# AmpliconSuite: an end-to-end workflow for analyzing focal amplifications in cancer genomes

**DOI:** 10.1038/s41592-026-03264-x

**Published:** 2026-10-16

**Authors:** Jens Luebeck, Ted Liefeld, Ivy Tsz-Lo Wong, Edwin Huang, Forrest Kim, Bhargavi Dameracharla, Michael Chan, Rohil Ahuja, Kyra Fetter, Dhruv Khatri, Kaiyuan Zhu, Thorin Tabor, Daniel Schreyer, Gino Prasad, Aditi Gnanasekar, Siavash Raeisi Dehkordi, Simone Spandau, Michał Adamaszek, Rishaan Kenkre, Tushar Agashe, Devika Torvi, Mădălina Giurgiu-Kraljič, Soyeon Kim, Hoon Kim, Peter Bailey, Roel G. W. Verhaak, Viraj Deshpande, Michael Reich, Paul S. Mischel, Jill P. Mesirov, Vineet Bafna

**Affiliations:** 1 Department of Computer Science and Engineering, University of California, San Diego, La Jolla, CA USA; 2 Department of Medicine, University of California, San Diego, San Diego, CA USA; 3 Department of Pathology, Stanford University School of Medicine, Stanford, CA USA; 4 Sarafan Chemistry, Engineering, and Medicine for Human Health, Stanford University, Stanford, CA USA; 5 Department of Bioengineering, University of California, San Diego, San Diego, CA USA; 6 School of Computer Science, Shanghai Jiao Tong University, Shanghai, China; 7 School of Cancer Sciences, University of Glasgow, Scotland, UK; 8 MOSEK ApS, Copenhagen, Denmark; 9 Department of Pediatric Oncology and Hematology, Charité – Universitätsmedizin Berlin, Berlin, Germany; 10 Department of Biopharmaceutical Convergence, Sungkyunkwan University, Suwon, Republic of Korea; 11 Botton-Champalimaud Pancreatic Cancer Center, Champalimaud Foundation, Lisbon, Portugal; 12 Department of Neurosurgery, Yale University School of Medicine, New Haven, CT USA; 13 The Bioinformatics Shop LLC, San Francisco, CA USA; 14 Mivax Therapeutics Inc., San Francisco, CA USA; 15 Moores Cancer Center, University of California, San Diego, San Diego, CA USA; 16 Halıcıoğlu Data Science Institute, University of California, San Diego, San Diego, CA USA; 17 Present Address: Sanford Burnham Prebys Medical Discovery Institute, La Jolla, CA USA

**Keywords:** Software, Cancer genomics

## Abstract

Here we present AmpliconSuite, a toolkit for focal amplification analysis from cancer whole-genome sequencing that identifies extrachromosomal DNA (ecDNA), breakage–fusion–bridges and other amplification modes. Its ecContext module categorizes ecDNA structures to understand formation mechanisms. Validation across 175 metaphase fluorescence in situ hybridization-probed genes in 72 models demonstrated accurate predictions. Analysis of 6,506 patient pan-cancer samples revealed ecDNA-mediated oncogene co-amplification preferences. To democratize data sharing, we created AmpliconRepository, a community-contributable platform, enabling investigation of results across >15,000 publicly hosted samples.

## Abstract (web summary)

AmpliconSuite is a toolkit that enables the analysis of focal amplifications from cancer whole-genome sequencing data.

## Main

Focally amplified oncogenes frequently drive cancer progression, yet different modes of amplification have distinct consequences^1^. Extrachromosomal DNA (ecDNA)-mediated amplifications are associated with high transcription^2^, copy-number (CN) adaptation and therapeutic resistance^1,3–5^. Distinguishing focal amplification modes using whole-genome sequencing (WGS) is bioinformatically challenging^6^.

**Fig. 1:**
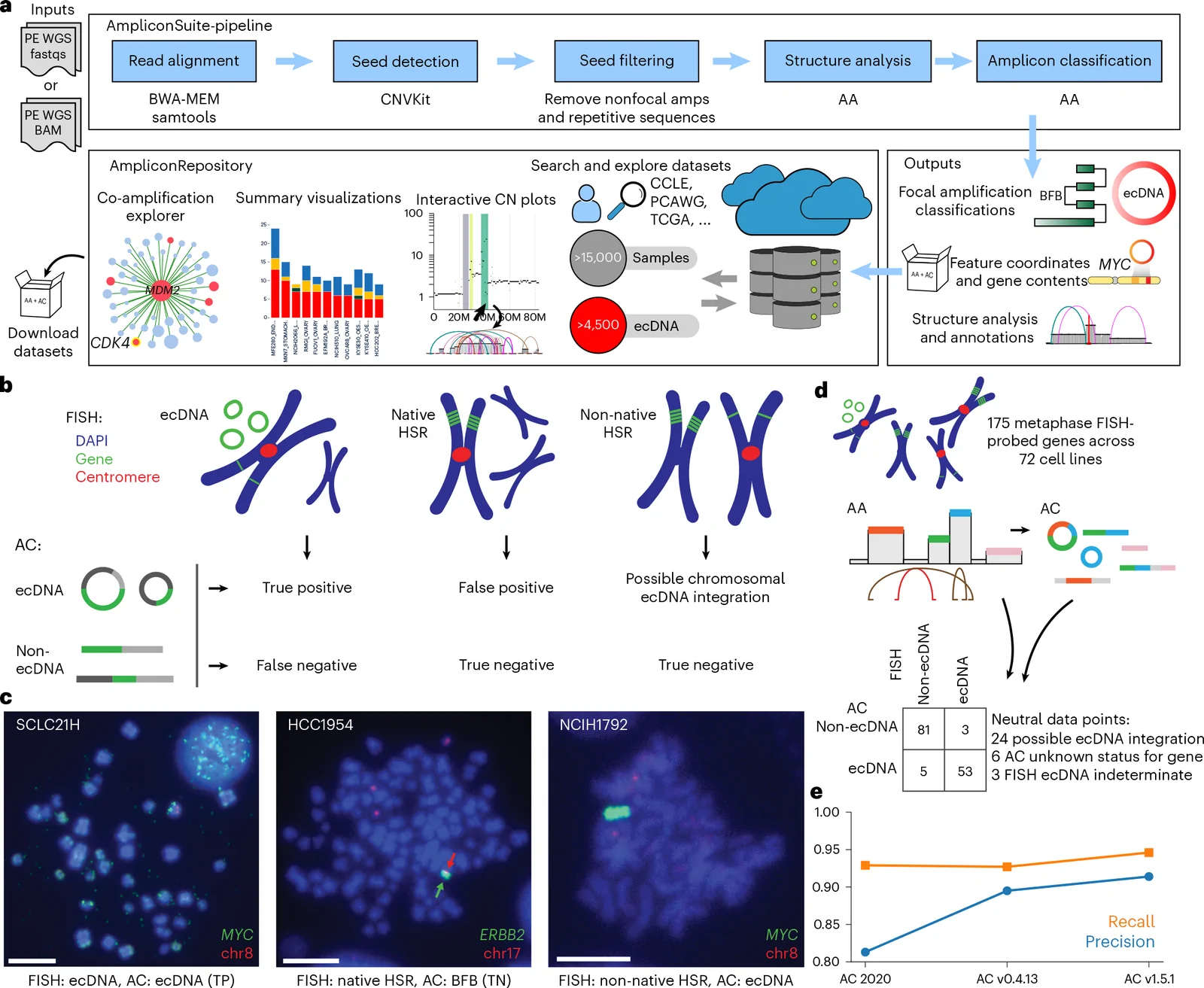
AmpliconSuite-pipeline, AmpliconRepository and FISH validation data. **a**, Schematic of AmpliconSuite-pipeline and AmpliconRepository. Users provide sequencing data to AmpliconSuite-pipeline, which optionally performs alignment and genome-wide CNV calling to identify candidate focal amplification regions. Filtered regions are passed to AA, which analyzes local genome structure; AC then reports the focal amplification class. Outputs can be uploaded to AmpliconRepository, where users query the database, interactively explore samples via Plotly and IGV plots, examine ecDNA gene co-amplifications and search or download results. PE, paired-end. **b**, Correspondence of AC and metaphase FISH findings, separated by HSR type, into truth labels. Colocation of probed gene and centromeric probe for original chromosome of gene indicates native HSR; probed gene without its native centromere indicates non-native HSR. **c**, Representative FISH images with probed gene and centromere, labeled with AC findings for three cancer cell lines. Scale bars, 10 µm. 17–24 images were captured per probed gene. TP, true positive; TN, true negative. **d**, Summary of validation data and AC/FISH agreement. Neutral data points include samples with possible ecDNA integration, ‘unknown’ AC categorization or indeterminate FISH ecDNA status. **e**, Precision and recall for previously published AC versions using FISH validation data. Source data

We present AmpliconSuite-pipeline, a workflow for focal amplification analysis, and AmpliconRepository, a community-sourced platform for results sharing, with >15,000 results publicly available (Fig. 1a). At AmpliconSuite’s core is AmpliconArchitect (AA)^7^, which jointly analyzes structural variants (SVs) and copy numbers (CNs) from WGS data. AmpliconSuite-pipeline combines input preparation, AA and AmpliconClassifier (AC), and is available through Bioconda^8^ and Nextflow^9^. We added a new method, ecContext, to AC, which predicts whether ecDNA is excisional or chromothripsis associated. A metaphase fluorescence in situ hybridization (FISH) dataset of 175 probed genes with matching WGS was assembled for validation.

## Results

### AmpliconSuite-pipeline

**Extended Data Fig. 1:**
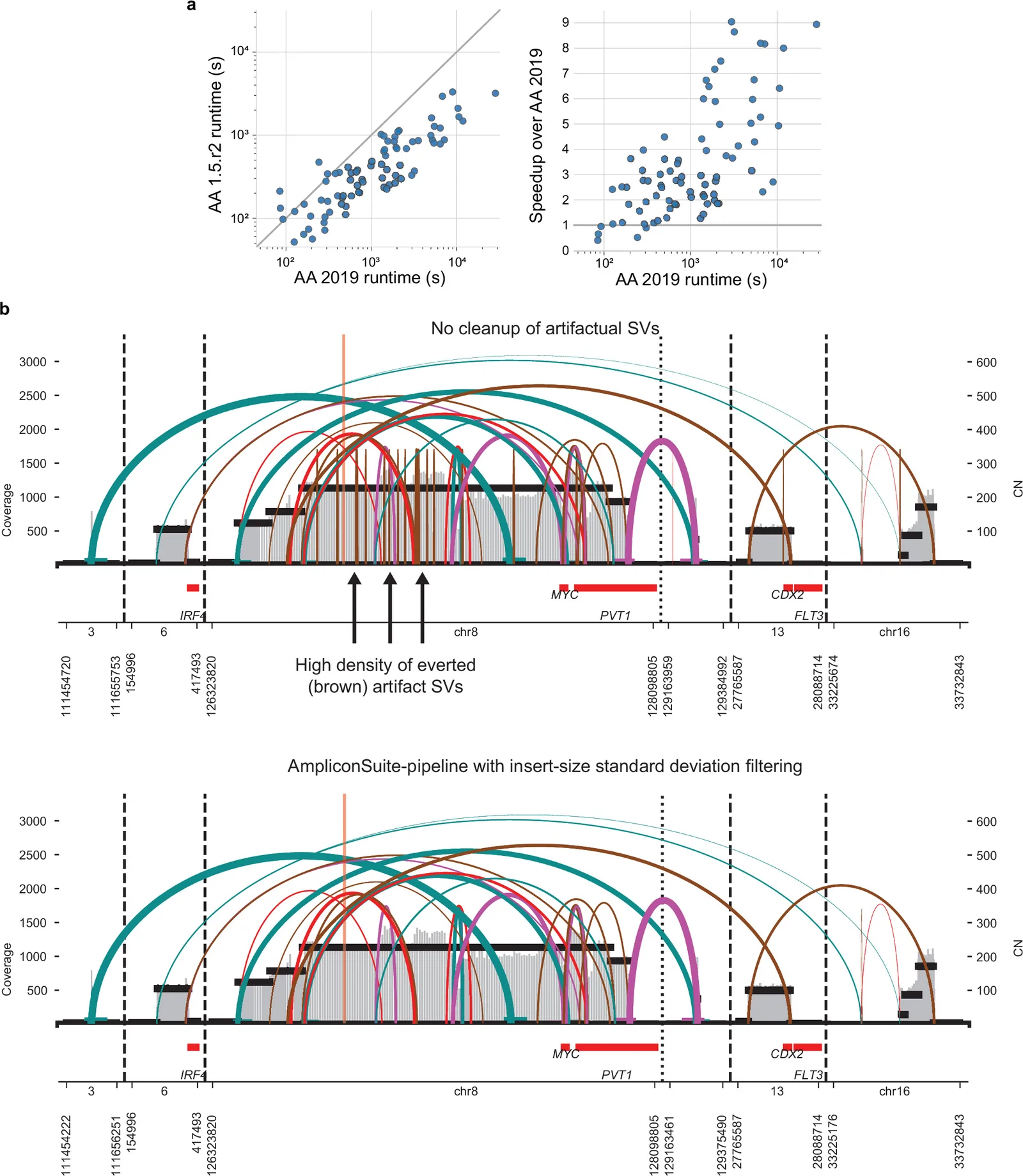
AA runtime improvement and artifact filtering. **a)** AmpliconArchitect (AA) runtimes on 81 cancer cell line samples comparing the original 2019 version and v1.5.r2. Diagonal line: y = x. Right panel shows speedup from these timing values; horizontal grey line at y = 1 indicates equivalent speed. **b)** AA Sashimi-style plots of copy number and identified SVs in COLO320DM (data from Wu et al., 2019). Top: default *--insert_sdevs* (3.0) showing artifactual SVs driven by high insert size variance, filtered by AmpliconSuite-pipeline. Bottom: *--insert_sdevs* 9.0, demonstrating user-adjustable stringency.

AmpliconSuite-pipeline predicts focal amplifications from FASTQ, BAM or CRAM input. Seed regions are identified (Methods) for AA, which constructs a CN-aware breakpoint graph^7^. We substantially improved AA’s performance since release (Methods). AA v1.5.r2 showed a 2.8 times median speedup over v1.0, increasing to 6.4 times for samples originally taking >2 h (Extended Data Fig. 1a and Supplementary Table 1). AmpliconSuite-pipeline detects low properly-paired read rates, adjusting AA’s parameters to reduce artifactual SVs (Extended Data Fig. 1b), and supports user-provided SV VCF files augmenting SV discovery.

**Extended Data Fig. 2:**
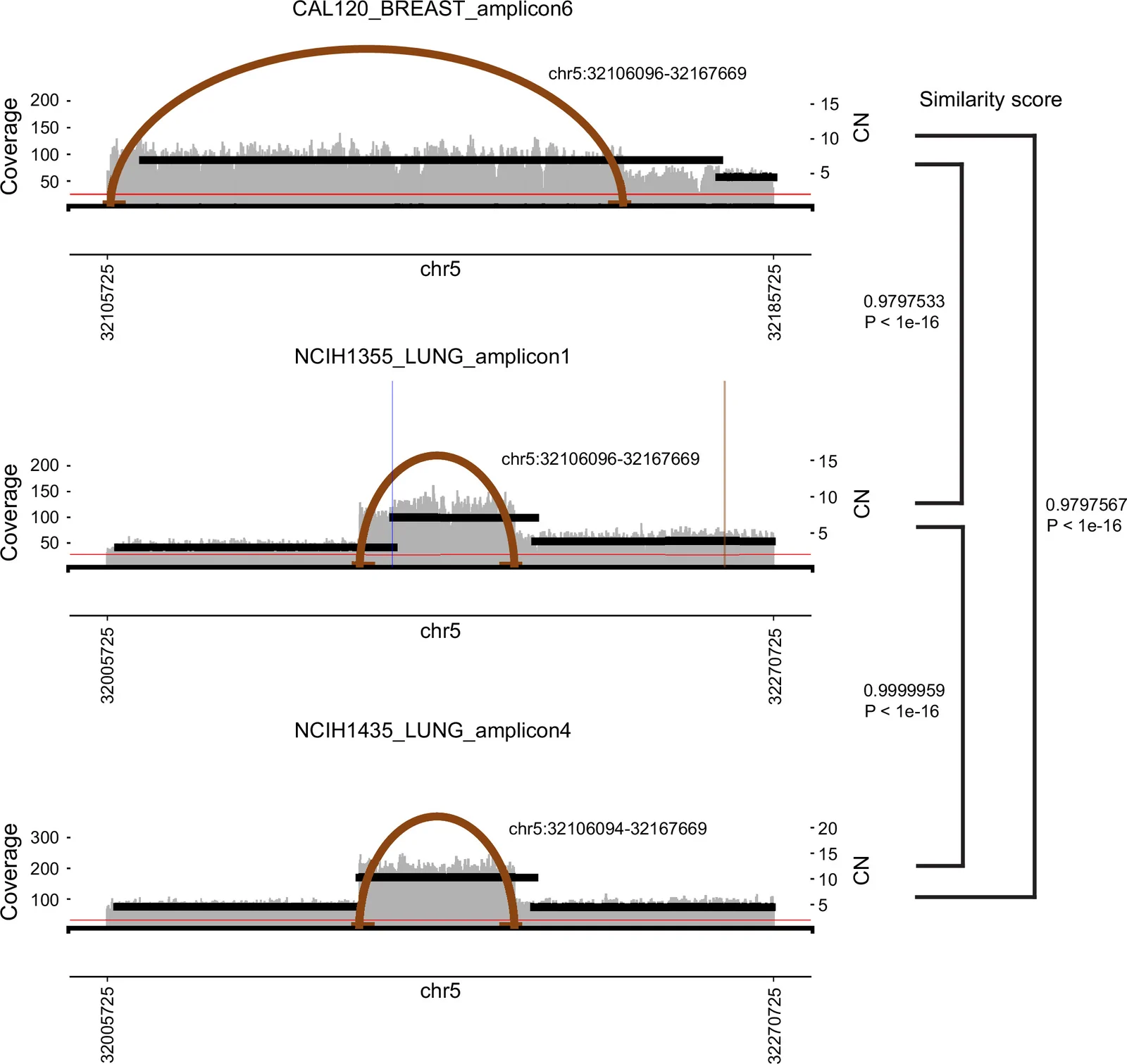
Similarity score filtering. AmpliconArchitect plots for three CCLE samples showing an identical ~61 kb structure at chr5:32.10-32.17 Mbp classified as linear amplification (CAL120_BREAST) or ecDNA (NCIH1355, NCIH1455). Similarity score analysis (right side) revealed high similarity in the amplification across these unrelated samples. The structure corresponds to a rare germline multi-copy duplication (2.2% population frequency; gnomAD-v4.1: DUP_CHR5_C02C1ED4), illustrating how similarity filtering identifies false positive focal amplifications due to population-level structural variation.

AC applies rule-based criteria to classify focal amplifications as ecDNA, breakage–fusion–bridge (BFB), complex noncyclic or linear amplifications^10^ (Methods). AC annotates focal amplification genes, lncRNAs, coordinates, complexity scores and applies ecContext to distinguish ecDNA mechanisms (Methods). An optional similarity score-based filter controls reference-derived false positives by computing similarity scores between amplicons^10^ (Methods). In Cancer Cell Line Encyclopedia^11^ (CCLE) data, similarity-score filtering revealed an identical ~61-kb amplified circular structure detected in three unrelated samples, representing a rare germline multi-copy duplication (2.2% population frequency; gnomAD-v4.1: DUP_CHR5_C02C1ED4)^12^ not ecDNA (Extended Data Fig. 2).

### Validation

We validated the methods with 175 metaphase FISH-probed genes across 72 cell lines having closely matching WGS (Supplementary Table 2), including 90 genes from five previous studies^1,4,13–15^. This FISH comprised 56 ecDNA-borne, 3 ecDNA-indeterminate, 52 non-native homogeneously staining region (HSR; an amplified gene on a different chromosome than its centromere probe), 39 native HSR and 25 unknown HSR (centromere unprobed) genes.

**Extended Data Fig. 3:**
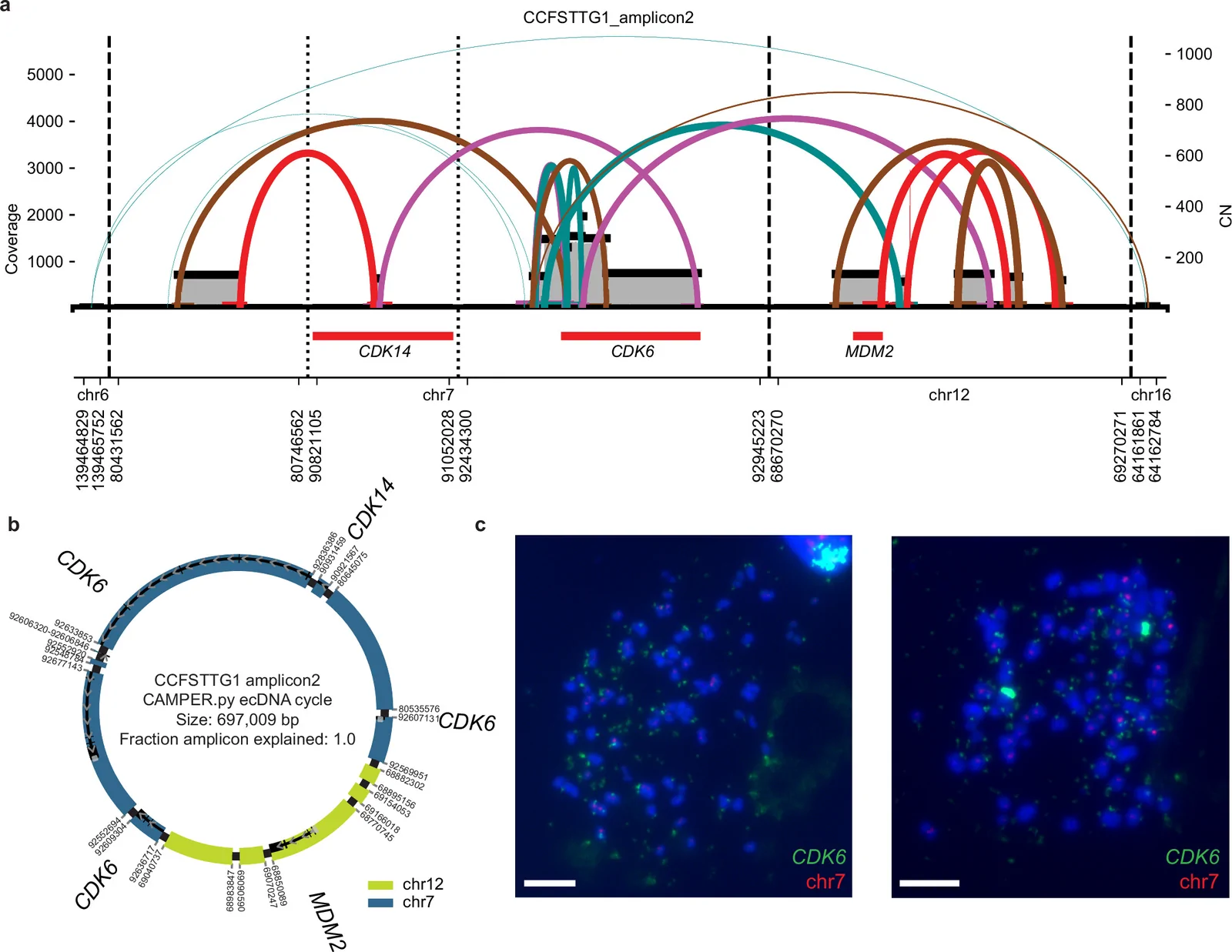
Mixed ecDNA and non-native HSRs in CCF-STTG1. **a)** AA sashimi plot for CCFSTTG1 ecDNA containing *CDK6*, *MDM2* and *CDK14*. **b)** CycleViz diagram using AmpliconSuite’s CAMPER.py cycle extraction (longest cycle conforming to estimated segment multiplicities) showing a 697kbp ecDNA structure which fully explains all amplified segments and their multiplicities, suggesting no additional overlapping unexplained amplification. **c)** Representative metaphase FISH images for CCFSTTG1 showing *CDK6* (green) as ecDNA (left) and a mix of ecDNA and non-native HSR (right; HSR does not co-locate with chr7 marker in red). Scale bars, 10 µm. 19 FISH images collected.

Complete integration of ecDNA into chromosomes is common in cancer cell lines^1,3,16,17^, appearing typically as a non-native HSR (24 non-native HSR genes with ecDNA WGS classification), characteristic of random integration or neochromosome formation^18^. Chromosomal mechanisms of amplification tended to remain on native chromosomes (eight of nine BFBs with a probed centromere were native HSRs). We therefore marked FISH:non-native HSR/AC:ecDNA cases as neutral in performance calculations (Methods and Fig. 1b–d). Importantly, ecDNA-derived HSRs may coexist latently^1^. In CCF-STTG1, WGS identified a single *CDK6* ecDNA cycle explaining the amplification (Extended Data Fig. 3a,b), yet 3 of 19 metaphase images also showed non-native *CDK6* HSRs (Extended Data Fig. 3c). *MYC* in DMS273 similarly appeared as both ecDNA and non-native HSR.

**Extended Data Fig. 4:**
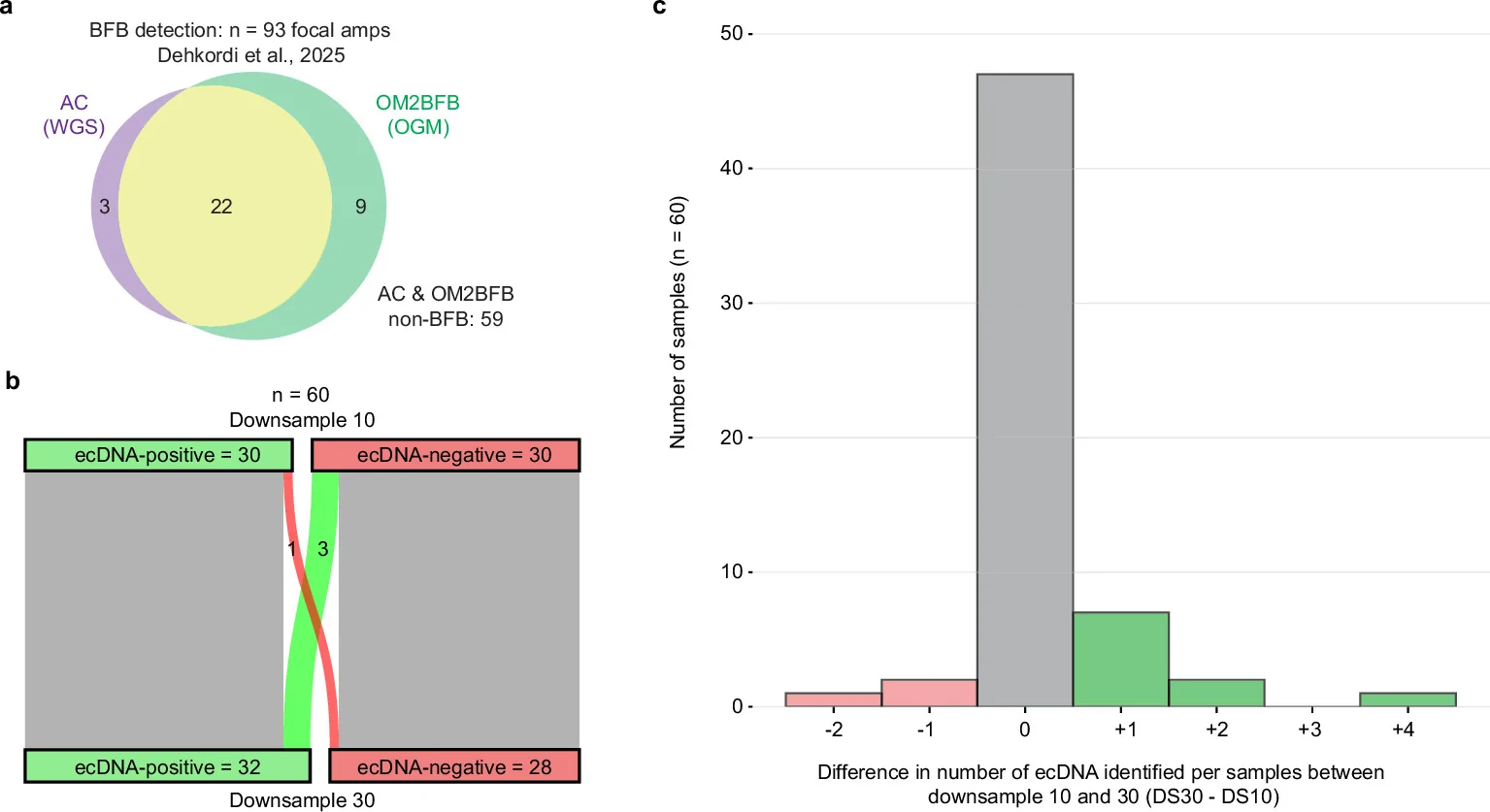
Benchmarking AC. **a)** Comparison of focal amplification class predictions for AC (using WGS) and OM2BFB (using optical genome mapping) on 93 BFB candidates. Numbers in each circle indicate BFBs predicted by each tool, and agreement on non-BFBs is shown outside the Venn diagram. **b)** Sankey diagram showing how the number of detected ecDNA changes between downsampling to 10x baseline coverage (default) and downsampling to 30x for 60 CCLE samples with at least >30x initial coverage, which all showed at least one focal amplification at default downsampling – and half showed at least one ecDNA. **c)** Histogram showing the difference in the number of ecDNA species discovered per sample between downsampling to 30x coverage and 10x coverage.

AC v1.5.1 applied to this dataset (Fig. 1d) achieved precision of 0.91 and recall of 0.95, improving over previous versions: 2020 (0.81 and 0.93, respectively) and v0.4.13 (0.89 and 0.93, respectively) (Fig. 1e). BFB performance was measured against an optical genome mapping dataset of 93 previously analyzed BFB candidates^15^, used as a ground truth, yielding recall of 0.71, specificity of 0.95 and precision of 0.88 (Extended Data Fig. 4a and Supplementary Table 3).

**Extended Data Fig. 5:**
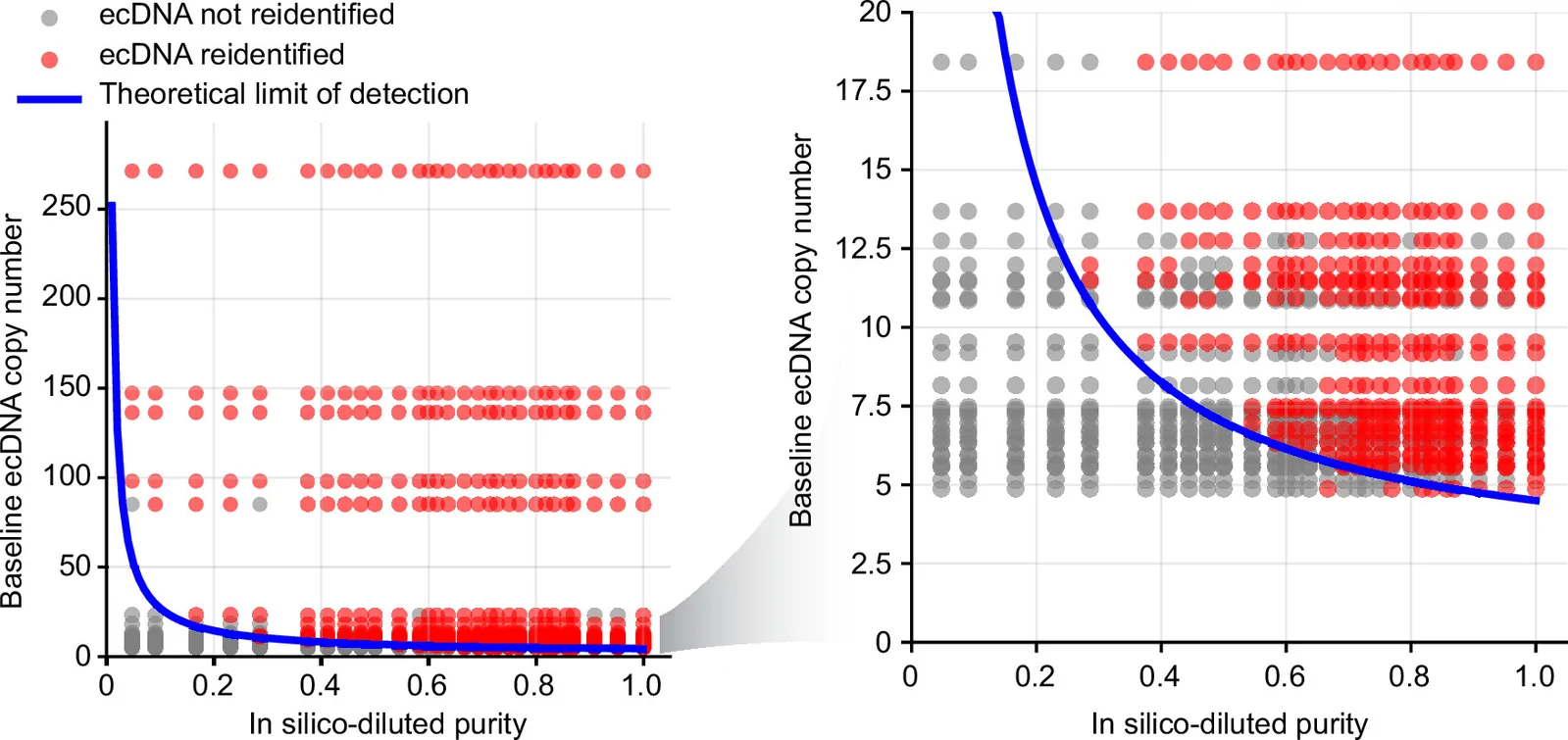
In silico purity dilution analysis. Scatterplot of ecDNA copy number against in silico-diluted purity, colored by whether the ecDNA was re-identified by AmpliconSuite-pipeline. 34 ecDNA species from nine ecDNA-rich cell lines are represented. In silico dilution levels ranged from 4.8% to 95.2% purity based on mixing cell line reads with NA12878 reads. The blue curve represents the theoretical detection threshold, where the median copy number of the genomic regions on ecDNA in the diluted mixture achieved at least 4.5 copies as a function of the purity, normal ploidy providing two copies.

In silico experiments tested parameter robustness, downsampling, simulated ecDNA recovery and tumor purity sensitivity (Methods). Sweeps across AC’s 19 configurable parameters confirmed defaults achieved near-optimal F1 scores against FISH validation data (Supplementary Table 4). AC was consistent across downsampling (10× and 30×) on 60 CCLE lines (30 with ecDNA and 30 with non-ecDNA focal amplifications), as three samples gained and one lost ecDNA status at higher coverage (Extended Data Fig. 4b,c and Supplementary Table 5). On simulated ecDNA-containing WGS^19^, AC recovered 89% (39/44) of chromothriptic and 83% (35/42) of excisional ecDNAs, substantially outperforming AC 2020 (46% and 62%, respectively; Supplementary Table 6). Purity dilution experiments with cancer cell line WGS and admixed normal reads on 34 unique ecDNAs from nine cell lines diluted across 4.8–95.2% purity recovered 79.5% of theoretically detectable ecDNAs across levels (Methods, Extended Data Fig. 5 and Supplementary Table 7).

### AmpliconRepository

**Extended Data Fig. 6:**
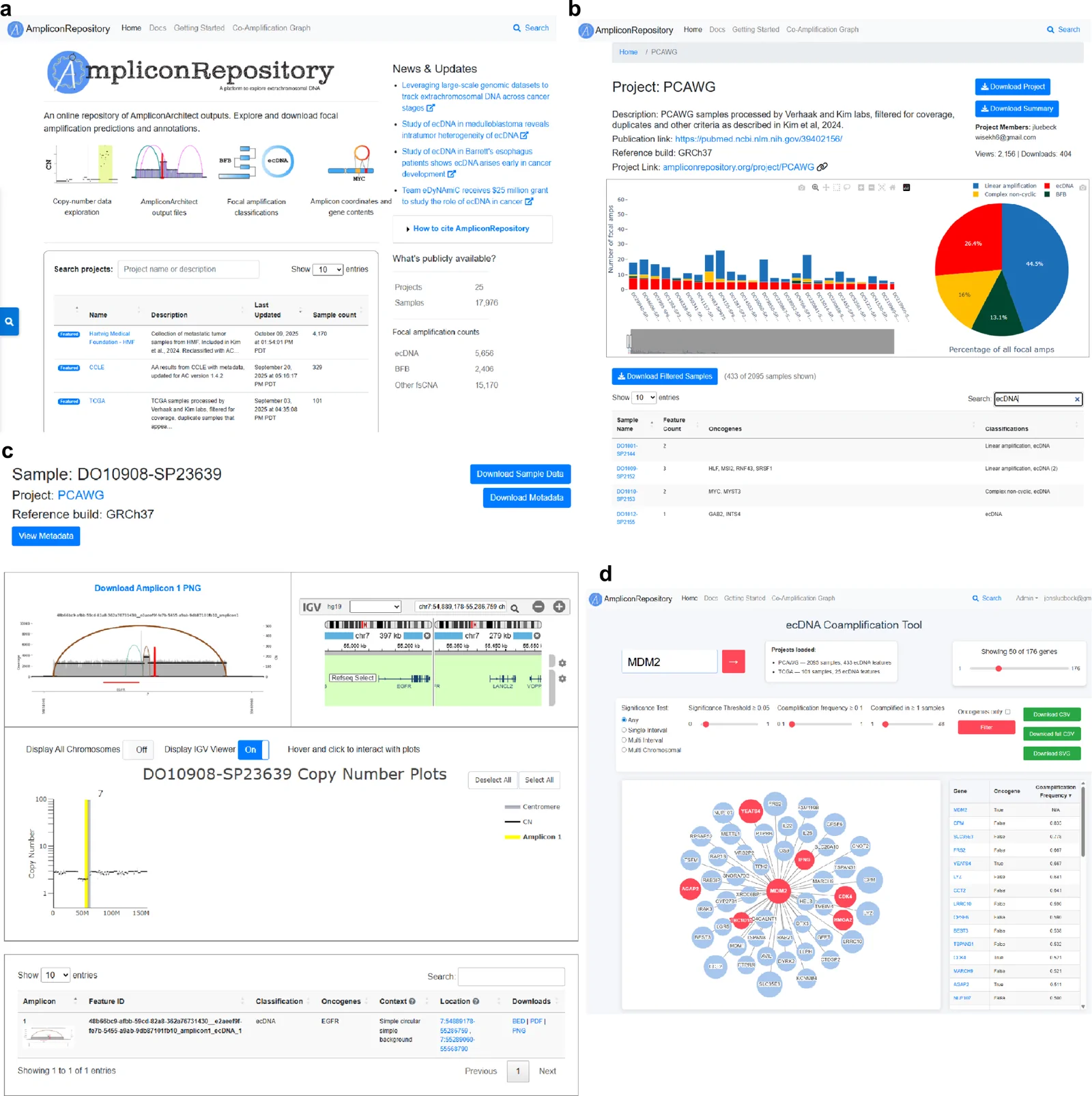
AmpliconRepository.org. **a)** Screenshot of the AmpliconRepository.org homepage showing a table of featured, public, and private projects (private projects shown only when logged in), summary of available data, and entry points to site search (left icon or top bar). **b)** Project page with Plotly-based visualizations of the number and class of focal amplifications per sample, a pie chart of overall class frequencies, and download links for a tarball of all outputs and a summary table. A searchable table below lists samples, links to each sample page, and allows filtering for subset download. **c)** Sample page with buttons for downloading sample files and metadata, and an interactive Plotly-based copy-number visualization where users can click highlighted amplicon regions to display AA sashimi plots and toggle an IGV gene viewer. A summary table below lists focal amplification features. **d)** ecDNA co-amplification graph (top navigation bar): a Cytoscape.js visualization of ecDNA-mediated gene co-amplifications across selected projects. The top input bar accepts gene queries; a control panel adjusts edge display requirements; a summary table on the right shows co-amplification frequencies, with tabular download via right-hand buttons.

To democratize data sharing, we created AmpliconRepository (https://www.ampliconrepository.org) (Methods and Extended Data Fig. 6a). AmpliconRepository currently hosts >15,000 publicly available results. Users create projects publicly or privately from uploaded AmpliconSuite-pipeline outputs. Project pages host overviews of focal amplification abundance (Extended Data Fig. 6b), and sample pages display interactive CN profiles (Extended Data Fig. 6c). A site-wide search enables custom batch downloads. To demonstrate utility, we analyzed published^20^ results obtained from AmpliconRepository, spanning 6,506 patient samples across ICGC (TCGA and PCAWG), HMF and GLASS projects. AC v1.5.1 identified 2,413 predicted ecDNA, with 36.0% (534) of ecDNA-positive samples showing multiple ecDNA.

### ecDNA-mediated gene co-amplifications

**Fig. 2:**
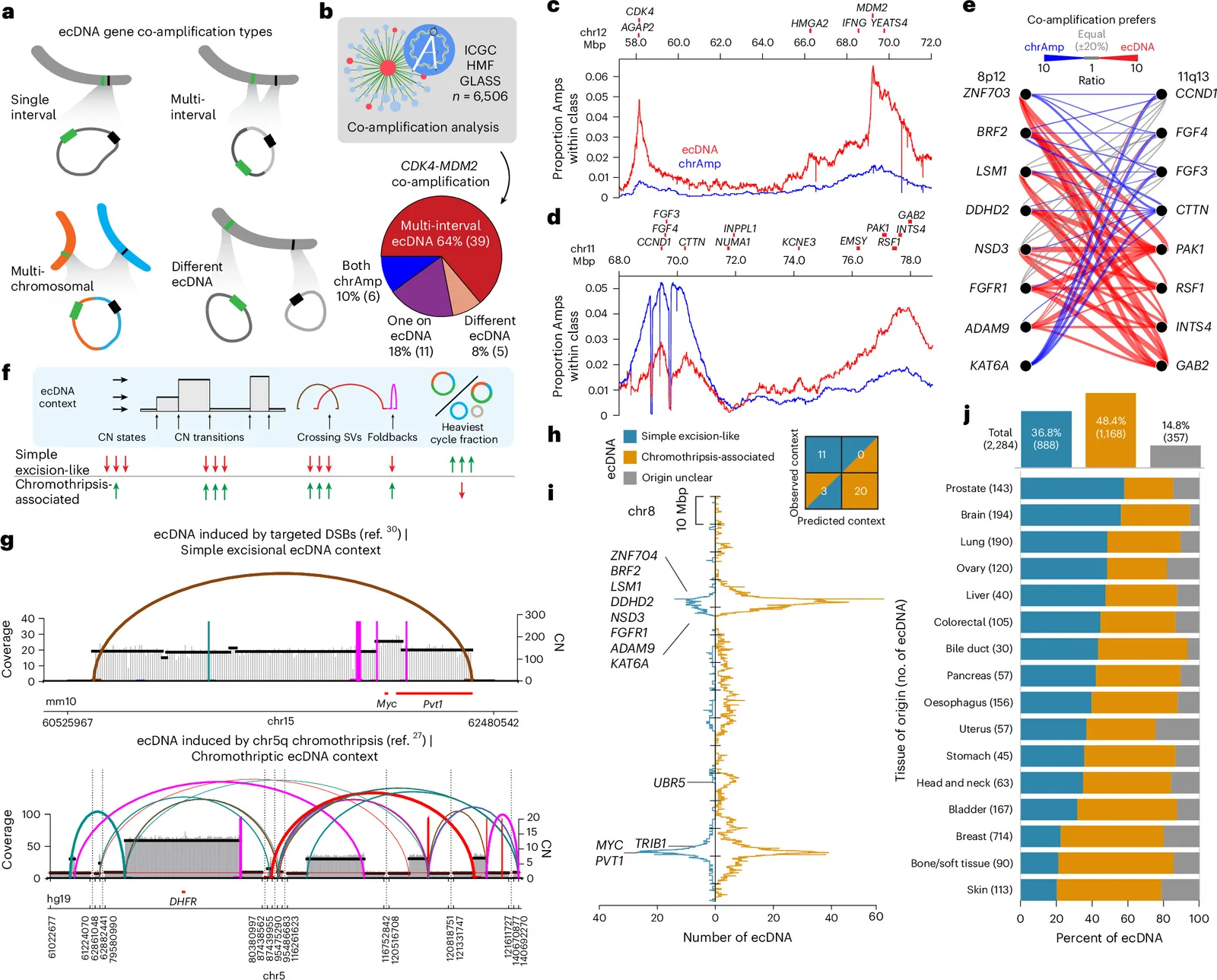
Analysis of focal amplifications in pan-cancer datasets. **a**, Diagram of ways two genes can become co-amplified on ecDNA in a single sample. **b**, The AmpliconRepository co-amplification utility characterizes co-amplification types for 61 *CDK4*-*MDM2* events in a combined pan-cancer cohort (ICGC, HMF and GLASS) used throughout the figure. **c**, Relative proportions of ecDNA and chrAmp events across a chr12 region containing *CDK4* and *MDM2*, highlighting ecDNA’s selective preference for these and neighboring oncogenes. **d**, Relative proportions of amplification types across a chr11q focal amplification hot spot, showing *CCND1* enrichment among chrAmps and ecDNA-frequent amplification of an oncogene cluster around 76–78 Mbp. **e**, Frequently co-amplified oncogene pairs from chr8p12 and chr11q13, colored by chrAmp or ecDNA preference (gray: ratio within ±20%; line thickness proportional to ratio strength). **f**, Cartoon of signal types used by ecContext to distinguish excisional from chromothripsis-associated ecDNA. **g**, AA sashimi plot examples for ecDNA induced in cancer models, labeled by predicted ecContext category. **h**, Confusion matrix of ecContext performance against observed labels in experimentally induced ecDNA from Pradella and Shoshani studies. **i**, Frequency of ecDNA by excision or chromothripsis for chr8. **j**, Percentages of simple excisional or chromothripsis ecContext label groupings by cancer type. Source data

In this pan-cancer data, ecDNA carried 1,664 oncogenes across 5,348 total megabase pairs (Mbp), 2.5 times the oncogene density found in the GRCh37 reference (997 oncogenes across 3,096 Mbp). We hypothesized that gene pairs frequently co-amplified on ecDNA would reveal selective pressures shaping ecDNA evolution^14,21^. Genes co-amplify on ecDNA through different mechanisms (Fig. 2a): from a single interval, from noncontiguous intervals, from intervals of different chromosomes or on distinct ecDNA. To explore, we implemented a queryable co-amplification explorer on AmpliconRepository (Extended Data Fig. 6d).

*CDK4*-*MDM2* co-amplification (61 samples) was predominantly ecDNA driven: 64% (39/61) by multi-interval ecDNA, 8% (5/61) by different ecDNA, 18% (11/61) with one gene by ecDNA and the other by chromosomal focal amplification (chrAmp), and 10% (6/61) by chrAmps (Fig. 2b). Across chr12q14–15 (57–72 Mbp) (Fig. 2c), ecDNA typically excluded the intergenic region between *CDK4* and *MDM2*. However, the chromatin remodeling factor *YEATS4*, located 514 kilobase pairs (kbp) downstream of *MDM2*, was retained alongside *MDM2* in 79% (77/97) of *MDM2*-ecDNA samples. Conversely, the upstream immunomodulatory gene *IFNG* (648 kbp upstream) showed lower co-inclusion (38%, 37/97), suggesting that context-dependent pressures shape ecDNA co-amplification partners.

Analysis of chr11q13 (68–79 Mbp) revealed a co-amplification hot spot (Fig. 2d). *CCND1*, *FGF3*, *FGF4* and *CTTN* were frequent chrAmp targets *(CCND1*: 76% chrAmp, 131/172). By contrast, 57% of focal amplifications covering the ~1-Mbp subregion containing *PAK1*, *RSF1*, *INTS4* and *GAB2* amplified through ecDNA. Among those, 66% (48/73) showed ecDNA amplified all four genes together—suggesting selective advantages for synergy between signaling pathway components and chromatin remodeling machinery.

The most frequent multi-chromosomal co-amplifications paired chr8p12 and chr11q13 oncogenes, and preferentially via ecDNA (Fig. 2e), frequently combining signaling oncogenes (*FGFR1* and *PAK1*) with chromatin remodeling factors (*RSF1* and *NSD3*/*WHSC1L1*). This reveals ecDNA as a capable substrate for combining distant, complementary oncogenes.

### ecContext classifies ecDNA formation mechanisms

**Extended Data Fig. 7:**
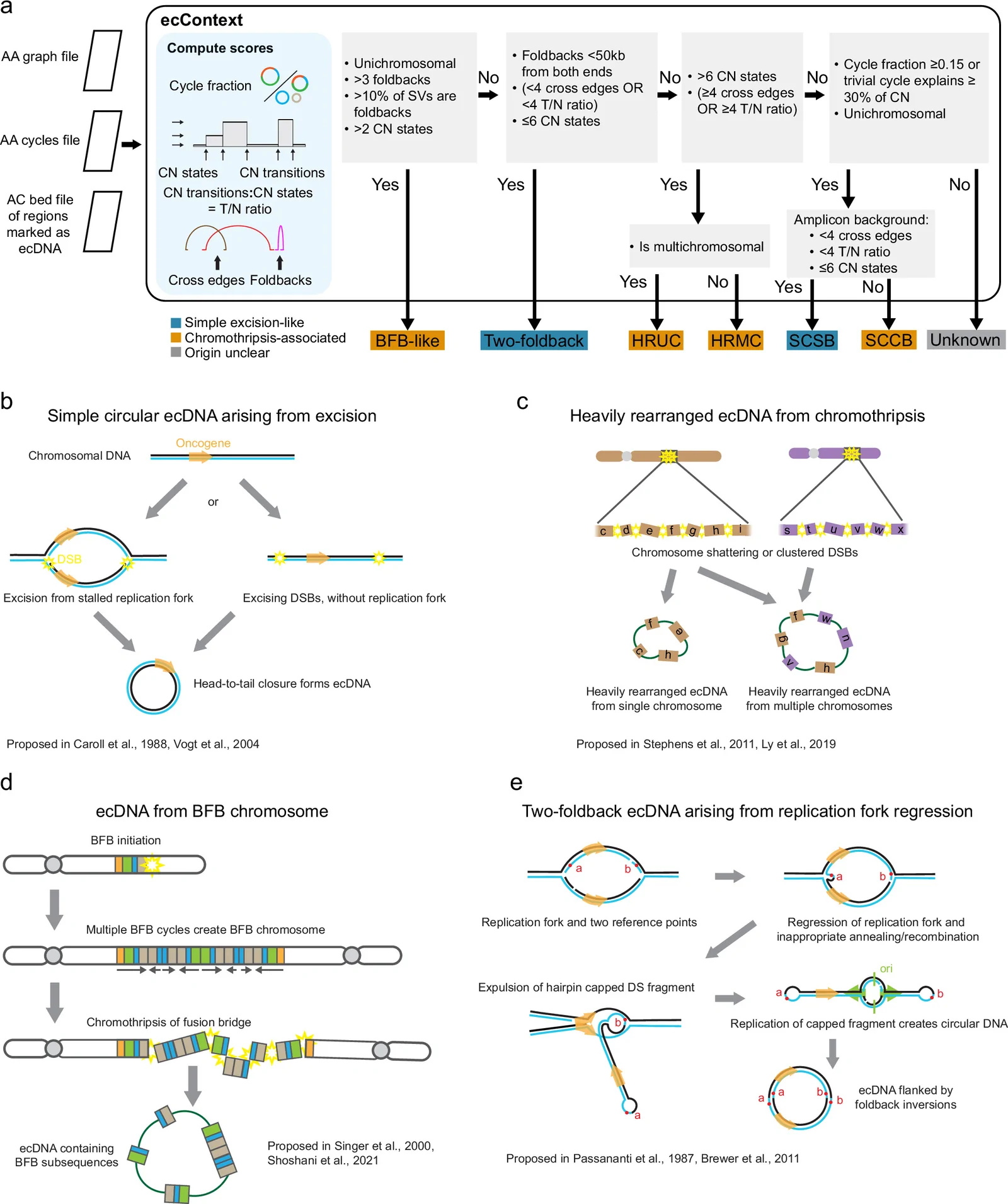
ecContext and ecDNA formation mechanisms. **a)** Decision diagram for ecContext, which takes the AA graph and cycles files plus the AC-called ecDNA regions BED file as input. ecContext computes scores from these inputs to predict ecDNA context: BFB-like, two-foldback, heavily rearranged unichromosomal (HRUC), heavily rearranged multichromosomal (HRMC), simple circular simple background (SCSB), simple circular complex background (SCCB), or unknown. **b)** Model of ecDNA formation by excision, consistent with Carroll et al., 1988 and Vogt et al., 2004. A DNA fragment may be excised from a replication fork in crisis or by two double-strand breaks on pre- or post-replicated DNA. Head-to-tail ligation forms a simple circular ecDNA. Additional rearrangements before or after formation are possible. **c)** Model of ecDNA formation by chromothripsis - shattering or clustered breakage of genome fragments from one or few chromosomes, as proposed in Stephens et al., 2011 and Ly et al., 2019. While shattering alone does not imply amplification, circular repair of fragments can yield ecDNA spanning one or multiple chromosomes. **d)** Model of ecDNA formation from a breakage-fusion-bridge (BFB) chromosome precursor, consistent with Singer et al., 2000 and Shoshani et al., 2021. A BFB chromosome may break apart through chromothripsis or multi-focal breakage during cell division as the dicentric bridge is pulled apart. As in (c), circular repair can yield ecDNA containing BFB-derived rearrangements. **e)** Model of ecDNA formation by replication fork regression, consistent with Brewer et al., 2011 (‘ODIRA’) and Passananti et al., 1987. Fork regression followed by annealing or recombination of the loose ends of newly synthesized DNA can yield a hairpin-capped double-stranded fragment expelled from the fork. Subsequent replication from an internal origin yields a circular molecule with an inverted repeat, producing the foldback SV bookends observed in sequencing data.

**Extended Data Fig. 8:**
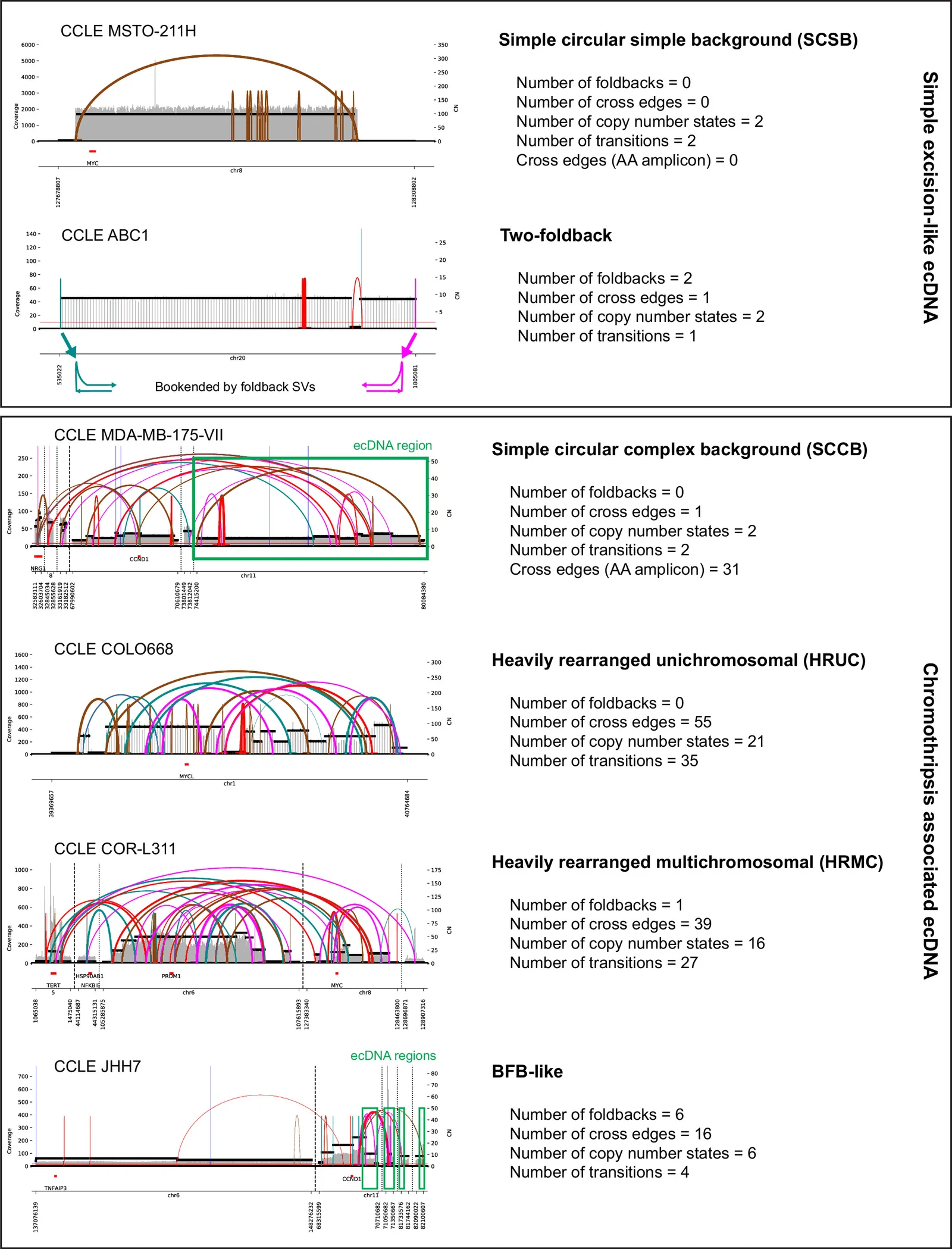
ecContext worked examples. Examples of the six ecDNA context categories (excluding ‘unknown’), showing the scores ecContext uses to assign labels.

To classify ecDNA formation mechanisms, we developed ecContext, which categorizes ecDNA on SV and CN patterns (Methods and Fig. 2f). Based on examined ecDNA structures, we established seven structural categories that ecContext emits: simple circular simple background (SCSB), simple circular complex background (SCCB), heavily rearranged unichromosomal (HRUC), heavily rearranged multi-chromosomal (HRMC), BFB-like, two-foldback and unknown (Extended Data Fig. 7a), which were mappable to proposed formation models^22–29^ (Extended Data Fig. 7b–e). Broadly, these encompass simple excisional mechanisms (SCSB, two-foldback) and chromothripsis-associated mechanisms (SCCB, HRUC, HRMC and BFB-like). Simple excisional categories exhibit fewer CN states, CN transitions, crossing SVs and foldback inversions, along with a more dominant heaviest cycle than chromothripsis-associated categories (Extended Data Fig. 8).

We validated ecContext using ecDNA launcher model systems (Fig. 2g). Pradella et al.^30^ generated simple megabase-scale ecDNA via Cre-loxP excision of murine *Myc* or *Mdm2*. Shoshani et al.^27^ demonstrated chromothripsis-associated *DHFR* ecDNA genesis in methotrexate-treated cells. ecContext correctly labeled all 11 excisional ecDNA as SCSB (8/8 Pradella, 3/3 Shoshani ‘simple DM’ labeled). 87% (20/23) of Shoshani chromothripsis-derived ecDNA capturing *DHFR* received chromothripsis-associated labels (HRUC and SCCB), demonstrating concordance between predicted and observed mechanisms (Fig. 2h and Supplementary Table 8).

### Formation mechanisms vary by genomic location and cancer type

**Extended Data Fig. 9:**
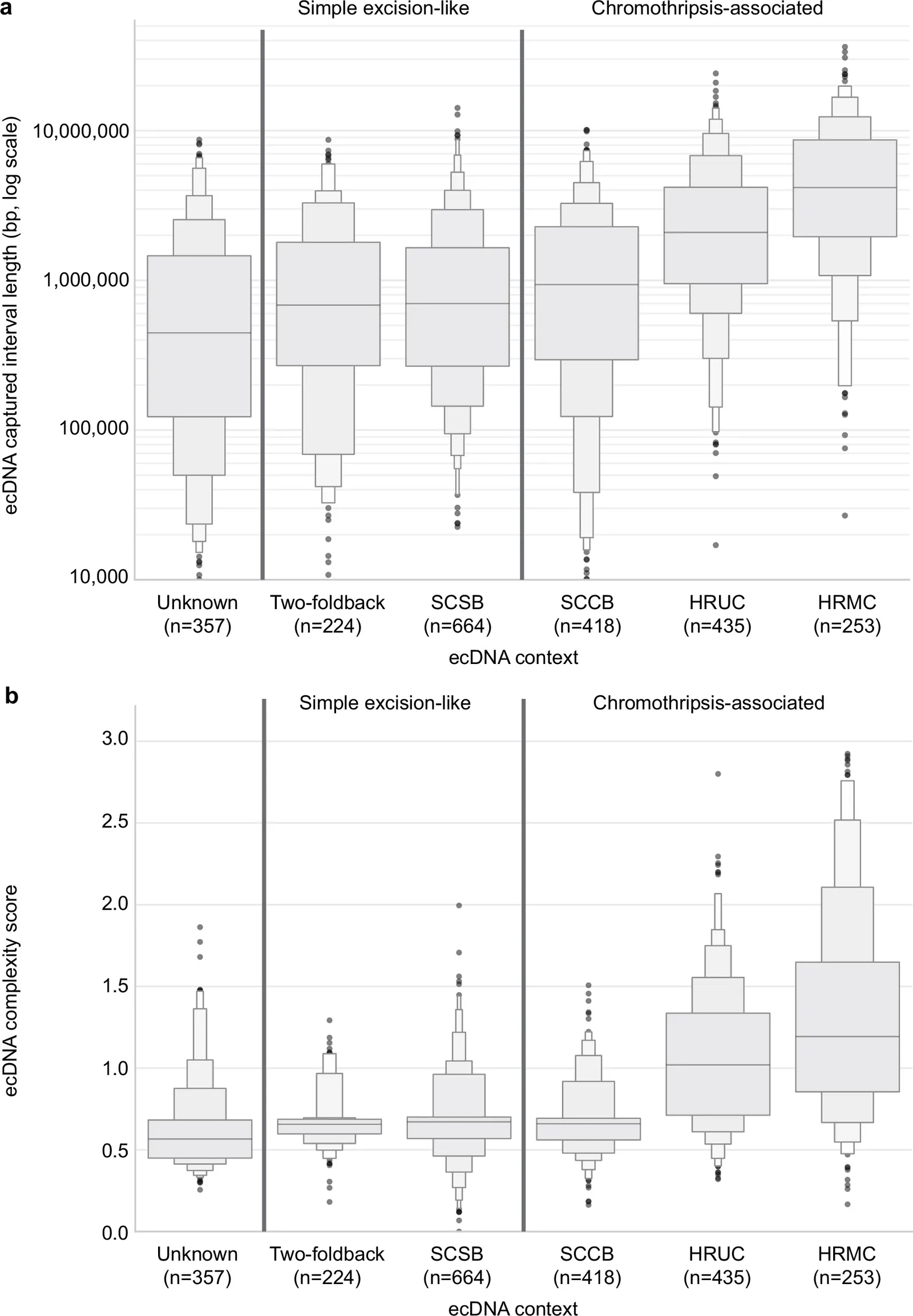
ecDNA size and complexity by ecContext label. **a)** Distribution of captured interval lengths (amplicon sizes with respect to reference genome) measured in base pairs for all ecDNA in each ecDNA context category. SCSB: simple circular simple background, SCCB: simple circular complex background, HRUC: heavily rearranged unichromosomal, HRMC: heavily rearranged multichromosomal. **b)** Distribution of ecDNA complexity scores for all ecDNA in each ecDNA context category. Source data

Applying ecContext to pan-cancer data revealed 36.8% (888) of ecDNA were simple excisional, 48.4% (1,168) were chromothripsis associated and 14.8% (357) had an unclear origin. Along chr8, the *FGFR1*-associated oncogene cluster preferentially associated to chromothripsis ecDNA, while *MYC* locus ecDNA was more balanced between mechanisms (Fig. 2i). ecDNA showed significantly greater variability in size than chrAmps (standard deviation, ecDNA: 3.22 Mbp, chrAmp: 2.23 Mbp, *P* < 1 × 10^−16^, Bartlett’s test). ecDNA size distributions varied by formation mechanism: simple excision-like ecDNA were smaller (medians, two-foldback 0.68 Mbp, SCSB 0.70 Mbp), while chromothripsis-associated ecDNA categories showed increased size (medians, SCCB 0.94 Mbp, HRUC 2.09 Mbp, HRMC 4.17 Mbp) (Extended Data Fig. 9a). Structural complexity scores followed the same pattern, with simple excision-like ecDNA (medians, two-foldback 0.66, SCSB 0.67) having lower complexity than chromothripsis-associated ecDNA (medians, SCCB 0.66, HRUC 1.02, HRMC 1.19) (Extended Data Fig. 9b). Formation mechanism frequencies varied substantially by cancer type. Prostate and brain cancers showed enrichment for simple excision-like ecDNA, while skin cancers and sarcomas showed more chromothripsis-associated ecDNA (Fig. 2j). These patterns suggest that tissue-specific factors influence the ecDNA formation mechanism and architecture.

## Discussion

AmpliconSuite provides a toolset to study focal amplifications including ecDNA, paired with a platform for sharing results. AmpliconSuite’s interoperable file formats support forthcoming integration of long-read methods such as CoRAL^19^ alongside other emerging tools within the ecosystem. The FISH dataset we assembled provides an orthogonal benchmark essential for refining methods to distinguish focal amplification mechanisms.

AmpliconRepository’s democratized data sharing enables new discoveries, and we anticipate continued growth beyond the >15,000 available samples. While other ecDNA databases exist^31–36^, AmpliconRepository differs in important ways: it provides complete tool outputs and lets contributors directly create, update and expand their projects.

ecDNA’s relationship with *CDK4*-*MDM2* co-amplification has important clinical implications^37^. ecDNA enables rapid modulation of both CNs, potentially contributing to therapeutic resistance, bridging distant genomic regions and selectively incorporating chromatin remodelers. Notably, some *CDK4*-*MDM2* amplifications cytogenetically appearing as chromosomal (for example, neochromosomes) probably derive from ecDNA intermediates^18^, suggesting ‘ecDNA competence’ and potential re-establishment of ecDNA with selective pressure. A key limitation of bulk WGS remains in distinguishing ecDNA from chromosomal ecDNA-derived HSRs, as the SVs indicating ecDNA origins persist. Nonetheless, identifying ecDNA-competent cells is biologically and clinically informative, as ecDNA-bearing subclones may persist and reselect^3^. Given the dynamics between extrachromosomal and integrated states, concurrent gathering of cytogenetic and sequencing data is essential.

Future work on ecDNA co-amplification dynamics and formation mechanisms, including expanding ecContext to understand mechanistically unclear ecDNA, has the potential to improve therapeutic strategies and accelerate the identification of new targets exploiting unique properties of focal amplifications.

## Methods

### Improvements to AA

SV detection was enhanced through entropy filtering of SV-end sequence content (Shannon entropy ≥0.75) to reduce false positives from low-complexity regions and stricter mapping quality requirements (≥1 read per SV end with mapping quality ≥20). Additional improvements include Python 3 compatibility, updated dependency support (MOSEK version ≥8), refined visualizations and deterministic outputs for reproducibility. A read caching strategy was added: when looking up the breakend destination of a read pair supporting an SV, all reads around that position are cached, as one SV endpoint in a region increases the likelihood of others nearby. Support was also added for GRCh38, mouse mm10 and GRCh38_viral (containing common oncoviral sequences; see the ‘Data repo construction’ section). To benchmark speed improvements, we ran a runtime comparison using low-coverage (median ~1×) WGS data from 81 cancer cell line samples with focal amplifications originally analyzed by AA^7^ on the same system (Intel Xeon CPU E5-2643 v2 3.50 GHz).

### Seed region filtering

Before running AA, AmpliconSuite-pipeline filters whole-genome CN variation (CNV) calls to identify candidate focal amplification regions (‘seed regions’). This step uses CNVkit^38^, by default, although other callers may be used assuming they do not undersegment the genome.

We define the following terminology:

- Copy number segment: a segment *S* of the reference genome with an assigned CN *S*_CN_. We denote the genomic length of the segment as |*S*|.
- Chromosome (arm) CN: the median CN of genome segments on a specific chromosome (arm). Denoted *C*_CN_ and *A*_CN_ for chromosome and chromosome arm CNs, respectively.
  The following parameters are used during the seed filtering:
- CN_cutoff: the minimum CN required for a region to be considered significantly amplified, assuming the baseline CN is 2. The default value is 4.5.
- Size_cutoff: the minimum size of a candidate seed region. The default value is 50 kbp.

Seed regions are identified in two phases: first the ‘prefiltering’ phase, which ensures that CNs are significantly amplified compared with the chromosome and, second, filtering based on repetitive sequence content and overlap with known high signal regions of the reference genome.

Prefiltering computes the median arm CN (*A*_CN_) per chromosome and removes amplifications consistent with karyotypic abnormalities, which are not focal amplifications. For each CN segment, *S* segments longer than 30 Mbp are discarded (unlikely to be focal). For |*S*| > 20 Mbp, CN_cutoff is doubled to prevent small-scale karyotypic abnormalities from being called focal.

The prefilter also handles CN segments falling into long stretches of continuously high CN despite being split across multiple segments. It identifies ranges of CN segments with a combined length of 10–20 Mbp where *S*_CN_ > CN_cutoff and neighboring segments are within 300 kbp of unamplified material. For segments in these ranges, CN_cutoff is set to 1.5× its default to avoid calling small-scale karyotypic events.

The prefilter then tests each segment against

$${S}_{\mathrm{CN}} > {A}_{\mathrm{CN}}+\mathrm{CN\_cutoff}-2,$$

merging neighboring segments with the same CN. The collection of regions passing these filters we termed the ‘prefiltered regions’.

AmpliconSuite-pipeline then passes the prefiltered regions to AA’s amplified_intervals.py utility^7^, First, the amplified_intervals.py script computes coverage statistics from the BAM file to determine *C*_CN_ (an independent sanity check on the provided CNV calls) and applies

$${S}_{\mathrm{CN}} > {C}_{\mathrm{CN}}+\mathrm{CN\_cutoff}-2.$$

Finally, amplified_intervals.py evaluates seed sequence content and overlap with reference genome problem regions. A candidate is filtered if >10% of its sequence overlaps conserved CN gain regions (see the ‘Data repo construction’ section) and <2 Mbp lies outside those regions. The method then checks sequence uniqueness, requiring the mean 35-bp *k*-mer uniqueness score (sliding window) to not exceed 2.5 occurrences elsewhere in the genome^7^. For non-oncoviral candidates, it also verifies that the region remains amplified after accounting for known segmental duplications, checking if

$${S}_{\mathrm{CN}}\times \frac{\left|S\right|}{\left|S\right|+\left|D\right|} > \mathrm{CN\_cutoff},$$

where |*D*| is the total length of the segmental duplication regions overlapping the candidate seed region.

Candidate seeds passing these filters are then merged if closer than 300 kbp. Merged non-oncoviral candidate seeds are then filtered by Size_cutoff, with final seeds reported as a BED file suffixed ‘AA_CNV_SEEDS.bed’. Users may bypass these filters by providing custom seed regions in a CNV BED file with that suffix.

### AC

We previously introduced methods to classify focal amplifications from AA outputs in Kim et al.^39^, later adding similarity scoring and amplicon complexity scoring in Luebeck et al.^10^. AC takes AA’s graph and cycle files (describing CN/SV properties and recovered genome structures, respectively) and applies rules to predict the focal amplification type. The complete description of rules previously described^10^, however, we summarize below.

The two inputs to AC are:

1. The AA breakpoint graph file (SV breakpoint edge locations and segment CNs).
2. The AA cycles file (cyclic and noncyclic paths in the breakpoint graph and their associated CNs).

First AC excludes genome paths on the basis of size (<10 kbp, excepting viral contigs), overlap with low-complexity or repetitive regions, or CN (CN <4.5 for included genome regions). Paths assigned a low copy count by AA’s decomposition are also filtered (typically fewer than three copies, depending on path length and segment CN). For retained paths, AC calculates a length-weighted CN (*W*) and proceeds with classification. AC first checks for BFB cycles, considering the fraction of SV edges (‘discordant edges’) with foldback orientation (duplicated inversion with <25 kbp between ends), and the fraction of segment pairs in decomposed paths joined by foldbacks. The amplicon is not considered a BFB if: the foldback-joined segment pair proportion is low (<0.295); fewer than three foldbacks exist and the foldback fraction across all SVs is <0.25; or foldback-containing paths represent <60% of total weight. As sequencing artifacts can also take foldback orientation, a valid BFB must contain no more than 15 foldback edges comprising no more than 80% of total amplicon SVs. Amplicons failing this filter are evaluated for complex noncyclic or linear amplifications. Otherwise, AC marks all paths consistent with BFB as BFB-like.

On remaining paths, AC assesses ecDNA presence by looking for cyclic paths with CN >4.5, lengths >50 kbp or at least 12% of the graph’s weight (segment lengths × CN) explained by cycles. AC differentiates ecDNA from linear tandem duplications using amplicon size, CN and structural complexity: small circular amplicons (<500 kbp) with simple head-to-tail SVs and modest CN gains (≤2.25-fold above background) are classified as linear, while high-CN circular amplicons showing extensive breakpoint reuse—a hallmark of ecDNA replication—are classified as ecDNA.

If neither BFB nor ecDNA is found, AC examines paths for complex noncyclic or linear amplifications. A complex noncyclic label is assigned if either: (1) the fraction of total weight in rearranged noncyclic paths (>5 kbp rearranged content) plus cyclic paths exceeds 0.3 of total *W* or (2) the ratio of weight in nonrearranged noncyclic paths exceeds 0.25 with more than four discordant SV edges in the amplified region. Otherwise, a linear amplification label is assigned. If no significant amplification remains after filtering, a ‘no amp/invalid’ label is assigned.

Classification rules and parameters were developed through iterative refinement on the basis of (1) empirical observations from metaphase-FISH validated samples and (2) theoretical understanding of focal amplification mechanisms. The 19 configurable parameters are provided as a JSON configuration file (default_config.json) in the AC codebase, with defaults established through parameter optimization against our FISH-validated benchmark and manual curation of predictions on CCLE, PCAWG and TCGA. Complete parameter documentation is available at https://github.com/AmpliconSuite/AmpliconClassifier/blob/main/AC_config_parameters.md. In brief, parameters control minimum size thresholds (for example, a 5-kbp minimum cycle size), CN thresholds (for example, CN >4.5 for focal amplifications), SV filtering criteria (for example, a 25-kbp maximum distance between inversion end points to call a foldback) and proportional thresholds for specific classifications (for example, 0.6 minimum CN-weighted path length supporting BFB). Parameter choices were evaluated by sweeps (Supplementary Table 4).

Complete resolution of focal amplification genome structure is often not possible from AA outputs alone owing to structural complexity, heterogeneity or the limitations of paired-end short-read WGS for CN and SV analysis. However, the structural signatures of focal amplification events remain detectable, allowing boundary resolution. Intervals are extracted from AC-marked genome paths and cycles containing segments above the default CN threshold (4.5). To refine boundaries beyond AA’s broader amplicon regions, AC distinguishes ecDNA and BFB regions from other amplified segments by identifying which high-CN regions decompose into ecDNA- or BFB-matching cycles. Neighboring amplified regions that do not are labeled ‘unknown’. If no ecDNA or BFB is detected, all amplified regions are classified as either linear or complex noncyclic.

AC annotates genes and lncRNAs found on the focally amplified intervals using the UCSC RefGene^40^ list packaged in the AA data repo, with oncogene annotations derived from COSMIC^41^ and ONGene^42^ databases, and GENCODE^43^ for lncRNAs.

We introduced methods to remove false-positive focal amplification calls from collections of unrelated samples, on the basis of the assumption that structurally identical focal amplifications across unrelated samples are highly improbable unless they are reference genome artifacts. The filter computes similarity scores for all genomically overlapping focal amplifications across the user-provided batch. Using the amplicon similarity *P* value described previously^10^, AC applies a Bonferroni-like correction with threshold $\frac{\alpha }{n-1}$, where *α* is the *P* value significance threshold (default 0.05) and *n* is the sample count (minimum 2). Features with $P < \frac{\alpha }{n-1}$ are filtered. Users enable this filtering in AC by setting --filter_similar.

### Parameter sweeps and downsampling comparison

To evaluate AC performance across experimental conditions, we conducted multiple in silico experiments. First, parameter robustness was assessed across AC’s 19 configurable parameters using our cytogenetically validated dataset. Supplementary Table 4 presents F1 scores (harmonic mean of precision and recall) for 12 parameters; the remaining 7 primarily modulate classification only in combination with other parameters and showed minimal independent effects. To evaluate downsampling effects, we analyzed 60 CCLE samples with baseline coverage >30× and at least one focal amplification (half ecDNA-positive at 10×), comparing AmpliconSuite-pipeline results at 10× versus 30× downsampling (--downsample 30).

### In silico dilution experiment

To evaluate ecDNA detection sensitivity across tumor purity levels, we performed in silico dilution experiments using nine high-coverage CCLE cancer cell lines (Supplementary Table 7) containing 34 distinct ecDNA species. For each cell line, original FASTQs were aligned to GRCh38, and aligned reads were mixed with reads from the normal diploid sample NA12878 (GIAB)^44^ to create 28 tumor purity levels between 4.8% and 95.2%. Read mixing was performed by extracting random subsets of reads from each BAM using AA’s downsample.py, combining them proportionally to the desired purity and resorting the merged BAMs. Mixed samples were downsampled to ≥10× median coverage by AmpliconSuite-pipeline and analyzed using AC v1.5.1 with default parameters. A baseline ecDNA was considered successfully re-identified if its genomic coordinates overlapped any ecDNA detected in the diluted sample.

We computed a theoretical detection limit to assess whether AC recovered ecDNAs having sufficient CN for expected detection. For a tumor with baseline ploidy *c*, purity *p* and ecDNA CN *e*, the measured CN *b*, is

$$b=\left(1-p\right)c+p\left(c+e\right)=c+{pe}.$$

A focal amplification must achieve a measured CN threshold, *t*, for focal amplification detection, such that *b* ≥ *t*. By default, *t* = *c* + 2.5. This implies *pe* ≥2.5 is required for detection of the ecDNA. This theoretical detection limit curve is shown in Extended Data Fig. 5.

### Analysis of ecDNA context

We developed ecContext, a hierarchical decision tree for ecDNA contextualization, to characterize the frequency of ecDNA origins, particularly chromothriptic versus nonchromothriptic (Extended Data Fig. 7a). These predictions are a default AC output. The method takes three inputs:

1. The AA breakpoint graph file, specifying the SV breakpoint edge locations and the CNs of genomic segments in the amplicon.
2. The AA cycles file, specifying a list of cyclic and noncyclic paths in the breakpoint graph, along with the CN explained by the path.
3. The ecDNA intervals file reported by AC listing the genomic segments within an AA amplicon, which form an ecDNA.

ecDNA intervals are first expanded by 10 kb on both ends to capture the surrounding context. ecContext then computes six scores on the basis of segments and breakpoint edges from the graph file lying completely within (not merely overlapping) these intervals. To identify ecDNA-relevant paths, ecContext filters out paths in the AA cycles file that use non-ecDNA SV edges. The remaining segments, SV edges and paths are used to compute the following classification scores:

(i) Cycle fraction (*C*): this represents the fraction of the length of the ecDNA regions weighted by CN that can be explained by the heaviest cyclic path reported by AA. First, for each cyclic path comprised solely of genome segments found on the ecDNA, with length *S*_P_ and CN assigned to that path *C*_P_, we calculate its weight as
  $${W}_{{\rm{P}}}={S}_{{\rm{P}}}\times {C}_{{\rm{P}}}.$$
  We next define the CN-weighted length of the regions, representing the denominator of the cycle fraction. Denoting the sizes of the ecDNA genome segments as ${s}_{1},{s}_{2},{s}_{3}\ldots$ and corresponding CNs ${c}_{1},{c}_{2},{c}_{3}\ldots$, the CN-weighted length of ecDNA regions, *W*_e_, is calculated as
  $${W}_{{\rm{e}}}=\sum _{i}{s}_{i}\times {c}_{i}.$$
  ecContext computes this quantity for each cyclic path comprised solely by ecDNA intervals. For the path *P* with the largest weight, we calculate the cycle fraction as
  $$C=\frac{{W}_{{\rm{P}}}}{{W}_{{\rm{e}}}}$$
(ii) Number of transitions (*T*): a transition is a CN change between two genomically adjacent segments in the AA graph. To count transitions, ecContext first filters small (<5 kb) insertions/deletions and joins neighboring segments flanking the filtered indel if their CNs differ by less than 2. Transitions are then counted from this updated segment list: each pair of consecutive segments increments the count by 1 if either (1) they are >5 kb apart in the reference or (2) both are ≥5 kb in size and differ in CN by ≥2.
(iii) Copy number states (*N*): using the merged segment list from above, ecContext deduces the total number of ecDNA CN states. Segment CNs are sorted in ascending order, and ecContext iterates through them, maintaining a running group average. Once a CN deviates from the current group average by a threshold (default 2), the rounded average of the previous group is recorded as a state and a new group starts. The final number of groups is the number of CN states.
(iv) Number of cross edges (cross SVs) (*E*): two breakpoint edges are crossing if one breakpoint end falls between the two ends of another edge on the same chromosome. Interchromosomal edges are also counted as cross edges. Each crossing pair increments *E* by 1.
(v) Number of foldbacks (*F*): inverted duplications give rise to a special SV class called a ‘foldback’. A read pair is consistent with a foldback SV if both mates have the same orientation (inversion-like) and end points map ≤50 kbp apart on the reference. The ‘foldback fraction’ is the number of foldback SV edges divided by total ecDNA SV edges.
(vi) Unichromosomal or multi-chromosomal: we defined a unichromosomal ecDNA as having segments from only one chromosome or having only very small segments (each segment size <10 kbp) from other chromosomes, and a multi-chromosomal ecDNA as comprising significantly large (≥10 kbp) segments from multiple chromosomes.

Using the six scores above, ecContext classifies ecDNA into one of the following contexts. AA typically decomposes focal CN gains encapsulated by everted (head-to-tail) SV edges into circular paths. When the heaviest circular cycle is enclosed by a single everted (duplication-like) edge that probably represents the founding excision event, structural rearrangements within may reflect postformation evolution rather than the formation mechanism. ecContext therefore excludes genome segments inside these cycles from structural complexity calculations. Context classes are evaluated in the following order, with classification returned once all criteria for a class are met:

1. BFB-like: ecDNA may form from a BFB chromosome^26,27^ (Extended Data Fig. 7d). ecContext predicts that the ecDNA is likely to have formed after a BFB event by looking at the number of foldbacks in the ecDNA. If there are at least four foldbacks, the fraction of foldbacks among all edges in the ecDNA is >0.1, the ecDNA is unichromosomal and has at least three CN states, then ecContext classifies the origin as ‘BFB-like’. The CN states must be varied, as the CN profile produced by BFB will be nonuniform in the segments that underwent inverted duplication.
2. Two-foldback: ecDNA can form owing to regression of a replication fork, following by inappropriate annealing or recombination of the newly replicated strands^28,29^ (Extended Data Fig. 7e). This may result in a hairpin-capped double-stranded DNA fragment that is ejected from the fork. Replication of this capped DNA fragment creates a double-stranded circular ecDNA flanked by two foldback inversions. ecContext detects this signature by looking for foldbacks close to (<50 kb) the ends of the ecDNA. If a foldback is found near both ends, and the remaining scores point to a relatively simple structure—few cross edges (up to three), low number of transitions compared with the number of copy states^45^ $(\frac{T}{N} < 4)$, and few CN states (up to six)—ecContext classifies the ecDNA origin as ‘two-foldback’.
3. Heavily rearranged: chromothripsis gives rise to many ecDNA^24,25^ (Extended Data Fig. 7c), and we created the heavily rearranged class to capture ecDNA enriched in a chromothriptic origin. ecContext considers a region to be heavily rearranged if the number of CN states is high (>6), or the number of cross edges is high (>4) or the number of CN transitions is high relative to the number of CN states $(\frac{T}{N}\ge 4)$, such as would be found in a chromothriptic region with oscillating CN^45^. If there is only a single chromosome involved or if the contribution from other chromosomes is minor (<30 kb), ecContext classifies the origin as HRUC. Otherwise, the origin is called HRMC.
4. Simple circular: ecDNA also form from an excisional model mediated by localized double-strand breaks with or without a replication fork being involved, yielding an ecDNA closed by a head-to-tail junction^22,23^ (Extended Data Fig. 7b). ecContext checks multiple criteria, described in detail below, to determine if the ecDNA is consistent with a simple circular class, and secondly whether the simple circular ecDNA lies in a genomic background with many genomic rearrangements (complex background) or few genomic rearrangements (simple background).
  First, if an ecDNA cycle constituted solely by regions from a single chromosome cannot be identified in the AA cycles file, then simple circular is ruled out (moving the classification to category 5: unknown). At this point, owing to also ruling out the heavily rearranged class, the remaining ecDNA must have at most three cross edges, *T*/*N* ratio <4 and at most six CN states. Using the cycle fraction score (defined in bullet i), ecContext checks if either the heaviest cycle explains ≥15% of the total ecDNA region(s) CN or at least 30% of the CN is explained by a single trivial ecDNA cycle. If met, then a simple circular label can be applied.
  For ecDNA classified as simple circular, ecContext determines whether they occur in a simple or complex genomic background by assessing scores ii–iv from above on the entire amplicon, excepting regions inside simple everted-edge cycles. If the amplicon-wide scores indicate a *T*/*N* ratio ≥4, or four or more cross edges, or more than six CN states, the ecDNA is classified as SCCB, and SCSB otherwise.
5. Unknown: if no criteria are met, ecContext classifies the ecDNA as unknown. This category may include unrecognized ecDNA formation modes or highly specific mechanisms not captured by broader categories (for example, fragments derived from double-strand breaks during a chromosomal translocation event could form a multi-chromosomal ecDNA with otherwise simple circular properties).

We grouped two-foldback and SCSB labels as simple excision-like, and BFB-like, SCCB, HRUC and HRMC as chromothripsis-associated. An important caveat is that chromothripsis-associated classifications may capture simple ecDNA that has undergone extensive postformation rearrangement or recombination.

### Data repo construction

The AA data repos contain reference genome FASTAs and annotation files used by AA. The hg19 and GRCh37 data repos were previously constructed as described by Deshpande et al.^7^. To construct data repos for GRCh38 (hg38) and mm10 (GRCm38), first we obtained the required reference genome sequences and genome annotation files from publicly available sources. Broadly, the genome annotation files required in each AA data repo include a BED file of the centromeric coordinates, an excludable regions BED file containing poorly mappable and high-signal regions, a bedgraph file of genome-wide 35-bp mappability scores, a GFF file of genes, a GFF file of oncogenes, a BED file of regions with conserved CN gains in normal samples and the reference’s UCSC ‘SuperDup’ segmental duplication annotation file. Last, a merged and sorted BED file that combines the excludable regions, conserved gain regions and centromere regions is required by AmpliconSuite-pipeline, named ‘[genome]_merged_centromeres_conserved_sorted.bed’. While most annotations were available online, we generated 35-bp mappability and conserved gain files as described below.

To create the genome-wide 35-bp mappability scores, we used the ‘gem-mappability’ tool (version 1.315) from the GEnomic Multi-tool (GEM) library^46^ to compute genome-wide mappability of 35-bp sequences throughout the genome, giving as input the reference FASTA with all nonchromosomal and non-mtDNA sequences removed.

To identify conserved high CN gains found in normal genomes, we used collections of noncancer genomes. For GRCh38, we used WGS from seven normal samples published by Turner et al.^1^ as well as WGS from HG003, HG006 and NA12878 released by Genome in a Bottle (GIAB)^44^. For mm10, we used 40 mouse WGS samples from the Mouse Genomes Project^47^. We then used CNVkit^38^ (version 0.9.7) to create a flat reference against which CNVkit called CNV profiles. We added regions which showed CN >4.9 in at least 20% of the analyzed samples to the conserved gain region file. For mm10, oncogenic regions were subtracted from the list of conserved gain regions to improve detection of mouse ecDNA covering oncogenes.

To construct the GRCh38_viral data repo, we added additional oncoviral reference genome sequences into the reference genome FASTA file for GRCh38, leaving other files the same. Broadly, the oncoviral sequences are the papilloma and hepatitis viral sequences available from PaVE^48^ (https://pave.niaid.nih.gov) as well as from Jiang et al.^49^. The complete list of sequences is presented in Supplementary Table 1 by Nguyen et al.^50^.

### Cell lines

Cell lines used to generate metaphase-FISH images (BT-474, CCF-STTG1, CHP-212, COLO320DM, DLD1, DMS114, DMS273, H520, H524, H716, HCC1569, HCC1954, HCC38, HGC27, KATOIII, NCI-H1792, NCI-H2170, HCI-H69, OE-19, OVCAR-3, OVCAR-8, RT-112, SCLC-21H, SJSA-1, SK-BR-3, TR14, UACC1598 and UACC812) were maintained at 37 °C with 5% CO_2_ (sources and culture conditions presented in Supplementary Table 2). Genomic DNA was extracted using the QIAamp DNA Kit (Qiagen) per the manufacturer’s protocol. Libraries were sequenced on a NovaSeq X Plus platform (Novogene) generating two 150-bp paired-end reads at approximately 10× coverage.

### Analysis of sequencing data

Cancer Cell Line Encyclopedia (CCLE) WGS data were obtained from SRA (PRJNA523380), and the FASTQ files were processed by AmpliconSuite-pipeline (v0.1032.4), using BWA^51^ (0.7.17-r1188), CNVkit^38^ (0.9.7) and AA^7^ (v1.3.r1), and classified with AC^10,39^ (v1.5.1). Cell line WGS fastqs used in validation experiments were analyzed with AmpliconSuite-pipeline (v1.3.6) using BWA (0.7.17-r1188), CNVkit (0.9.7) and AA (v1.5.r1), and classified with AC (v1.5.1).

### Metaphase FISH

About 2 million cells were plated into 6-cm dishes and cultured for 24 h. The next day, metaphase cells were enriched by a 4-h or overnight KaryoMAX colcemid treatment at a final concentration of 0.1 μg ml^−1^ (Gibco, 15210040). Cells were trypsinized and pelleted at 400*g* for 4 min along with cells collected in the supernatant. The cell pellet was resuspended by pipetting in 400 μl 0.75 M KCl and incubated at 37 °C for 20 min to allow hypotonic swelling of cells. An equivalent amount of freshly prepared Carnoy’s fixative (3:1 methanol:glacial acetic acid) was added to the cells and mixed by thorough pipetting, and they were spun down at 2,000*g* for 3 min. Two additional washes with Carnoy’s fixative was performed, and the cells were resuspended in ~150 μl Carnoy’s fixative. To prepare metaphase spreads, 13 μl of cell suspension was dropped onto humidified frosted glass slides and allowed to air-dry. The slides were subjected to a brief rinse at 2× SSC, followed by dehydration in ascending ethanol concentrations (70%, 85% and 100%) for 2 min each. DNA FISH probes (Empire Genomics, probe information presented in Supplementary Table 2) were diluted with hybridization buffer (0.6 μl probe in 7 μl buffer), added to the sample and concealed with a coverslip. The slides were subjected to heat denaturation at 75 °C for 3 min and incubated overnight in a humidified slide moat at 37 °C for hybridization. The next day, coverslips were removed, washed in 0.4× SSC and then in 2× SSC supplemented with 0.1% Tween-20 for 2 min each. DNA was counterstained with 4,6-diamidino-2-phenylindole (DAPI) at 50 ng ml^−1^ for 10 min at room temperature and briefly rinsed in ddH_2_O before being air-dried. Samples were mounted with ProLong diamond antifade mountant (Invitrogen, P36961). Images were acquired on a Leica DMi8 widefield microscope using a 63× objective and the LAS X v3.9.1 software. Typically, ten or more images were collected per probe.

### FISH image labeling

Metaphase FISH images were independently reviewed and labeled by two ecDNA FISH interpretation experts (I.T.-L.W. and J.L.). Each gene was assigned one of the following categories on the basis of the observed signal patterns: ecDNA, native HSR, non-native HSR or unknown HSR. A gene was labeled ecDNA if at least two images showed unambiguous ecDNA signal (distinct, punctate extrachromosomal foci not attributable to imaging artifacts). Native/non-native HSR classifications required a centromere probe for the gene’s chromosome of origin: genes were labeled non-native HSR if the majority (>50%) of images showed at least one HSR signal on a chromosome other than the gene’s chromosome of origin, and native HSR otherwise. In cases of disagreement between the two raters, a consensus label was reached through discussion. Cases that showed non-native HSR but whose WGS data suggested ecDNA were marked neutral in performance calculations of AC (24 probed genes), as were cases with indeterminate status for ecDNA (3 probed genes), or for which AC marked the region as ‘unknown’—indicating an indeterminate focal amplification type in WGS data (6 probed genes). Probed genes which resolved as HSR but did not include a centromere probe were labeled unknown HSR.

### AmpliconRepository framework

The core of AmpliconRepository is built as a Django-based^52^ web application. User authentication is done using Globus Auth^53^, which supports OAuth2^54^ and OPenID Connect^55^, allowing single sign-on from many educational institutions and commercial identity providers (for example, Google). Project and sample metadata are stored in a Mongo-compatible document database, while AA output files and images are stored on the server’s local filesystem or in an Amazon Web Services (AWS) S3 bucket for scalability. Interactive sample plots are built using Plotly^56^ for CN and focal amplification abundances, and igv.js^57^ for region-level gene annotation plots. The AmpliconRepository server runs from a Docker container on an AWS EC2 instance, using S3 for files and AWS DocumentDB as the primary database. Private AWS-free instances can also be deployed using Docker or Singularity with MongoDB on a local filesystem where privacy or data confidentiality is required.

AmpliconSuiteAggregator aggregates a collection of AmpliconSuite-pipeline outputs into a single compressed file. Users provide a zip or tarball of unstructured output files (CNV seed calls, AA and AC outputs), which the tool decompresses and groups by sample name. Files are moved into a predetermined directory structure with a generated JSON index containing file paths for each sample, and the resulting directory is compressed. AmpliconSuiteAggregator runs in the backend when a user uploads a project.

Project uploads can be performed through the AmpliconRepository project creation page or by specifying a project’s UUID and secret key to AmpliconSuite-pipeline with the -upload argument, which triggers a POST via curl.

### AmpliconRepository co-amplification graph utility

To enable systematic analysis of gene co-amplification patterns mediated by ecDNA, we developed a Co-amplification Graph utility integrated into AmpliconRepository. Users select one or more projects sharing the same reference genome build and then click ‘generate co-amplification graph’ to initiate processing.

The utility constructs a Neo4j graph database where nodes represent genes and edges represent co-amplification relationships. Gene locations are extracted from reference annotation BED files (hg19 or GRCh38). Genomic intervals from each ecDNA are first merged if their end points are within 300 kb (merge cutoff parameter), representing the maximum deletion size permitted considering a gene pair to originate from a single interval. Genes are included only if amplified on ecDNA in at least three samples (minimum gene occurrence threshold).

Nodes are annotated with samples amplifying that gene; edges are annotated with co-amplifying samples and the breakdown of co-amplification type. Co-amplifications are categorized into four mutually exclusive types per sample by analyzing the merged genomic intervals of each gene pair: (1) single interval—genes on the same chromosome and ecDNA within the same merged interval; (2) multi-interval—genes on the same chromosome and ecDNA but on different intervals (>300 kb apart); (3) multi-chromosomal—genes from different chromosomes joined on a single ecDNA; and (4) multi-ecDNA—genes amplified on distinct ecDNA within the same sample. Edge weights reflect the number of samples exhibiting each relationship.

The resulting network is visualized using Cytoscape.js^58^, providing an interactive interface where users can search for genes, adjust visualization parameters (edge count thresholds and number of nodes displayed) and download results in tabular format with gene pairs as rows and co-amplification type counts as columns.

### Statistical analysis

The relevant statistical tests are named in the ‘Results’ section and in the figure legends. Statistical tests were performed with SciPy^59^ version 1.13.0. All statistical tests were two-sided unless otherwise stated.

### Reporting summary

Further information on research design is available in the Nature Portfolio Reporting Summary linked to this article.

## Online content

Any methods, additional references, Nature Portfolio reporting summaries, source data, extended data, supplementary information, acknowledgements, peer review information; details of author contributions and competing interests; and statements of data and code availability are available at https://doi.org/10.1038/s41592-026-03264-x.

## Supplementary information

Reporting Summary

Peer Review File

Supplementary Table 1 Supplementary Table 1.

Supplementary Table 2 Supplementary Table 2.

Supplementary Table 3 Supplementary Table 3.

Supplementary Table 4 Supplementary Table 4.

Supplementary Table 5 Supplementary Table 5.

Supplementary Table 6 Supplementary Table 6.

Supplementary Table 7 Supplementary Table 7.

Supplementary Table 8 Supplementary Table 8.

## Source data

Source Data Figs. 1 and 2 and Extended Data Fig. 9 Source Data Fig. 1e. Source Data Fig. 2c Source Data for Fig. 2c. Source Data Fig. 2d Source Data for Fig. 2d. Source Data Fig. 2e Source Data for Fig. 2e. Source Data Fig. 2i Source Data for Fig. 2i. Source Data Fig. 2j Source Data for Fig. 2j. Source Data Extended Data Fig. 9 Source Data for Extended Data Fig. 9.

## Data Availability

WGS data for CCLE were downloaded from SRA (PRJNA523380). New sequencing data produced for cell lines used in validation analysis are available through SRA (PRJNA1464264), and the associated metaphase FISH imaging is available via the Stanford Digital Repository at https://doi.org/10.25740/xs547km2145 (ref. ^60^). AmpliconSuite-pipeline outputs for samples used in metaphase FISH validation are available via AmpliconRepository at https://ampliconrepository.org/project/68d06aa57116a5e41cb99d38 and https://ampliconrepository.org/project/68d06e477116a5e41cb9cc74. ICGC (PCAWG + TCGA), HMF and GLASS AA outputs were previously published^20^ and downloaded from AmpliconRepository at https://ampliconrepository.org/project/6a5a7edc6b83a17ac958761e, https://ampliconrepository.org/project/6a5a4f41f0fb4735a8586742, https://ampliconrepository.org/project/6a5a946971bfe99e4bd6e7b0 and https://ampliconrepository.org/project/6a5a559851b225cd74586742. Optical genome mapping data used in comparisons with OM2BFB were previously published^15^. Source data are provided with this paper.

## Code availability

All code developed for this study is available via GitHub at https://github.com/AmpliconSuite.
