# Supplementary material for: AmpliconSuite: an end-to-end workflow for analyzing focal amplifications in cancer genomes
Source: Nat Methods. 2026 Oct 16;23(11):2141–6. doi: 10.1038/s41592-026-03264-x (PMC13736199; doi:10.1038/s41592-026-03264-x)
Supplement: Supplementary file 1 [file 41592_2026_3264_MOESM1_ESM.pdf]

nature portfolio

Corresponding author(s): Vineet Bafna (vbafna@ucsd.edu), Jens Luebeck (jluebeck@ucsd.edu)

Last updated by author(s): Jun 15, 2026

Reporting Summary

Nature Portfolio wishes to improve the reproducibility of the work that we publish. This form provides structure for consistency and transparency in reporting. For further information on Nature Portfolio policies, see our Editorial Policies and the Editorial Policy Checklist.

Statistics

For all statistical analyses, confirm that the following items are present in the figure legend, table legend, main text, or Methods section.

n/a	Confirmed	
☐	☒	The exact sample size (*n*) for each experimental group/condition, given as a discrete number and unit of measurement
☐	☒	A statement on whether measurements were taken from distinct samples or whether the same sample was measured repeatedly
☐	☒	The statistical test(s) used AND whether they are one- or two-sided<br>*Only common tests should be described solely by name; describe more complex techniques in the Methods section.*
☒	☐	A description of all covariates tested
☒	☐	A description of any assumptions or corrections, such as tests of normality and adjustment for multiple comparisons
☐	☒	A full description of the statistical parameters including central tendency (e.g. means) or other basic estimates (e.g. regression coefficient) AND variation (e.g. standard deviation) or associated estimates of uncertainty (e.g. confidence intervals)
☐	☒	For null hypothesis testing, the test statistic (e.g. *F*, *t*, *r*) with confidence intervals, effect sizes, degrees of freedom and *P* value noted<br>*Give P values as exact values whenever suitable.*
☒	☐	For Bayesian analysis, information on the choice of priors and Markov chain Monte Carlo settings
☒	☐	For hierarchical and complex designs, identification of the appropriate level for tests and full reporting of outcomes
☒	☐	Estimates of effect sizes (e.g. Cohen's *d*, Pearson's *r*), indicating how they were calculated

Our web collection on statistics for biologists contains articles on many of the points above.

Software and code

Policy information about availability of computer code

Data collection

CCLE WGS data was downloaded using SRA-toolkit: https://github.com/ncbi/sra-tools/releases/tag/3.0.0.

Data analysis

BWA (version 0.7.17-r1188)
Samtools (version 1.10-3)
CNVKit (version 0.9.7)
AmpliconArchitect (versions 1.3.r1, 1.3.r2, 1.5.r2)
AmpliconClassifier (version 2020, 0.4.13, 1.5.1)
AmpliconSuite-pipeline (versions 0.1032.4, 0.1344.2, 1.3.7)
Scipy (version 1.13.0)
GEnomic Multi-tool (GEM) library (version 1.315)
LAS X (version 3.9.1)

The AmpliconSuite github organization contains custom code created for this project: https://github.com/AmpliconSuite

For manuscripts utilizing custom algorithms or software that are central to the research but not yet described in published literature, software must be made available to editors and reviewers. We strongly encourage code deposition in a community repository (e.g. GitHub). See the Nature Portfolio guidelines for submitting code & software for further information.

Data

Policy information about availability of data

All manuscripts must include a data availability statement. This statement should provide the following information, where applicable:

- Accession codes, unique identifiers, or web links for publicly available datasets
- A description of any restrictions on data availability
- For clinical datasets or third party data, please ensure that the statement adheres to our policy

New sequencing data produced for cell lines used in validation analysis is available through SRA (PRJNA1464264) and the associated metaphase FISH image is available on the on Stanford Digital Repository (https://doi.org/10.25740/xs547km2145). AmpliconSuite-pipeline outputs for samples used in metaphase FISH validation are available from AmpliconRepository.org (https://ampliconrepository.org/project/68d06aa57116a5e41cb99d38, https://ampliconrepository.org/project/68d06e477116a5e41cb9cc74). WGS data for CCLE was downloaded from SRA (PRJNA523380), ICGC (PCAWG + TCGA), HMF, and GLASS AA outputs were previously published and downloaded from AmpliconRepository.org: ampliconrepository.org/project/CCLE, ampliconrepository.org/project/PCAWG, ampliconrepository.org/project/TCGA, ampliconrepository.org/project/HMF, ampliconrepository.org/project/GLASS . The original sequencing data for PCAWG and TCGA data was downloaded from ICGC (DACO application # DACO-753) and TCGA data was obtained from dbGaP (phs000178.v11.p8). Optical genome mapping data utilized in comparisons with OM2BFB was previously published (https://pubmed.ncbi.nlm.nih.gov/39402156/). A list of IDs of additional previously published analyzed WGS samples is available in Supplementary Table 1.

Research involving human participants, their data, or biological material

Policy information about studies with human participants or human data. See also policy information about sex, gender (identity/presentation), and sexual orientation and race, ethnicity and racism.

Reporting on sex and gender	Our study is a meta-analysis of existing large-scale cancer datasets (CCLE, PCAWG, TCGA, HMF, GLASS). Information about the sex of tissue donors is available in the parent studies. The method for copy number detection we use to select regions to study with AA automatically determines the non-autosomal chromosome count of the individual. Sex information for cell lines used here is available through the SRA metadata here: PRJNA1464264
Reporting on race, ethnicity, or other socially relevant groupings	N/A
Population characteristics	This study combined analyses of commerically available cancer cell lines (metadata available at PRJNA1464264), and publicly available and access-controlled large-scale cancer sample cohorts representing samples from 6,506 tumor samples from CCLE, PCAWG, TCGA, HMF, and GLASS. The population characteristics of each donor are addressed in the parent studies.
Recruitment	Our study is a meta-analysis of existing large-scale cancer datasets (CCLE, PCAWG, TCGA, HMF, GLASS), and no human subjects were recruited specifically for our study. Information about human subject recuitment is available in the parent studies.
Ethics oversight	Our study is a meta-analysis of existing large-scale cancer datasets (CCLE, PCAWG, TCGA, HMF, GLASS) and commerically available cell lines. Information about ethics oversight of those datasets is available in the parent studies.

Note that full information on the approval of the study protocol must also be provided in the manuscript.

Field-specific reporting

Please select the one below that is the best fit for your research. If you are not sure, read the appropriate sections before making your selection.

☒ Life sciences ☐ Behavioural & social sciences ☐ Ecological, evolutionary & environmental sciences

For a reference copy of the document with all sections, see nature.com/documents/nr-reporting-summary-flat.pdf

Life sciences study design

All studies must disclose on these points even when the disclosure is negative.

Sample size	This meta-analysis of whole genome sequencing data from patient samples did not utilize a sample size calculation. Sample size was determined by the 6,506 samples considered in this study from PCAWG, TCGA, HMF, GLASS, the broadest available cohort. This number of samples was sufficient for statistical analysis. A collection of 401 cell lines was also considered, which was considered sufficient for statistical analysis. For FISH imaging, approximately 10 images were gathered per probe, which was deemed sufficient to infer cytogenetic status of the probed gene.
Data exclusions	Samples below a tissue purity of 0.4 were excluded from TCGA, PCAWG, HMF, GLASS.
Replication	Individual tumor sample biospecimens from patients cannot be precisely replicated as tumors contain spatial genomic heterogeneity. Our study provides a descriptive summary of existing large-scale cancer datasets. For FISH experiments, multiple images were gathered per probe - typically around 10.

Randomization	No randomization was performed given the descriptive nature of our study.
Blinding	No blinding was performed given the descriptive nature of our study.

Reporting for specific materials, systems and methods

We require information from authors about some types of materials, experimental systems and methods used in many studies. Here, indicate whether each material, system or method listed is relevant to your study. If you are not sure if a list item applies to your research, read the appropriate section before selecting a response.

Materials & experimental systems

n/a	Involved in the study
☒	☐ Antibodies
☐	☒ Eukaryotic cell lines
☒	☐ Palaeontology and archaeology
☒	☐ Animals and other organisms
☒	☐ Clinical data
☒	☐ Dual use research of concern
☒	☐ Plants

Methods

n/a	Involved in the study
☒	☐ ChIP-seq
☒	☐ Flow cytometry
☒	☐ MRI-based neuroimaging

Eukaryotic cell lines

Policy information about cell lines and Sex and Gender in Research

Cell line source(s)	The source of cell lines used is present in Supplementary Table 2, and the sex of cell lines used is present in the SRA metadata: PRJNA1464264
Authentication	Cell lines were not authenticated as they were purchased from the vendors listed in Supplementary Table 2
Mycoplasma contamination	All cell lines tested negative for mycoplasma contamination.
Commonly misidentified lines (See ICLAC register)	None

Plants

Seed stocks	N/A
Novel plant genotypes	N/A
Authentication	N/A
