# Supplementary material for: AmpliconSuite: an end-to-end workflow for analyzing focal amplifications in cancer genomes
Source: Nat Methods. 2026 Oct 16;23(11):2141–6. doi: 10.1038/s41592-026-03264-x (PMC13736199; doi:10.1038/s41592-026-03264-x)
Supplement: Supplementary file 2 [file 41592_2026_3264_MOESM2_ESM.pdf]

nature portfolio

Peer Review File

AmpliconSuite: an end-to-end workflow for analyzing focal amplifications in cancer genomes

Corresponding Author: Professor Vineet Bafna

This file contains all editorial decision letters in order by version, followed by all author rebuttals in order by version.

A version of this paper was originally rejected for publication by Nature Methods, however that decision was reconsidered after appeal by the authors.

Version 0:

Decision Letter:

18th Feb 2024

Dear Dr Bafna,

Thank you for submitting your manuscript entitled "AmpliconSuite: an end-to-end workflow for analyzing focal amplifications in cancer genomes". Please accept our sincere apologies for the length of time that it has taken to get the decision on your manuscript due to the Spring Festival holiday resulting in a backlog of new manuscripts.

I’m happy to say that we plan to send your manuscript out for peer review, though we think that Brief Communication would be the most appropriate format. Please see our editorial about the content type offering at Nature Methods.
https://www.nature.com/articles/s41592-020-0927-4

To fit into the BC format, we suggest you shorten the manuscript now or after peer review. We will leave it to your discretion. Moreover, we ask you to provide the related manuscripts mentioned in your submission form. We will need to check the overlapping contents among these manuscripts.

We shall hope to receive your resubmission as soon as possible. If you anticipate a delay of more than two weeks, please let us know. In this event, we will still be happy to reconsider your paper at a later date so long as nothing similar has been accepted for publication at Nature Methods or published elsewhere.

Nature Methods is committed to improving transparency in authorship. As part of our efforts in this direction, we are now requesting that all authors identified as ‘corresponding author’ on published papers create and link their Open Researcher and Contributor Identifier (ORCID) with their account on the Manuscript Tracking System (MTS), prior to acceptance. This applies to primary research papers only. ORCID helps the scientific community achieve unambiguous attribution of all scholarly contributions. You can create and link your ORCID from the home page of the MTS by clicking on ‘Modify my Springer Nature account’. For more information please visit please visit www.springernature.com/orcid.

If you are not interested in resubmitting the manuscript please let me know immediately so we can close your file. If you have any questions, please contact me.

Please use the link below when you are prepared to resubmit.
Link Redacted

Note: The above URL links to your confidential home page and associated information about manuscripts you may have submitted, or that you are reviewing for us. If you wish to forward this email to co-authors, please delete the link to your homepage.

Thank you for your interest in Nature Methods.

Sincerely,
Lei

Lei Tang, Ph.D.
Senior Editor
Nature Methods

** For Nature Portfolio general information and news for authors, see http://npg.nature.com/authors.

Version 1:

Decision Letter:

7th Apr 2024

Dear Dr Bafna,

Your Brief Communication entitled "AmpliconSuite: an end-to-end workflow for analyzing focal amplifications in cancer genomes" has now been seen by 3 reviewers, whose comments are attached. While they find your work of potential interest, they have raised serious concerns which in our view are sufficiently important that they preclude publication of the work in Nature Methods, at least in its present form.

As you will see, the reviewers raise substantial concerns about the methodology novelty and the potential overlap with your previous publications.

Should further experimental data allow you to fully address these criticisms we would be willing to look at a revised manuscript (unless, of course, something similar has by then been accepted at Nature Methods or appeared elsewhere). This includes submission or publication of a portion of this work somewhere else. We hope you understand that until we have read the revised paper in its entirety we cannot promise that it will be sent back for peer-review.

If you are interested in revising this manuscript for submission to Nature Methods in the future, please contact me to discuss your appeal before making any revisions. Otherwise, we hope that you find the reviewers' comments helpful when preparing your paper for submission elsewhere.

Sincerely,
Lei

Lei Tang, Ph.D.
Senior Editor
Nature Methods

Reviewers' Comments:

Reviewer #1:
Remarks to the Author:
The authors describe the AmpliconSuite package that they developed; this builds on their earlier published AmpliconArchitect method and adds a classifying algorithm for the detected amplicons. AmpliconSuite is then applied to ~2500 WGS samples from cell line and patient data.

AmpliconClassifier and the methodology behind the classification have already been used by the authors in earlier publications (Kim et al., Nature Genetics, 2020 and Luebeck et al., Nature, 2023). Both these publications include the analysis of large datasets after classification using AmpliconClassifier.

As these publications show, a tool like AmpliconClassifier is very important when studying ecDNA-based cancers. For this manuscript to be a strong addition to the ecDNA literature, it should in figure 2 very clearly demonstrate how the current iteration of AmpliconClassifier outperforms the versions that were used in Kim et al. and Luebeck et al. In figure 3, there should be a similar comparison: which results are improved in upgrading to the current iteration of AmpliconClassifier?

In figure 2, the authors describe the performance of AmpliconArchitect and AmpliconClassifier. The performance improvement of AmpliconArchitect looks impressive. I am less convinced with the methodology behind AmpliconClassifier, for two reasons: first of all, the classification methodology relies on a number of filtering steps and neither the specific choices nor the parameter values are explained. Second of all, since this is a classifier the authors should describe their training and test dataset, how the training set is used to determine the parameter values, and how the performance is on the training and on the test set.

I have the following major suggestions for improvement:

- There should be a systematic approach behind determining the parameter values, and the performance of the classifier should be measured on a separate test dataset.
- In my understanding, an HSR that consists of amplicons that are linearly integrated in tandem would generate almost exactly the same signal as ecDNA. The authors should explain and simulate why this type of linear amplification is not classified as ecDNA.
- For figure 3, I think it would be important to show which of these results could have already been obtained by earlier iterations of AmpliconClassifier.
- Since AmpliconClassifier has already been published, I feel it would be important to show what novel biological insights can be gained by it. In that sense, I don't think Figure 3 does AmpliconClassifier/AmpliconSuite enough justice.

Minor comments:
- The first three paragraphs of the result section seem to be a description of AmpliconArchitect. I am sure that this can be condensed and made less technical.
- Many of the improvements made to AmpliconArchitect are not supported by data or figures.
- It is laudable that the authors have tried to make the installation of AmpliconSuite as flexible and simple as possible. However, I am not sure it needs to be described to this length in the main text. Similarly, I think the description of AmpliconRepository's user interface can be reduced.
- It would be great if the authors could add some reasoning behind the various filtering steps in AmpliconClassifier.
- Can the authors explore which WGS coverage levels are necessary for correct classification and whether results are consistent over various downsamplings of the WGS data?

Reviewer #2:
Remarks to the Author:
In their article "AmpliconSuite: an end-to-end workflow for analyzing focal amplifications in cancer genomes", Luebeck et al. describe a novel toolkit and analysis pipeline integrating previously published methods with novel approaches to infer amplicon structures from whole-genome sequencing in a user-friendly manner. They highlight improvements in their main method AmpliconArchitect, introduce a new method ecContext as part of their AmpliconClassifier method and demonstrate usability of their toolkit on a set of previously published cancer data. A community building website accompanies the package.

Focal amplifications are currently an important area of research in cancer genomics and as such the paper deals with a timely subject. We believe the toolkit has the potential to find wide application in the community, and we particularly applaud the authors for the community building website which will surely increase accessibility of the tool and its results. However, we do have substantial concerns regarding the manuscript in its current form, largely related to the novelty of the work and the justification of methodological approaches.

Major comments

1. The authors present improvements to the overall usability and performance of the previously published tools AmpliconArchitect and AmpliconClassifier. However, the main novel methodological contribution seems to be ecContext, a rule-based tool for classifying ecDNAs based on their genetic context. While certainly interesting, ecContext feels somewhat underdeveloped. Described in a short paragraph in the main text it employs various ad-hoc chosen parameters and cutoffs to classify ecDNAs. Unfortunately, we could not find a systematic evaluation of the performance of the tool, and no thorough explanation is given about how the arbitrary thresholds and parameters were selected.

2. Additionally, the relationship between the features computed by ecContext (enumerated as (i) to (vi) in the Methods section) and the resulting ecDNA classes (enumerated as 1. to 5.) is unclear. A tabular description or decision tree of all rules and how they are applied together with the precise thresholds and their motivation (see comment 1) should be given.

3. The authors report on improvements of detection of focal amplifications including ecDNA, but only give sensitivity values for ecDNA detection. Can the authors provide a thorough evaluation of the detection and classification performances of all parts of AmpliconSuite including in particular accuracy measures for the classification of other focal amplifications outside ecDNAs?

4. While ecDNAs are relatively easy to detect using FISH allowing generation of an experimental gold standard, this seems to be more difficult for other types of amplifications. The choice of a benchmark dataset needs to be discussed, the used cell lines were also used for the AmpliconArchitect paper and it is unclear how accurate these reference calls are. Are other novel datasets available to corroborate the findings from the cell lines?

5. As a side note, the Kim et al. paper states that 44 cell lines were used, the authors here use only 43 cell lines. What happened to that one cell line?

6. Beyond additional test datasets, sophisticated simulations could reveal the improved performance of AmpliconSuite over prior implementations and elucidate sensitivity, specificity and classification accuracy values more systematically.

7. The authors explored the complexity and genomic span of focal amplifications. They report lower complexity and higher variance in genomic span in ecDNA compared to BFB. Can the authors speculate on why this could be observed?

8. The authors state that biologically unrelated samples should not share exact genomic boundaries and implement a similarity score-based filter to control for false-positive focal amplifications. How might specific phenomena that predispose the genome

to break at specific positions (such as fragile sites) influence this assumption? Can the authors give an example how this filter improves the analysis and elaborate on when to use the filter?

Minor comments

l.275: Caner Cell Line → Cancer Cell Line
l.135: 'may arise due from' → 'may arise from' or 'may arise due to'
l.102: the abbreviation SV for structural variants is defined here, but structural variant appears earlier (l.59)
l.40,58,79: multiple definitions of the abbreviation AA for AmpliconArchitect; similar for AC, please check

Remarks on code availability:
We reviewed the code and found it well documented, but did not run it ourselves.

Reviewer #3:
Remarks to the Author:
Jens Luebeck and colleagues reported the development of an all-in-one bioinformatic pipeline AmpliconSuite for analyzing focal amplifications in cancer genomes. The AmpliconSuite pipeline developed by the study has made significant efforts to improve and integrate the analytic packages for analyzing, visualizing, and interpreting focal amplifications in pair-end WGS cancer genomes. Although many packages were integrated into the AmpliconSuite-pipeline with their original but well-developed codes, significant amounts of modifications and improvements have been introduced into the AA, AC, and ecContext.
The AmpliconSuite could not be regarded as a completely novel method. However, it is a significantly improved and streamlined tried-and-tested technique for focal amplification analysis. The research team has significantly improved the sensitivity and performance of the AmpliconArchitect (AA) and AC. As a validation of the codes developed by the study, we have performed back-to-back comparison of the older version of AA and the AmpliconSuite pipeline using in-house generated WGS data from UBC. In line with the sensitivity and performance reported by the study, the AmpliconSuite-pipeline is significantly faster, smoother, and more informative than previous AA. Since there are rapidly increasing interests in understanding the roles and translatability of ecDNAs in cancer diagnosis and treatment, the AmpliconSuite will become one of the most popular and broadly used tool kits for studying focal amplification and ecDNAs in cancer genomes.
Furthermore, this study has developed an interoperable database ampliconrepository for the scientific community to share, re-use, and analyze focal amplification data. Although the ampliconrepository will benefit from some further modifications (comments below), it is a great example of the foundational FAIR (Findability, Accessibility, Interoperability, and Reusability) principles.
To demonstrate the broad applicability of the AmpliconSuite-pipeline, the study has analyzed over 2500 WGS genomes from tumors and cancer cell lines. These analyzed data have been shared and illustrated in their database AmpliconRepository.org.
A conceptionally interesting and important point introduced by the study is ecContext for categorizing the ecDNAs by their generative mechanism. Although some of these categorizations remain discussing, this is an important step toward better understanding of the ecDNA functions.
Overall, the AmpliconSuite is a significant improvement to the tried-and-tested AmpliconArchitect bioinformatic tool. This is a timely all-in-one and end-to-end workflow to meet the great needs of analyzing cancer WGS data. The nature of code-free (GenePattern and nextflow) and end-to-end makes the method to be easily adapted by the broad scientific community, even with limited bioinformatic skills. Although the manuscript, methods, and figures have already been well prepared, the study and manuscript will benefit from addressing a few important points.
1. The AmpliconSuite-Pipeline
We have tested the AmpliconSuite pipeline and codes with in-house generated WGS data from human tumors. The performance of the pipeline is excellent in term of speed and operability. The amplicon detection results are consistent between the old and new version, but the contexts provided have been significantly improved in the new version as compared to the older one. However, it is unclear about the effect of WGS coverage on the AmpliconSuite (or AA) sensitivity. The previous AA has suggested 5-10X coverage. In the current study, only samples with at least 10X coverage and 0.4 tumor purity had been included for analysis. Although the authors have commented that the pipeline does not include the QC assessment of the WGS dataset, assigning certain degree of QC cutoffs will benefit the subsequent projects and results inclusion in the AmpliconRepository.
The analyzed results provided by AmpliconSuite are organized into three folders containing quite a large number of sub-folders and files. The readiness of the results will be significantly improved by including the code for generating an accompany html report as results output.
2. The AmpliconRepository portal
I have been exploring the AmpliconRepository database generated by this study. The interoperability of AmpliconRepository makes this database unique and distinct from the eccDNA Atlas database (https://lcbb.swjtu.edu.cn/eccDNAatlas/index.html). Both eccDNA Atlas and AmpliconRepository database mainly focus on providing static results on focal amplicon and eccDNA (including ecDNA).
One important value of studying focal amplification and ecDNA in cancer is related to their potential role in cancer prognosis. It will be relevant and highly useful to include the prognostic value of ecDNA genes, amplicon classification, and ecContext in the database.
The Human Protein Atlas (e.g. https://www.proteinatlas.org/ENSG00000146648-EGFR/pathology/urothelial+cancer) have provided excellent examples for interactively exploring the prognostic value of genes.
Particularly, since the study has already analyzed over 2000 tumor samples from PCAWG and TCGA, it should be feasible to explore the prognostic potential of the ecDNA genes and focal amplicons.
3. Classification of ecDNA and HSR with AA

ecDNA can subsequently integrate into the chromosome as homogeneously staining regions (HSRs). One limitation and concern about AA is regarding the accuracy or correctness of distinguishing the ecDNA from the other two classes of HSR-like amplifications (CNC and linear). It is unclear to me that whether this issued has been addressed and improved in the updated AA. According to the results provided in Figure 2c, it doesn't seems that this has been resolved in the new version of AA.
4. ecContext
The conceptional introduction of ecDNA context based on their potential generative mechanisms is excellent. This will facilitate the better interpretation, study and understanding of ecDNA in cancer. The methods section has provided very detail description of the six types of ecDNA contexts based on their generative mechanisms. However, one major concern regarding the imputation of ecDNAs into such contexts is based on the assumption that the ecDNA in the tumor tissue (cells) is homogenous. Biologically, cancer cells and their genomes are highly heterogeneous in tumor tissues, particularly in tumors with advanced stages. For example, the ecDNA defined as heavily rearranged unichromosomal (HRUC, Figure 3f) could also be explained by cancer cell heterogeneity, comprising several ecDNAs within this genomic region of the tumor cells. If one inspecting the coverage of ecDNA amplicon, the depth diversity among the different genomic blocks within the ecDNA can be an indicative of heterogeneity.
5. Other minor corrections
5.1 Figure 2c, the figure legend should include the information that these 43 cancer cell lines are different from those benchmarking cancer cells used in Figure 2a-b. The results in Figure 2c do suggest that the current AA pipeline still cannot distinguish ecDNA from HSRs.
5.2 Regarding the truncated genes on ecDNA, PVT1 is provided as an example (Figure 3i and j). However, since PVT1 is co-amplified with MYC, the data provided is not sufficient to conclude that the truncated ecDNA gene has oncogenic effect and positive selection effect. Similarly, the truncated SYT1 gene on ecDNA is within the same ecDNA amplicon of MDM2 (check from the AmpliconRepository database).
5.3 Furthermore, some of the truncated ecDNA genes, e.g. RPSAP52, MGAT4C, ecDNA gene could not be found in the AmpliconRepository.
5.4 Supplementary Figure 4b. one typo in the illustrative heavily rearranged ecDNA from single chromosome ("p" should be "g").

Remarks on code availability:
We have checked all the codes provided in GitHub and tested them with in-house WGS data from human cancers. There are enough and very detail instructions for using the codes. These codes are usable resource for the community and outperform the older version of AA/AC.

Although we cannot offer to publish your manuscript, I suggest that you consider Nature Communications as a suitable venue for this work. To transfer your manuscript, please use our manuscript transfer portal. You will not have to re-supply manuscript metadata and files, unless you wish to make modifications. For more information, please see our manuscript transfer FAQ page.

** For Nature Portfolio general information and news for authors, see http://npg.nature.com/authors.

Version 2:

Decision Letter:

Our ref: NMETH-BC55179B-Z

24th Apr 2026

Dear Dr. Bafna,

Thank you for submitting your revised manuscript "AmpliconSuite: an end-to-end workflow for analyzing focal amplifications in cancer genomes" (NMETH-BC55179B-Z). It has now been seen by the original referees and their comments are below. The reviewers find that the paper has improved in revision, and therefore we'll be happy in principle to publish it in Nature Methods, pending minor revisions to satisfy the referees' final requests and to comply with our editorial and formatting guidelines.

We are now performing detailed checks on your paper and will send you a checklist detailing our editorial and formatting requirements within two weeks or so. Please do not upload the final materials and make any revisions until you receive this additional information from us.

TRANSPARENT PEER REVIEW
Nature Methods offers a transparent peer review option for new original research manuscripts submitted from 17th February 2021. We encourage increased transparency in peer review by publishing the reviewer comments, author rebuttal letters and editorial decision letters if the authors agree. Such peer review material is made available as a supplementary peer review file. **Please state in the cover letter 'I wish to participate in transparent peer review' if you want to opt in, or 'I do not wish to participate in transparent peer review' if you don't.** Failure to state your preference will result in delays in accepting your manuscript for publication.

Please note: we allow redactions to authors' rebuttal and reviewer comments in the interest of confidentiality. If you are concerned about the release of confidential data, please let us know specifically what information you would like to have removed. Please note that we cannot incorporate redactions for any other reasons. Reviewer names will be published in the peer review files if the reviewer signed the comments to authors, or if reviewers explicitly agree to release their name. For more information, please refer to our FAQ page.

ORCID
IMPORTANT: Non-corresponding authors do not have to link their ORCIDs but are encouraged to do so. Please note that it will not be possible to add/modify ORCIDs at proof. Thus, please let your co-authors know that if they wish to have their ORCID added to the paper they must follow the procedure described in the following link prior to acceptance:
https://www.springernature.com/gp/researchers/orcid/orcid-for-nature-research

Author names using non-Roman characters
Nature Portfolio journals can support presentation of author names using non-Roman characters in the HTML version of the article. If you wish to, please include author names in parentheses after the Roman-character spelling; see example online here. Currently supported scripts are: Arabic, Chinese, Cyrillic, Devanagari, Greek, Hebrew, Hangul, Japanese and Persian. You will be asked to verify the rendering is correct at proof stage.

Thank you again for your interest in Nature Methods. Please do not hesitate to contact me if you have any questions. We will be in touch again soon.

Sincerely,
Madhura

Madhura Mukhopadhyay, PhD
Senior Editor
Nature Methods

Reviewer #2 (Remarks to the Author):

We would like to thank the authors for carefully addressing our concerns. We are convinced that AmpliconClassifier will make a great contribution to the field. We have no further comments.

Reviewer #3 (Remarks to the Author):

In this revision, the authors have addressed majority of my previous concerns and comments. However, one of my major concerns for AmpliconSuite is still its novelty and significance as a novel and disruptive method for the field.

Just to be fair to the authors for the great amounts of work and effort that have been investigated for the study, I have carried out code validation and pipeline parsing for AmpliconSuite. The whole pipeline could be executed successfully and operated smoothly using the codes provided via GitHub. So, from a reproducibility point of view, I can confirm that pipelines are reproducible. Agreed with the authors' claim regarding the mapping speed of the AA, we observed some improvement but not dramatic. Another implementation that the current AmpliconSuite has installed is a more detailed stratification of the ecDNA context. I would hope that the authors could provide more biological and cancer insights in the revision. However, the current applicational demonstration of AmpliconSuite with ecDNA gene co-amplification and tissue-type based cancer stratification still belong to the nature of descriptive observations, rather than mechanical insights that can only be achieved with AmpliconSuite, substantially lacking validations.

However, I see the study more valuable from a resource point of view instead of a significantly improved or novel method for the ecDNA and cancer biology field. The aggregated pipelines used by AmpliconSuite enable one-stop identification and characterization of focal amplification from WGS data. The online repository as commended before is another useful resource for the field. The current repository contains 8772 samples from 14 projects. However, the online repository is more a platform or app for visualization of AA outputs. The analytical and applicational values of the online platform are still limited.

Reviewer #3 (Remarks on code availability):

The codes provided contain enough details for reproducibility. We are able to run the code locally and successfully.

Version 3:

Decision Letter:

15th Sep 2026

Dear Professor Bafna,

I am pleased to inform you that your Brief Communication, "AmpliconSuite: an end-to-end workflow for analyzing focal amplifications in cancer genomes", has now been accepted for publication in Nature Methods. The received and accepted dates will be Feb 22, 2024 and Sep 15, 2026. This note is intended to let you know what to expect from us over the next month or so, and to let you know where to address any further questions.

Over the next few weeks, your paper will be copyedited to ensure that it conforms to Nature Methods style. Once your paper is typeset, you will receive an email with a link to choose the appropriate publishing options for your paper and our Author Services team will be in touch regarding any additional information that may be required.

If you have any questions about our publishing options, costs, Open Access requirements, or our legal forms, please contact ASJournals@springernature.com

Once proofs are generated, they will be sent to you electronically and you will be asked to send a corrected version within 48 hours. It is extremely important that you let us know now whether you will be difficult to contact over the next month. If this is the case, we ask that you send us the contact information (email, phone and fax) of someone who will be able to check the proofs and deal with any last-minute problems.

If, when you receive your proof, you cannot meet the deadline, please inform us at rjsproduction@springernature.com immediately.

If you have posted a preprint on any preprint server, please ensure that the preprint details are updated with a publication reference, including the DOI and a URL to the published version of the article on the journal website.

You may wish to make your media relations office aware of your accepted publication, in case they consider it appropriate to organize some internal or external publicity. Once your paper has been scheduled you will receive an email confirming the publication details. This is normally 3-4 working days in advance of publication. If you need additional notice of the date and time of publication, please let the production team know when you receive the proof of your article to ensure there is sufficient time to coordinate. Further information on our embargo policies can be found here:
https://www.nature.com/authors/policies/embargo.html

Authors may need to take specific actions to achieve compliance with funder and institutional open access mandates. If your research is supported by a funder that requires immediate open access (e.g. according to Plan S principles) then you should select the gold OA route, and we will direct you to the compliant route where possible. For authors selecting the subscription publication route, the journal's standard licensing terms will need to be accepted, including self-archiving policies. Those licensing terms will supersede any other terms that the author or any third party may assert apply to any version of the manuscript.

If you are active on X or Bluesky, please e-mail me your and your coauthors' handles so that we may tag you when the paper is published.

To assist our authors in disseminating their research to the broader community, our SharedIt initiative provides you with a unique shareable link that will allow anyone (with or without a subscription) to read the published article. Recipients of the link with a subscription will also be able to download and print the PDF. As soon as your article is published, you will receive an automated email with your shareable link.

Please note that you and your coauthors may order reprints and single copies of the issue containing your article through Springer Nature Limited's reprint website, which is located at http://www.nature.com/reprints/author-reprints.html. If there are any questions about reprints please send an email to author-reprints@nature.com and someone will assist you.

You can now use a single sign-on for all your accounts, view the status of all your manuscript submissions and reviews, access usage statistics for your published articles and download a record of your refereeing activity for the Nature journals.

Please feel free to contact me if you have questions about any of these points.

Best regards,
Madhura

Madhura Mukhopadhyay, PhD
Team Leader and Senior Editor
Nature Methods

** Visit the Springer Nature Editorial and Publishing website at www.springernature.com/editorial-and-publishing-jobs for more information about our career opportunities. If you have any questions please click here.**

Open Access This Peer Review File is licensed under a Creative Commons Attribution 4.0 International License, which permits use, sharing, adaptation, distribution and reproduction in any medium or format, as long as you give appropriate credit to the original author(s) and the source, provide a link to the Creative Commons license, and indicate if changes were made. In cases where reviewers are anonymous, credit should be given to 'Anonymous Referee' and the source.
The images or other third party material in this Peer Review File are included in the article's Creative Commons license, unless indicated otherwise in a credit line to the material. If material is not included in the article's Creative Commons license and your intended use is not permitted by statutory regulation or exceeds the permitted use, you will need to obtain permission directly from the copyright holder.
To view a copy of this license, visit https://creativecommons.org/licenses/by/4.0/

Response to Reviewers

We thank the reviewers for their thoughtful and constructive feedback, which has substantially strengthened our manuscript, as well as for their patience in our revision process. Following this summary of our changes, we provide a point-by-point response to each comment, with changes to the manuscript indicated by line numbers in the revised text.

Summary of Major Revisions

1. Expanded cytogenetic validation establishes the field's largest metaphase FISH benchmarking dataset for ecDNA

We expanded our metaphase FISH validation from 66 probed genes across 43 cell lines to **175 probed genes across 72 cell lines/models**, the largest single cytogenetic validation dataset in the literature. This this balanced dataset (56 ecDNA, 52 non-native HSR, 39 native HSR, 25 unknown HSR, 3 indeterminate for ecDNA) enabled rigorous performance comparison across three AmpliconClassifier versions, demonstrating progressive improvements culminating in AC v1.5.1 achieving 94.6% sensitivity, 94.2% specificity, and 91.4% precision as well as improved detection of focal amp boundaries. Figure 2 has been completely revised around these findings and comparisons with optical genome mapping to show robust BFB detection.

2. Figure 3 redesigned to showcase novel biological discoveries enabled exclusively by updates reported in this paper

Following reviewer feedback requesting clear demonstration of what the current version enables, we replaced the original Figure 3 with analyses revealing novel cancer biology:

- **ecContext validation on experimentally-induced ecDNA**: Using landmark studies where ecDNA was induced with known formation mechanisms (Pradella et al., Nature 2025; Shoshani et al., Nature 2021), we found strong concordance **(11/11 samples) for identifying simple excisional ecDNA** and **(20/23 samples) for chromothripsis-derived ecDNA**, demonstrating that ecContext can reliably distinguish formation mechanisms.

- **CDK4-MDM2 co-amplification is an ecDNA-driven phenomenon**: Analysis of 61 co-amplifying samples revealed **72% are mediated by ecDNA** (64% multi-interval ecDNA + 8% separate ecDNA). Regional analysis also revealed ecDNA selectively retains functional neighbors. YEATS4 (chromatin remodeler) was co-amplified with MDM2 in 79% of cases despite 514 kb separation.

- **Tissue-specific formation mechanisms**: Among 2,413 pan-cancer ecDNA events, 36.8% formed by simple excision and 48.4% by chromothripsis-associated mechanisms, with prostate/brain cancers enriched for excision while skin cancers and sarcomas showed chromothripsis enrichment, aligning with known high chromothripsis rates in these tissues.

3. Comprehensive methodological justification

In response to questions about AmpliconClassifier's parameter choices and validation strategy, we:

- **Demonstrated parameter optimality**: Systematic sweeps across 12 key parameters using the FISH-validated dataset show default values achieve optimal or near-optimal F1 scores (Supplementary Table 4)
- **Documented all classification logic**: Created comprehensive parameter documentation (https://github.com/AmpliconSuite/AmpliconClassifier/blob/main/AC_config_parameters.md) explaining all 19 configurable parameters and their biological rationale
- **Validated against orthogonal methods**: WGS data compared to optical genome mapping demonstrated high concordance in BFB detection (revised Figure 2g).
- **Comprehensive simulations**: Benchmarked ecDNA detection against *in silico* purity dilution, analyzed ecDNA detection in simulated WGS with simple and chromothriptic ecDNA structures present, and analyzed the effects of downsampling.

AmpliconSuite has gained excellent community traction: 15 large-scale studies (50+ samples each) analyzing 8,332 samples were published or appeared as preprints in 2025 alone, with 10 conducted independently of tool develop input. AmpliconRepository.org continues to expand and now hosts >15,000 samples.

Reviewer #1:

Remarks to the Author:

The authors describe the AmpliconSuite package that they developed; this builds on their earlier published AmpliconArchitect method and adds a classifying algorithm for the detected amplicons. AmpliconSuite is then applied to ~2500 WGS samples from cell line and patient data.

AmpliconClassifier and the methodology behind the classification have already been used by the authors in earlier publications (Kim et al., Nature Genetics, 2020 and Luebeck et al., Nature, 2023). Both these publications include the analysis of large datasets after classification using AmpliconClassifier.

Response: We thank the reviewer for their summary of our work and greatly appreciate the reviewer's recognition of the prior utility of this toolset. While earlier versions of AmpliconClassifier have been used in prior publications, the current manuscript presents substantial methodological advances (e.g., ecContext, improved classification performance), benchmarked on what is now the largest metaphase FISH dataset in the field, as well as showing novel biological discoveries enabled by these improvements and the aggregation of results via our companion web platform AmpliconRepository.org.

Major Comments:

1. As these publications show, a tool like AmpliconClassifier is very important when studying ecDNA-based cancers. For this manuscript to be a strong addition to the ecDNA literature, it should in figure 2 very clearly demonstrate how the current iteration of AmpliconClassifier outperforms the versions that were used in Kim et al. and Luebeck et al. In figure 3, there should be a similar comparison: which results are improved in upgrading to the current iteration of AmpliconClassifier?

Response: This is a key point raised by the reviewer and it has led us to strongly re-evaluate how we show the improvements in the tool. In the prior version of the manuscript, our intention was to demonstrate our many contributions distinct from improvements to AmpliconClassifier itself (including the building and deployment of AmpliconRepository). However, AmpliconClassifier has undergone many improvements, and we agree with the reviewer that it will be good to re-focus on these new developments, and show how the classifications have improved in the latest version of the tool.

To address this, we have expanded our set of cell lines with cytogenetically validated ecDNA status. Previously, we relied on a set of 66 total metaphase FISH-probed genes across 43 different cell lines as a truth set for comparing our classification heuristics. We performed **extensive** additional validation experiments, **expanding this dataset by >100 additional probes** to a total of 175 metaphase FISH-probed genes across 72 different cell lines or models**,** incorporating new data (85 probed genes) with prior results from five different studies.

Importantly, this new data set contains a close balance of different cytogenetic amplification types; 56 ecDNA (DM), 3 indeterminate for ecDNA, 52 non-native HSR (homogeneously staining region) - where the amplified gene was located on a different centromere than its corresponding centromeric probe, 39 native HSR, and 25 unknown HSR types - where the centromere was not concurrently probed with the gene.

We first wish to note a particular challenge of evaluating ecDNA detection in cancer cell lines. The re-integration of ecDNA into chromosomal DNA is a common phenomenon that is observed in cancer cell lines, and may occur spontaneously (Alitalo et al., PNAS, 1983; PMID: 6300869) or be induced by changes to selective pressures (Nathanson et al., Science 2014; Song et al., Cancer Discovery, 2022). Prior analysis of HSR integration points is limited, but based on existing findings and a null model of random re-integration, a non-native integration location is expected (e.g., Luebeck et al., Nature Communications 2020; Song et al., Cancer Discovery, 2022). In our validation data, when ecDNA was identified by WGS, and a non-native HSR was observed in the amplicon's FISH data, we marked these cases as neutral in the calculation of performance metrics. Such ecDNA-derived amplifications still carry the signatures of ecDNA in their SV "fossil records" (revised Figure 2c).

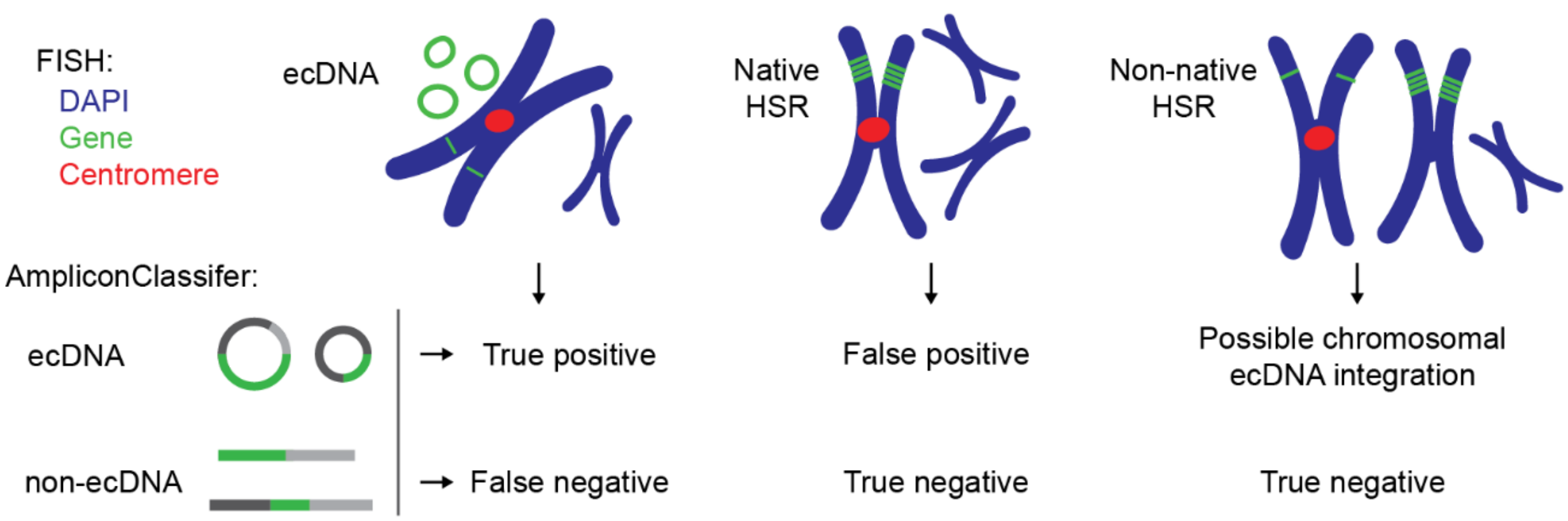

By contrast, in patient tumor samples, the landscape of selective pressures is heterogeneous across the tumor and over time. Thus, even if a significant fraction of cells were converted to re-integrated ecDNA and appeared as HSR, we argue that some ecDNA cells would likely remain and could be re-selected when selective pressures changed again (Nathanson et al., Science, 2014).

On the panel of 175 amplifications with ecDNA status validated by metaphase FISH we then applied three versions of AmpliconClassifier; AC 2020 (Kim et al., Nature Genetics 2020), AC v0.4.13 (Luebeck et al., Nature 2023), and the most recent version AC v1.5.1. The latest version showed the best performance, highlighting the improvements to the method over time.

Metric	AC 2020	AC v0.4.13	AC v1.5.1
Sensitivity	92.9%	92.7%	94.6%

Specificity	85.2%	93.1%	94.2%
Precision	81.3%	89.5%	91.4%

We have incorporated these findings into a heavily revised Figure 2 which illustrates the methodology for creating the truth set (Fig. 2c-e), and comparisons to prior versions of AmpliconClassifier (Fig. 2f), detailed in revised manuscript section "Expanded validation analysis."

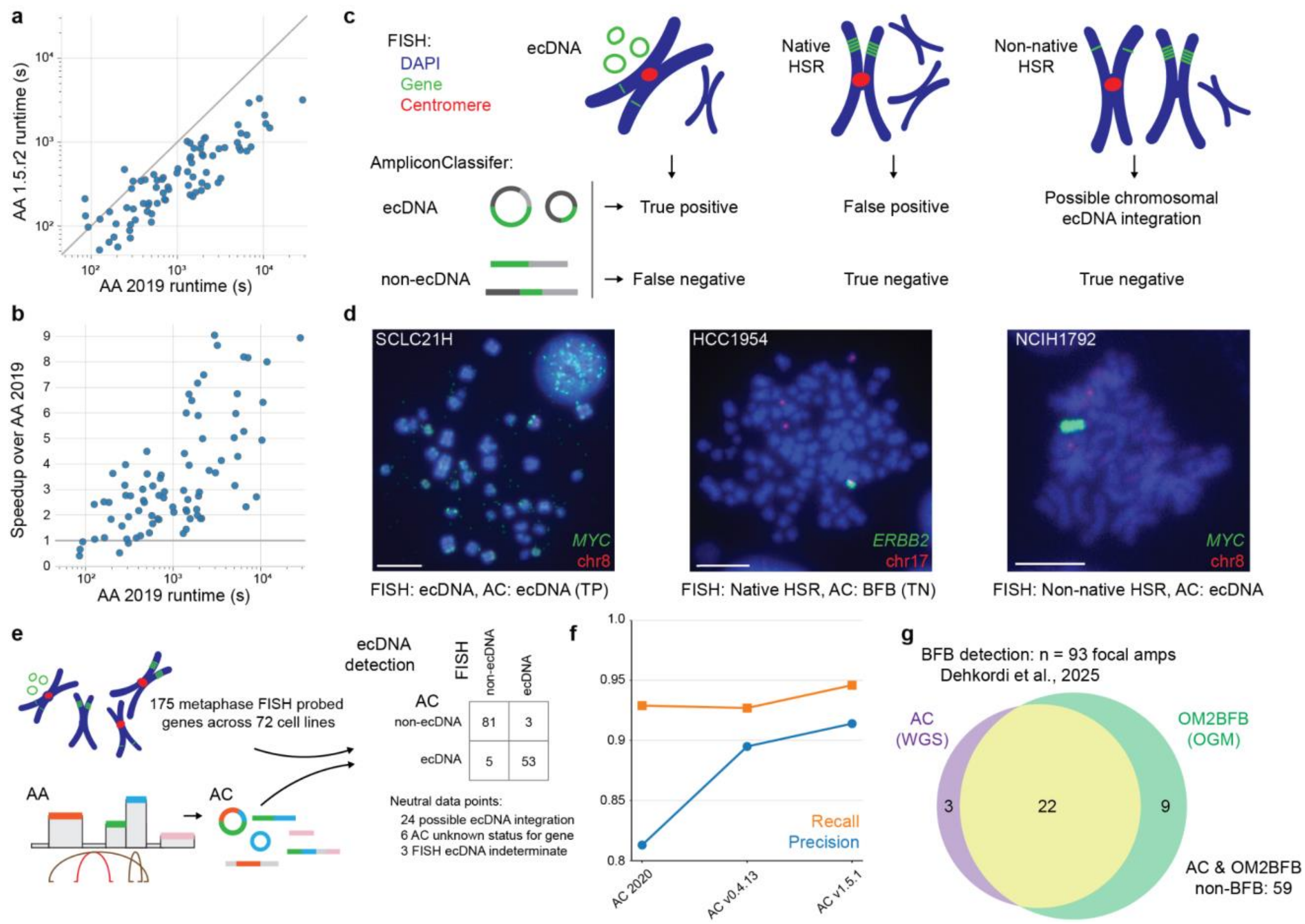

To the reviewer's second point about highlighting analyses that were made possible with newer versions of AmpliconClassifier (AC) that would not have been possible before, we want to highlight the tremendous improvement in resolution of ecDNA segment boundaries that has occurred since the initial version of AC. In the earlier version, all regions of an amplicon were assumed to belong to the ecDNA, BFB, etc. identified in the amplicon. Currently, AmpliconClassifier uses the locations of genomic cycles to identify ecDNA boundaries and separate ecDNA species within an amplicon. There is also the addition of the ecContext method to AC, which is described in response to the reviewer's comment #6.

To illustrate the improvement to ecDNA boundary identification, using the AC 2020 (Kim et al., Nature Genetics, 2020) results reported for PCAWG and TCGA, 210 / 579 ecDNA were reported to be longer than 10 Mbp, with 71 being larger than 50 Mbp. In that scheme, ecDNA regions were not separated within an amplicon, so if AA analyzed an entire chromosome arm and identified ecDNA-like cycles somewhere, the entire amplicon region would be marked as ecDNA. This can easily result in conflating karyotypic abnormalities or arm-level gains with bona fide ecDNA. By contrast when applying the latest version of AmpliconClassifier (v1.5.1) to the 2024 re-analysis of PCAWG and TCGA, the resulting properties of the amplifications are much more reasonable. Only 35 of 659 identified ecDNA exceed 10 Mbp in length and none are longer than 50 Mbp. These changes affect all amplicon types, not only ecDNA. This improved classification of genomic regions also manifests in other important downstream aspects - including gene contents analysis, amplicon complexity scores and so forth.

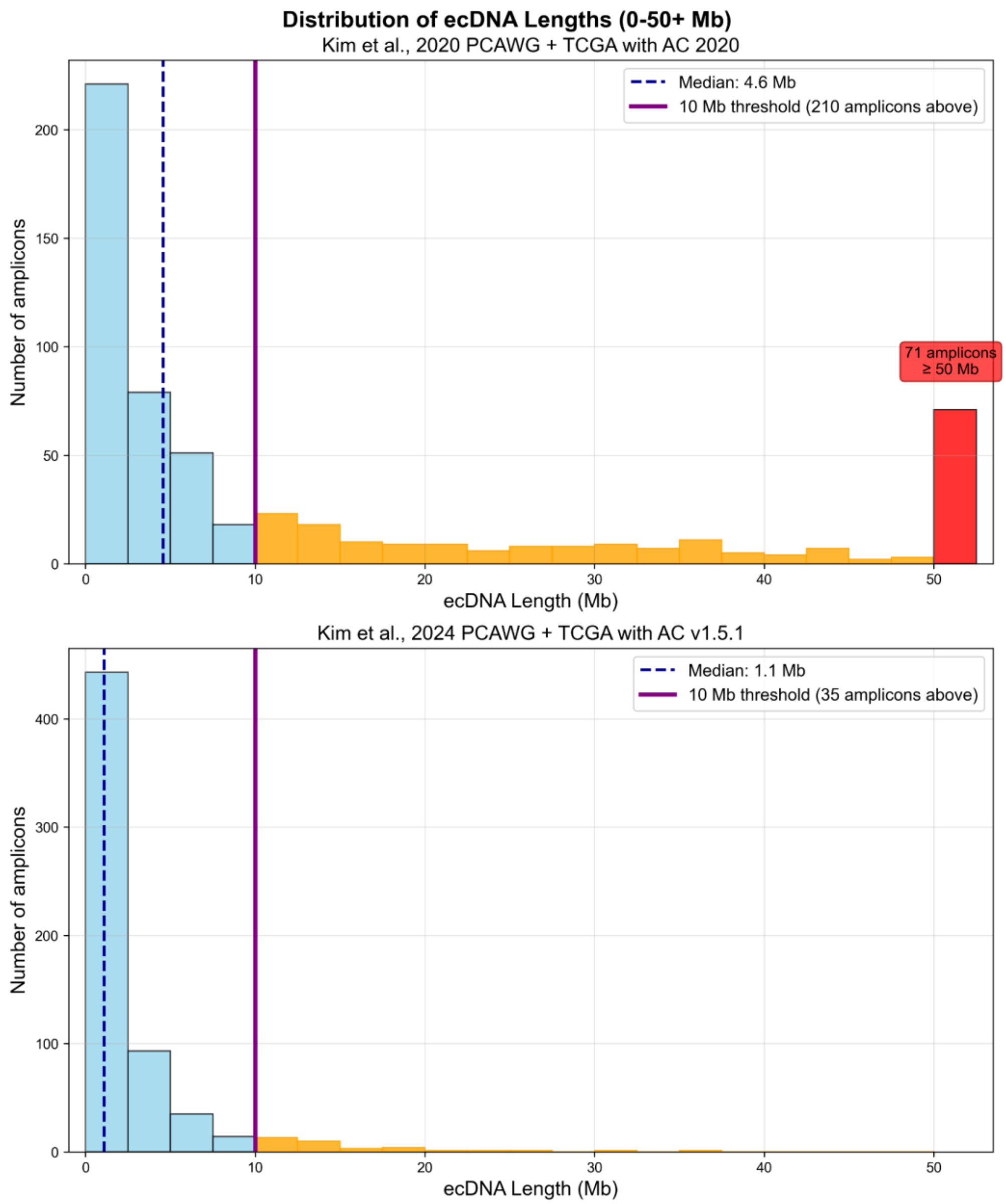

To highlight these changes at a detailed level, we delved into a few particular samples which have changed from an ecDNA label in Kim et al., 2020 to non-focal amp labels with newer improved versions of AC reported in the table below. Notably, all of those reported regions were extremely large - over 50 Mbp - and represented entire chromosome arms. With the exception of the last example (which showed chromothriptic-like properties), these are better characterized as chromosome-arm level gains than focal amplifications.

PCAWG ID	Genomic Coordinates	AC 2020	AC v1.5.1
SA501395	6:39681-57330015	Circular	No fsCNA
SA542651	7:40386-158817500	Circular	No fsCNA
SA541952	7:40386-158817500	Circular	No fsCNA
SA543605	5:0-180689978,17:0-81195209,GL000220.1:0-161802	Circular	No fsCNA
SA542014	17:7582251-22199999	Circular	Complex non-cyclic

The revised manuscript focuses on newly added features as requested by reviewers, so this complete result is not directly included. However, it is reflected in the overall improved performance of AC. However we have added a description of the process for resolving amplicon boundaries to the Methods section (lines 611-616).

2. In figure 2, the authors describe the performance of AmpliconArchitect and AmpliconClassifier. The performance improvement of AmpliconArchitect looks impressive. I am less convinced with the methodology behind AmpliconClassifier, for two reasons: first of all, the classification methodology relies on a number of filtering steps and neither the specific choices nor the parameter values are explained. Second of all, since this is a classifier the authors should describe their training and test dataset, how the training set is used to determine the parameter values, and how the performance is on the training and on the test set.

We thank the reviewer for this important observation. We wish to first highlight that the analysis of ecDNA at a sequence level is a relatively new scientific field. There is very little gold standard data providing fully resolved ecDNA sequences. Even imaging-based approaches for ecDNA detection based on FISH are scarce, scattered across different sources, and do not represent a large enough dataset for robust machine learning. Our new set of 175 metaphase FISH-stained gene probes with contemporaneous WGS, that we will release with this study, are intended as a resource for the research community. We believe that this effort will jump-start the collection of larger-scale truth datasets, aiding future algorithm development.

For the aforementioned reasons, AmpliconClassifier employs a rule-based classification system rather than a machine learning classifier. The default parameters used by AC were determined through:

- Empirical optimization against metaphase-FISH validated samples (our "gold standard" ground truth);
- Theoretical constraints based on known focal amplification biology;
- Iterative refinement through analysis of >2,800 cancer samples.

While we don't have traditional training/test sets as in supervised machine learning, our validation approach uses:

- The FISH-validated dataset for parameter optimization;
- Independent cohorts for performance evaluation (as shown in Figure 2);
- Cross-validation against orthogonal methods including long-read sequencing (Zhu, Jones et al., Genome Research, 2024) and optical genome mapping (Dehkordi et al., Nature Communications, 2025), which we describe below.

We believe this rule-based approach offers advantages including interpretability of classification decisions and the ability to adjust parameters for specific biological contexts, while maintaining robust performance across diverse cancer types.

We have revised the methods section and supplementary materials to clarify this distinction and better explain our methodology as follows:

1. Added a comprehensive description of the parameter determination methodology in the revised methods (lines 589-605):

   "The classification rules and their associated parameters were developed through iterative refinement based on: (1) empirical observations from metaphase-FISH validated samples, (2) theoretical understanding of focal amplification mechanisms. The complete set of 19 configurable parameters governing these rules is provided in the software distribution as a JSON configuration file (AC codebase default_config.json file), with default values established through parameter optimization against our FISH-validated benchmark dataset and manual curation of predictions on large-scale cancer datasets such as CCLE, PCAWG, TCGA. A complete description of the parameters and how each is used is available at https://github.com/AmpliconSuite/AmpliconClassifier/blob/main/AC_config_parameters.md.
   In brief, the parameters control various aspects of the classification including: minimum size thresholds for valid amplifications (e.g., minimum cycle size of 5kb), copy number thresholds for different amplification classes (e.g., CN > 4.5 for focal amplifications), structural variant filtering criteria (e.g. 25kbp as the maximum distance between inversion endpoints to call an SV a foldback), and proportional thresholds for classifying specific amplification types such as BFB and ecDNA (e.g. 0.6 as the proportion of total CN-weighted path length supporting BFB to classify a focal amp as being BFB). Goodness of parameter choices was evaluated by parameter sweeps (Supplementary Table 4)."

2. Regarding the filtering steps and parameter values, the classification relies on 19 configurable parameters that govern various filtering and threshold decisions. We have created a comprehensive description of the complete parameter configuration file online, with descriptions of each parameter's role, its default value and what functions use it: https://github.com/AmpliconSuite/AmpliconClassifier/blob/main/AC_config_parameters.md

3. Included parameter sensitivity analysis (Supplementary Table 4) showing systematic sweeps of the 9 most sensitive parameters against our FISH-validated benchmark set. This is presented in more detail in response to the following comment #3 by the reviewer.

In lieu of a classical testing dataset, we examined the performance of AC against results from a large-scale optical genome mapping study (Dehkordi et al., Nature Communications, 2025) that identified breakage-fusion-bridge (BFB) focal amplifications, where matched WGS data was also available for 93 focal amplifications. Using AC v1.5.1, we compared WGS-based predictions to OM2BFB calls assumed as truth. We found BFB detection using AC had: Sensitivity (Recall): 0.71, Specificity: 0.95, Precision: 0.88. These data showcase the strength of AC because they were achieved with short-reads, whereas the Dehkordi study was conducted using ultra-long optical genome maps (~150-200kbp per map). These results are presented in revised Figure 2g and Supplementary Table 3.

	OM2BFB: BFB	OM2BFB: Not BFB
AC: BFB	22	3
AC: Not BFB	9	59

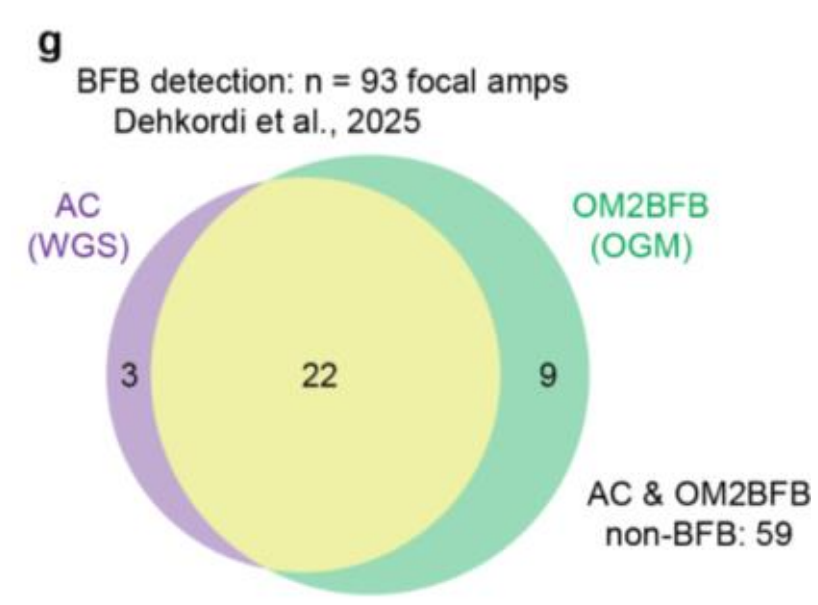

3. There should be a systematic approach behind determining the parameter values, and the performance of the classifier should be measured on a separate test dataset.

Response: This is a critically important analysis and we thank the reviewer for suggesting it. To demonstrate that we have selected reasonable parameters, we performed a parameter sweep of 12 important parameters for AC, deploying it on our new cytogenetically validated dataset of the 175 metaphase FISH-probed focal amplifications. We then computed an F1 score (harmonic mean of precision and recall) at each setting. We have created Supplementary Table 4 which reports these results. We reproduce the table below and we provide the following graphical representation for the reviewer as well. Importantly, the parameters we had chosen for AmpliconClassifier are at, or are near the top of the F1 plots, and make sense in the context of the known properties of ecDNA. The meaning of each AC parameter is described in https://github.com/AmpliconSuite/AmpliconClassifier/blob/main/AC_config_parameters.md.

parameter	default_f1	best_f1	f1_difference	default_value	best_value_range
anyCycContcut	0.94805	0.94805	0	10000	0
compCycContCut	0.94805	0.94805	0	50000	0-50000
cycCut	0.94805	0.94805	0	0.12	0.1-0.3
fb_break_weight_prop	0.94805	0.96053	-0.01247	0.3	0
fb_dist_cut	0.94805	0.94805	0	25000	10000-50000
max_nonbfb_break_weight	0.94805	0.95425	-0.0062	0.5	0.7-1.0
max_segdup_size	0.94805	0.94805	0	1000000	10000-2010000
minCycleSize	0.94805	0.94805	0	5000	0-25000
min_bfb_cycle_weight_ratio	0.94805	0.95425	-0.0062	0.6	0.0-0.3
min_fb_read_prop	0.94805	0.94805	0	0.25	0.05-0.5
segdup_max_extra_fraction	0.94805	0.94805	0	0.25	0.0-1.0
tot_min_del	0.94805	0.94805	0	5000	1000-10000

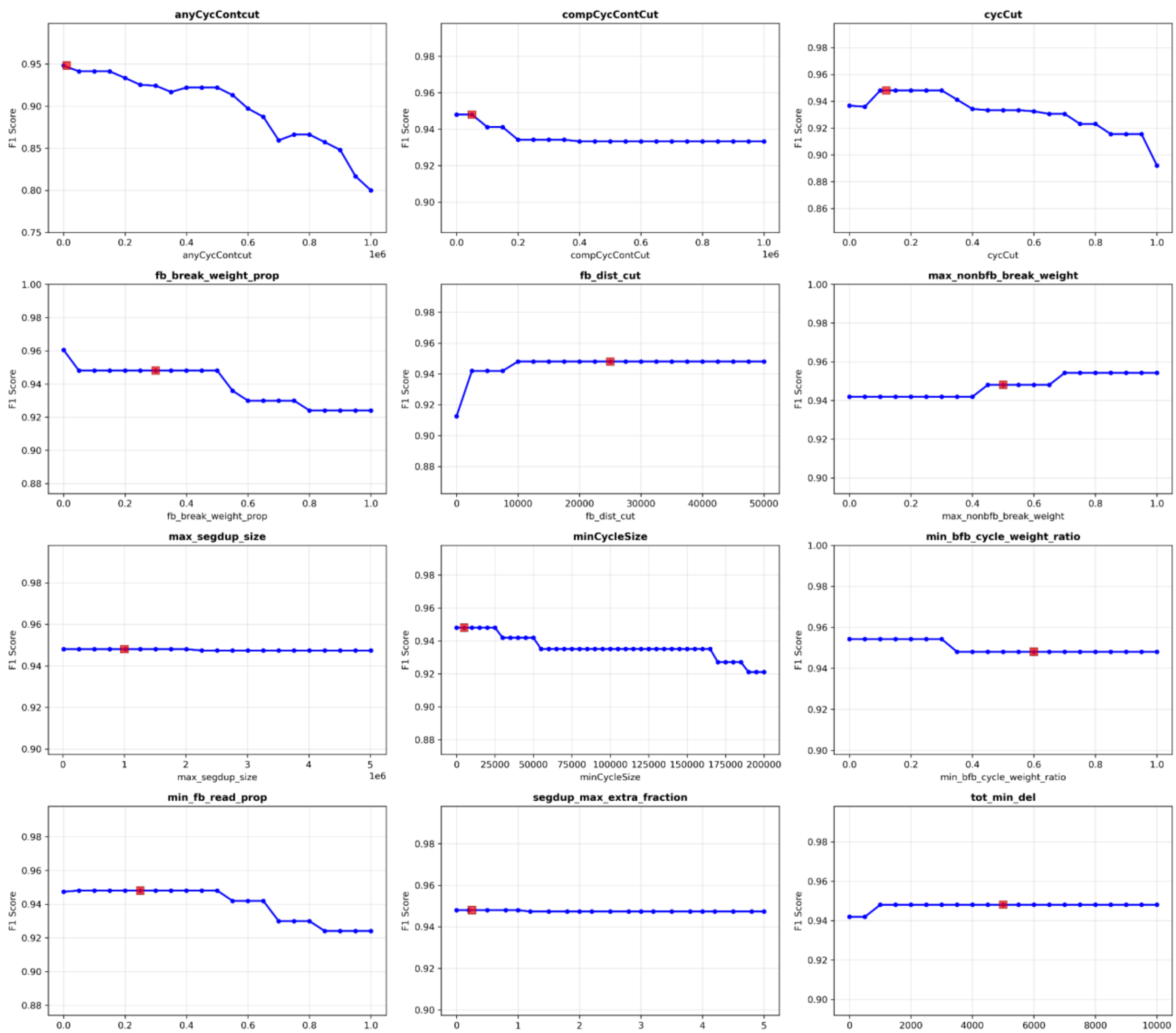

4. In my understanding, an HSR that consists of amplicons that are linearly integrated in tandem would generate almost exactly the same signal as ecDNA. The authors should explain and simulate why this type of linear amplification is not classified as ecDNA.

Response: This is a very important distinction and a common question we receive, and so we thank the reviewer for raising it. The reviewer's intuition is correct that linear tandem duplications could, in principle, generate signals similar to ecDNA in short-read sequencing data. However, our classification approach leverages key structural differences that distinguish these mechanisms, and emerging experimental evidence suggests that ecDNA-derived HSR amplifications are typically not composed of simple tandem duplications.

To improve detection especially w.r.t the reviewer comments, AmpliconClassifier v1.5.1 uses a filtering strategy based on two critical features to differentiate ecDNA from linear tandem duplication structures. First, smaller circular amplicons (<500 kbp) which have only a simple head-to-tail (tandem dup or ecDNA-like) SV that show copy numbers not significantly above 2-fold beyond the background genomic level are classified as linear amplifications by AC rather than ecDNA. This is based on the principle that these head-to-tail connections showing modest copy number gains against background are readily explained by local duplication events. Conversely, high copy number circular amplicons require extensive breakpoint reuse, meaning the same genomic junctions must be traversed many times to generate the observed copy number profile. While this pattern is expected and observed in ecDNA (where a circular molecule replicates autonomously to high copy number), it is unlikely to arise through tandem duplication mechanisms, which would require the same precise breakpoints to be used repeatedly during independent duplication events. We have added a description of the tandem-dup filtering to the manuscript Methods section lines 576-580.

We illustrate how we filter a validated duplication event with an example in our validation data. The T47D cell line harbors a chr16 focal amplification which we analyzed with WGS and previously analyzed with optical genome mapping.

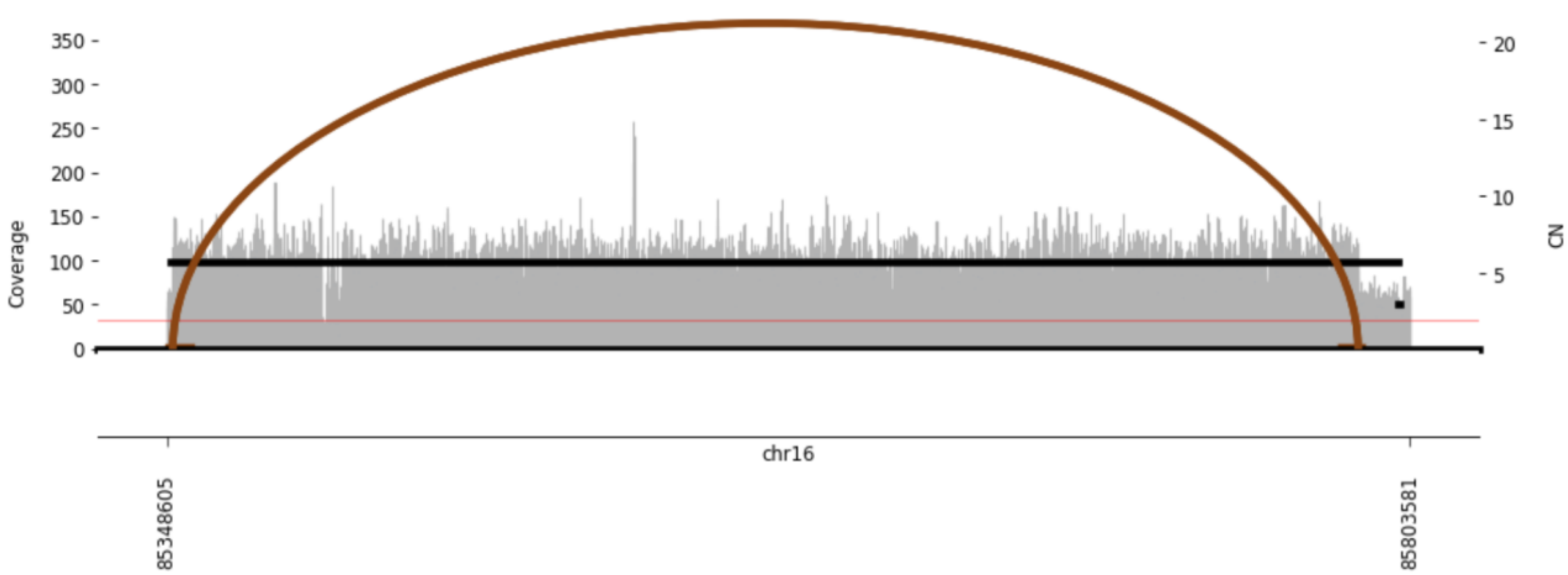

Figure above: T47D chr16 focal amp from CCLE (hg38 coordinates), which AC v1.5.1 classifies as a “Linear amplification”

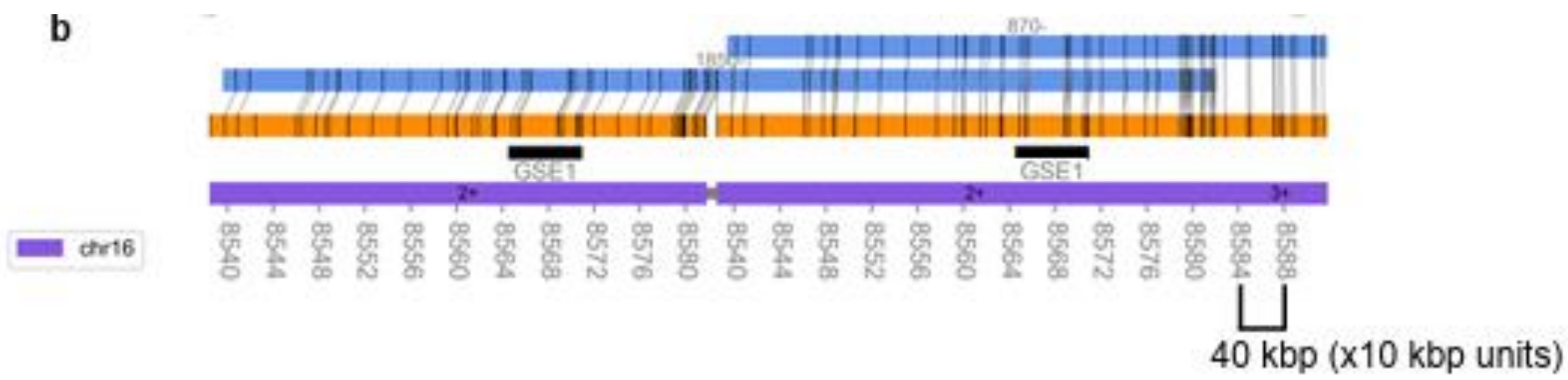

Figure above: (reproduced from Luebeck et al., Nature Communications, 2020 Supplementary Figure 13b), an optical genome mapping-based reconstruction of T47D chr16 focal amp (hg19 coordinates), using Bionano optical genome map contigs, reveals that the event is consistent with a duplication anchored to the neighboring regions, not a circular ecDNA.

While relatively little is known about the fine-structure of ecDNA-derived HSR amplifications, emerging evidence suggests that HSR amplifications are often not comprised of simple linear tandem duplications. When ecDNA integrates into chromosomes to form HSRs, the resulting structures are often highly complex and retain signatures of their circular origin (e.g. COLO320DM/HSR). An illustrative example comes from Song et al., Cancer Discovery, 2022 (Figure below), showing an ecDNA-derived HSR amplification reconstructed by optical genome mapping. The structure reveals extensive complexity beyond what would be expected from tandem duplications, indicating that even after chromosomal integration, these amplifications maintain the structural hallmarks of their ecDNA origin rather than reorganizing into simple linear arrays.

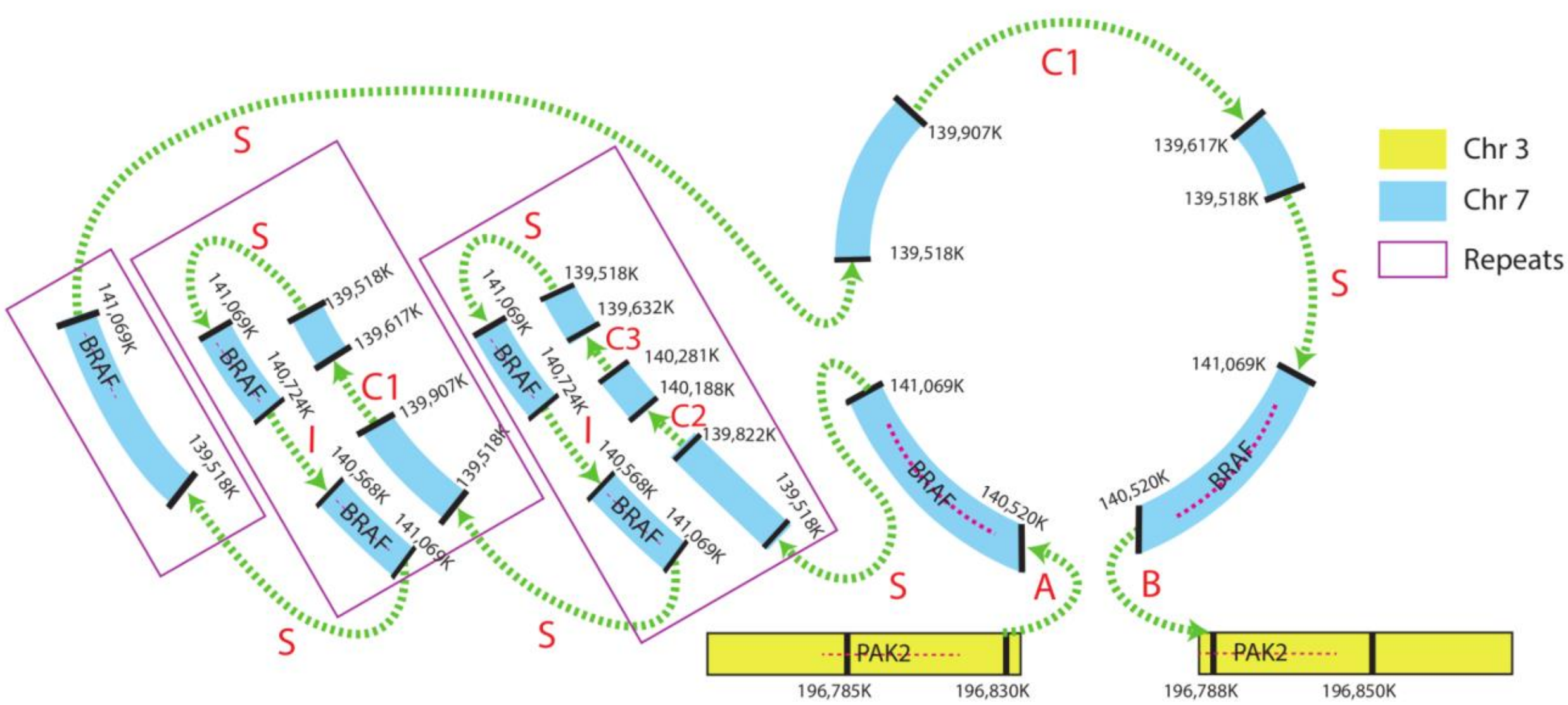

Figure above: Reproduced from from Song et al., Cancer Discovery, 2022. "Plasticity of Extrachromosomal and Intrachromosomal BRAF Amplifications in Overcoming Targeted Therapy Dosage Challenges." The reconstruction shows the complex structure of an ecDNA-derived HSR

amplification, as analyzed by optical genome mapping, indicating that it is not typically comprised of tandem duplications of the ecDNA.

Together, these observations support our classification approach: high copy number ecDNA exhibits extensive breakpoint reuse, inconsistent with tandem duplication mechanisms, while lower copy number simple circular structures are appropriately classified as local duplications. The structural complexity observed in HSR amplifications further suggests that the genomic architectures of integrated amplicons differ fundamentally from simple tandem arrays.

5. For figure 3, I think it would be important to show which of these results could have already been obtained by earlier iterations of AmpliconClassifier.

Response: We thank the reviewer for this insightful suggestion. We agree that the original Figure 3 did not adequately distinguish novel capabilities from existing functionality. It also did not adequately convey that this manuscript discussed capability above and beyond AmpliconClassifier, such as the development of the AmpliconRepository In fact, the first four panels of the original figure presented analyses could have been performed with earlier AmpliconClassifier versions.

In revising the manuscript, we have completely redesigned Figure 3 to focus exclusively on two novel analytical capabilities that reveal new biological insights: (1) gene co-amplification analysis (revised Figure 3a-e), enabled by our new Co-amplification Graph utility on AmpliconRepository.org, and (2) ecDNA formation mechanism classification (revised Figure 3f-j), enabled by our new ecContext method. From the original Figure 3, we retained only the ecContext work (which we have substantially expanded upon) and the finding that ecDNA shows significantly greater size variance than non-ecDNA focal amplifications (now reported in text rather than as a figure panel). We have also revised Figure 2 to better demonstrate AmpliconClassifier's performance improvements over prior versions (as suggested in the reviewer's earlier comments). These changes strengthen the manuscript by clearly highlighting the novel contributions of AmpliconSuite. Our detailed response to the reviewer’s next point (#6) describes the specific biological insights gained from these new analyses.

6. Since AmpliconClassifier has already been published, I feel it would be important to show what novel biological insights can be gained by it. In that sense, I don’t think Figure 3 does AmpliconClassifier/AmpliconSuite enough justice.

We greatly appreciate this suggestion from the reviewer and in addressing these points, we feel the manuscript has been clearly strengthened.

We completely revised Figure 3 to focus predominantly on two new types of analysis we’ve created for AmpliconSuite, which are focused on revealing novel biology of ecDNA:

(1) analysis of oncogene co-amplifications mediated by ecDNA (revised Figure 3a–e; Supplementary Figure 7d)
(2) analysis of ecDNA context, particularly how it relates to ecDNA of chromothriptic and non-chromothriptic origins (revised Figure 3f–j).

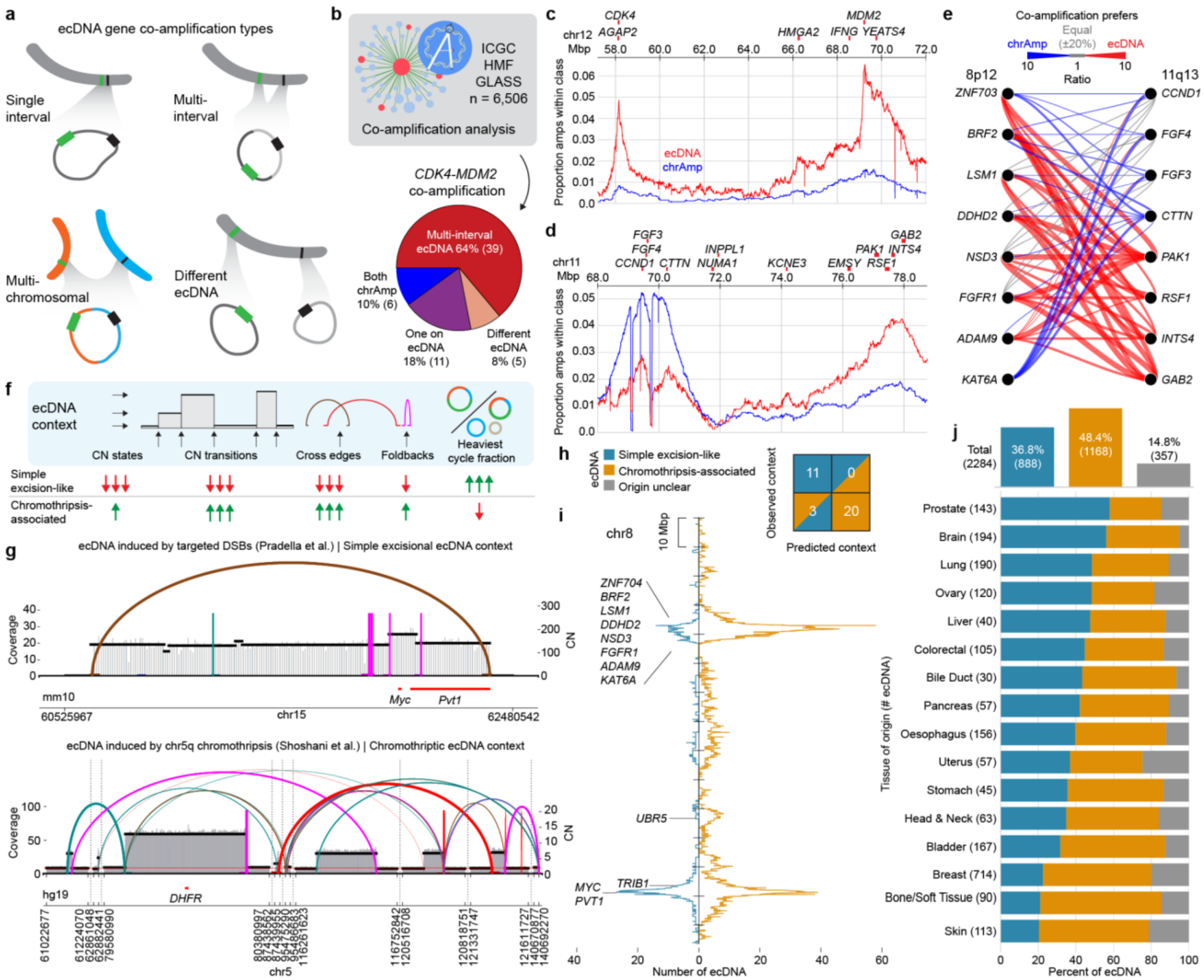

These findings are described on lines 266-396. In short, we performed comprehensive analysis of focal amplifications using 6,506 patients from TCGA, PCAWG (ICGC), Hartwig Medical Foundation, and GLASS cohorts (an increase from the 2,525 samples in the prior manuscript version), reclassifying with AmpliconClassifier v1.5.1 to identify 2,413 ecDNA events. The projects used were all downloaded directly from AmpliconRepository.org, demonstrating AmpliconRepository.org's utility for enabling large-scale biological insights.

Gene co-amplification analysis (Figure 3a-e): We developed a Co-amplification Graph utility on AmpliconRepository.org that systematically identifies how gene pairs are co-amplified on ecDNA through four mechanisms: single contiguous intervals, multiple non-contiguous intervals on the same chromosome, multi-chromosomal segments on single ecDNA, or distinct ecDNA elements. Using this tool, we discovered:

- CDK4-MDM2 co-amplification (61 samples): 64% on multi-interval ecDNA, 8% on separate ecDNA, demonstrating ecDNA's outsize role in this potent co-amplification.
- Regional analysis of chr12q14-15 showed ecDNA selectively excludes intergenic regions between CDK4-MDM2 while incorporating neighboring oncogenes (YEATS4, AGAP2).
- chr11q13 analysis revealed mechanistic preferences: CCND1/FGF3/FGF4 relatively enriched in chromosomal amplifications while PAK1/RSF1/INTS4/GAB2 preferentially amplified by ecDNA, suggesting distinct selective advantages for chromatin remodeling and signaling genes.
- Multi-chromosomal co-amplification between chr8p12 and chr11q13 oncogene clusters showed potential ecDNA-mediated functional synergy between signaling pathway components and chromatin remodeling machinery.

ecContext methodology and validation (Figure 3f-h)**:** Note that ecDNA can be formed by a variety of mechanisms (Bafna and Mischel, Annual Review of Genomics and Human Genetics, 2022). We developed ecContext to describe the structural characteristics of ecDNA and the background structural signals surrounding the ecDNA. The seven categories described by ecContext are strongly suggestive of the mechanisms of ecDNA formation. Specifically, these categories distinguish simple excision (Simple Circular, Simple Background (SCSB), two-foldback) from chromothripsis-associated mechanisms (Simple Circular Complex Background (SCCB), Heavily Rearranged Unichromosomal (HRUC), Heavily Rearranged Mulichromosomal (HRMC), BFB-like). ecContext analyzes features including copy number states, transitions, overlapping SVs, foldback inversions, and dominant cycle fraction.

Additionally, we tested ecContext classifications by exploring experiments where ecDNA had been induced with known mechanisms:

- 11/11 excisional ecDNA were correctly classified as SCSB (8/8 Pradella Cre-loxP samples, 3/3 Shoshani non-chromothriptic)
- 20/23 (87%) Shoshani chromothripsis-derived ecDNA correctly classified as chromothripsis-associated

In our manuscript we highlight several new findings this method enabled about pan-cancer mechanistic patterns (Figure 3i-j):

- 36.8% (888) simple excisional, 48.4% (1,168) chromothripsis-associated, 14.8% (357) unclear origin
- Genomic location influences mechanism: chr8 FGFR1 cluster predominantly chromothripsis-associated, MYC locus balanced between mechanisms
- ecDNA size varies by formation mechanism, with simple excision-like ecDNA typically being smaller and less structurally complex than ones generated by chromothripsis-associated mechanisms.
- Tissue-specific preferences for formation mechanisms, suggesting biological factors influence ecDNA architecture

Minor comments:
- The first three paragraphs of the result section seem to be a description of AmpliconArchitect. I am sure that this can be condensed and made less technical.

Response: This was an excellent call-out by the reviewer. We have reduced the length of the first five paragraphs of the results section by ~50% (lines 88-108), moving technical details into methods and the online GUIDE.md document in the AmpliconSuite-pipeline GitHub.

- Many of the improvements made to AmpliconArchitect are not supported by data or figures.

Response: The improvements to AA span a development period of over five years and individually do not lend themselves easily to graphical representation demonstrating how each change is an improvement. We maintain a complete AmpliconArchitect changelog here. We note that based on findings shown in revised Figure 2, and the response to Reviewer #1 point 1, there is a marked improvement in ecDNA detection and resolution of its boundaries. We also feel that a deep dive into each small change could balloon out the length of the text (a simultaneous concern mentioned in the minor comment below). There are many new developments around AmpliconClassifier, ecContext, and AmpliconRepository.org that are the crux of this work.

- It is laudable that the authors have tried to make the installation of AmpliconSuite as flexible and simple as possible. However, I am not sure it needs to be described to this length in the main text. Similarly, I think the description of AmpliconRepository's user interface can be reduced.

Response: We agree with the reviewer that the installation information is better suited for GitHub, and not the manuscript itself. Consequently, we have reduced the length of that section down to just four sentences (lines 227-232). We have also trimmed as much as possible from the section describing the AmpliconRepository user interface so that it succinctly describes the organization of the site and critical functionality available there (lines 242-265).

- It would be great if the authors could add some reasoning behind the various filtering steps in AmpliconClassifier.

Response: This is also addressed in response to point 2 and 3 above by the reviewer. While the full response to those points explains in greater detail the comprehensive new work, in short, we have demonstrated that AmpliconClassifier's parameter choices are well-justified by performing systematic parameter sweeps across 12 key parameters using the expanded 175-sample FISH-validated dataset, computing F1 scores at each setting to show that the default parameters are at or near optimal performance. We have also created a comprehensive online document detailing how each of the parameters is defined and used: https://github.com/AmpliconSuite/AmpliconClassifier/blob/main/AC_config_parameters.md

- Can the authors explore which WGS coverage levels are necessary for correct classification and whether results are consistent over various downsamplings of the WGS data?

Response: We thank the reviewer for this intriguing question - and indeed it is important as the same question was also identified by Reviewer #3 (point 1). To address the effect of changing the coverage from standard (10x) downsampling to a higher mode, we randomly selected 60 high coverage cancer cell lines from CCLE with at least one focal amplification requiring that 30 were ecDNA-positive and 30 were ecDNA-negative at the default downsampling level (10x baseline coverage). We found that between the two modes of running, there were minimal differences in detection of ecDNA:

ecDNA negative (10x) → positive (30x): 3 (5.0%)
ecDNA positive (10x) → negative (30x): 1 (1.7%)

AC also predicts the number of ecDNA species present in the sample. These could vary if a structural variant joining two ecDNA into one was missed/gained between the coverage settings. When we compared the number of species identified, we found that the vast majority remained the same:

Stable (no change between ds10 and ds30): 47 (78.3%)
More species (ds30 > ds10): 10 (16.7%)
Fewer species (ds30 < ds10): 3 (5.0%)

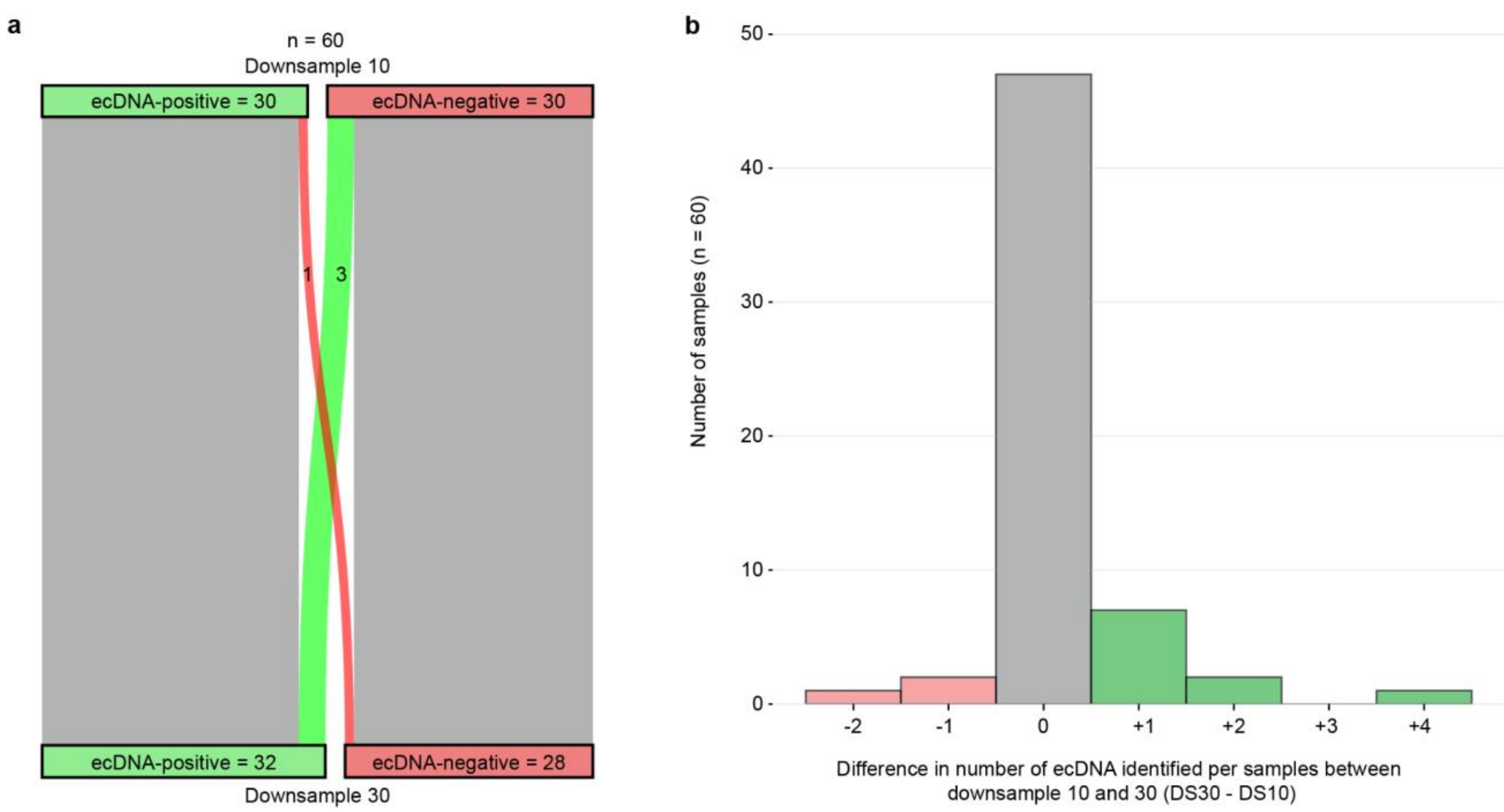

Reviewer #2:

Remarks to the Author:

In their article “AmpliconSuite: an end-to-end workflow for analyzing focal amplifications in cancer genomes”, Luebeck et al. describe a novel toolkit and analysis pipeline integrating previously published methods with novel approaches to infer amplicon structures from whole-genome sequencing in a user-friendly manner. They highlight improvements in their main method AmpliconArchitect, introduce a new method ecContext as part of their AmpliconClassifier method and demonstrate usability of their toolkit on a set of previously published cancer data. A community building website accompanies the package.

Focal amplifications are currently an important area of research in cancer genomics and as such the paper deals with a timely subject. We believe the toolkit has the potential to find wide application in the community, and we particularly applaud the authors for the community building website which will surely increase accessibility of the tool and its results. However, we do have substantial concerns regarding the manuscript in its current form, largely related to the novelty of the work and the justification of methodological approaches.

Response: We thank the reviewer for their close examination of the manuscript and related tools. We agree that this work is very timely as focal amplifications are of great importance and an exciting frontier in cancer biology. We appreciate that the reviewer agrees this work is of broad interest to the cancer community, and provides benefits to accessibility of results and methods.

Major comments

1. The authors present improvements to the overall usability and performance of the previously published tools AmpliconArchitect and AmpliconClassifier. However, the main novel methodological contribution seems to be ecContext, a rule-based tool for classifying ecDNAs based on their genetic context. While certainly interesting, ecContext feels somewhat underdeveloped. Described in a short paragraph in the main text it employs various ad-hoc chosen parameters and cutoffs to classify ecDNAs. Unfortunately, we could not find a systematic evaluation of the performance of the tool, and no thorough explanation is given about how the arbitrary thresholds and parameters were selected.

Response: The reviewer’s thoughtful and insightful assessment of our work is greatly appreciated. We have expanded the validation and explanations of the ecContext methodology to address the reviewer's concerns about systematic evaluation. ecContext introduces a novel analytical framework where none previously existed. Prior to this work, no computational tool or systematic workflow was available for classifying ecDNA structures based on their genomic context and formation mechanisms. ecContext fills a critical gap by enabling researchers to distinguish between simple excisional ecDNAs, chromothripsis-derived ecDNAs, and other complex formation patterns. However we also wish to clarify both the scope of novelty in this manuscript and the comprehensive validation we have now included for ecContext.

Beyond ecContext, this manuscript represents a substantial advancement in several other key areas. First, we also introduced a utility for the analysis of ecDNA gene co-amplifications which

reveals interesting biology of ecDNA (revised Figure 3a-e). Second, while AmpliconArchitect (AA) was previously published, yet AmpliconClassifier (AC) was reported only as a supplemental methods section in prior publications, and we have made significant improvements to both tools which improve the accuracy of ecDNA detection. The lack of a consolidated publication describing the entire workflow created significant challenges for the field as our users regularly received reviewer questions about tools' methodology and implementation. This manuscript provides the first comprehensive description of the complete workflow. We provide extensive new metaphase FISH validation data to benchmark AmpliconClassifier's performance, substantially expanding beyond prior validation efforts, and made these results publicly available for others to build from. Finally, we have developed and deployed a public web repository that makes these tools accessible to researchers without computational expertise—a significant step toward democratizing ecDNA analysis. This represents one the few truly community-editable resources of sharing focal amplification calls, and it encompasses important new analyses like our ecDNA co-amplification analysis tool described in revised Figure 3. In short, many significantly novel developments on multiple fronts.

The specific threshold values were empirically determined through iterative manual curation of ecDNA structures across CCLE, TCGA, and PCAWG datasets, where amplicon visualizations were reviewed without labels shown by multiple team members to ensure context classifications aligned with observed structural patterns. Thresholds were refined through this process to balance sensitivity for detecting chromothriptic origins while maintaining specificity for distinguishing simpler formation mechanisms.

However, to address the validation of ecContext experimentally - we have gathered examples of ecDNA generated experimentally, where the mechanism of formation is known. Two landmark studies provided this data; Shoshani et al., Nature, 2021 and Pradella et al., Nature, 2025. In the Pradella study, ecDNA was induced by engineering a circular structure from the cleaved DNA between inserted loxP sites, containing murine Myc or Mdm2. Cre recombinase excised and circularized the intervening DNA through double strand breaks, creating an oncogene-bearing megabase-scale head-to-tail ecDNA characteristic of simply-structured ecDNA which was allowed to develop in the mouse tumor. In contrast, Shoshani et al. observed spontaneous ecDNA formation in methotrexate-selected HeLa cells through chromothripsis, where chromosomal regions underwent catastrophic shattering into multiple fragments that were then reassembled into complex circular amplicons in a PARP- and DNA-PKcs-dependent manner. The Shoshani study authors documented in the figures and supplement of their study that with their genomic and cytogenetic analysis, the majority of ecDNA formations occurred through chromothripsis, however a few were marked as extrusion.

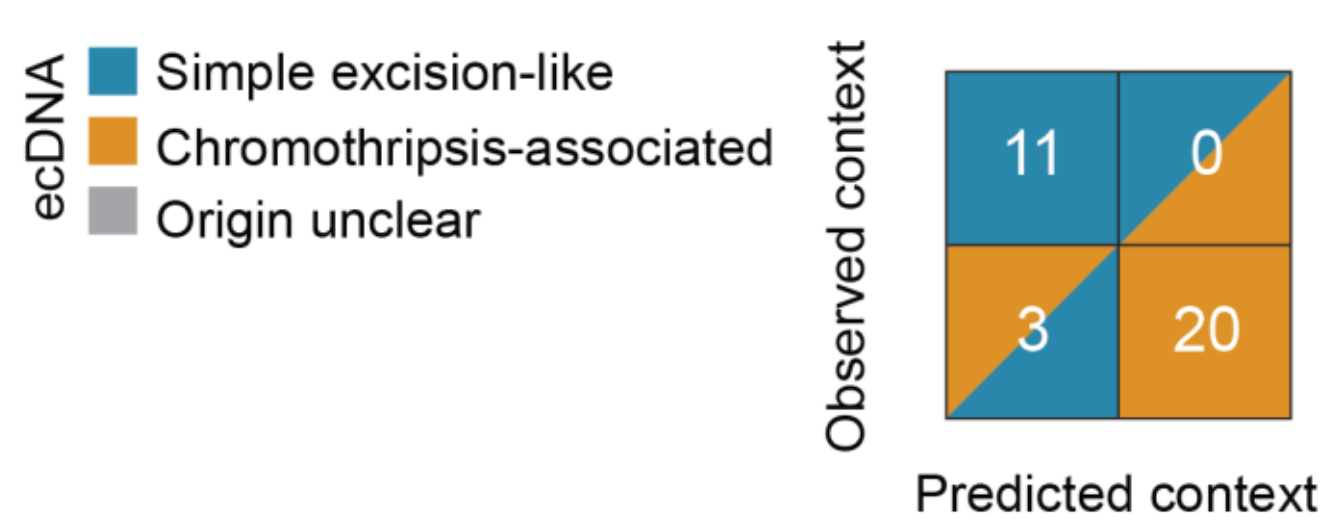

We have found that among the samples generated from non-chromothriptic mechanisms, namely excisional ecDNA formation, 11/11 (8/8 Pradella samples, 3/3 Shoshani samples) were classified as having a the “simple circular simple background” context label - which is a non-chromothriptic category. Among samples from the Shoshani study with ecDNA capturing segments of chr5 20/23 (87%) were given context labels associated with chromothripsis ("heavily rearranged unichromosomal", "simple circular complex background"). These results are available in revised Figure 3h and in Supplementary Table 8.

Figure 3, which now better reflects the novel enhancements to AmpliconSuite - namely the ecDNA co-amplification and ecDNA context work is reproduced below.

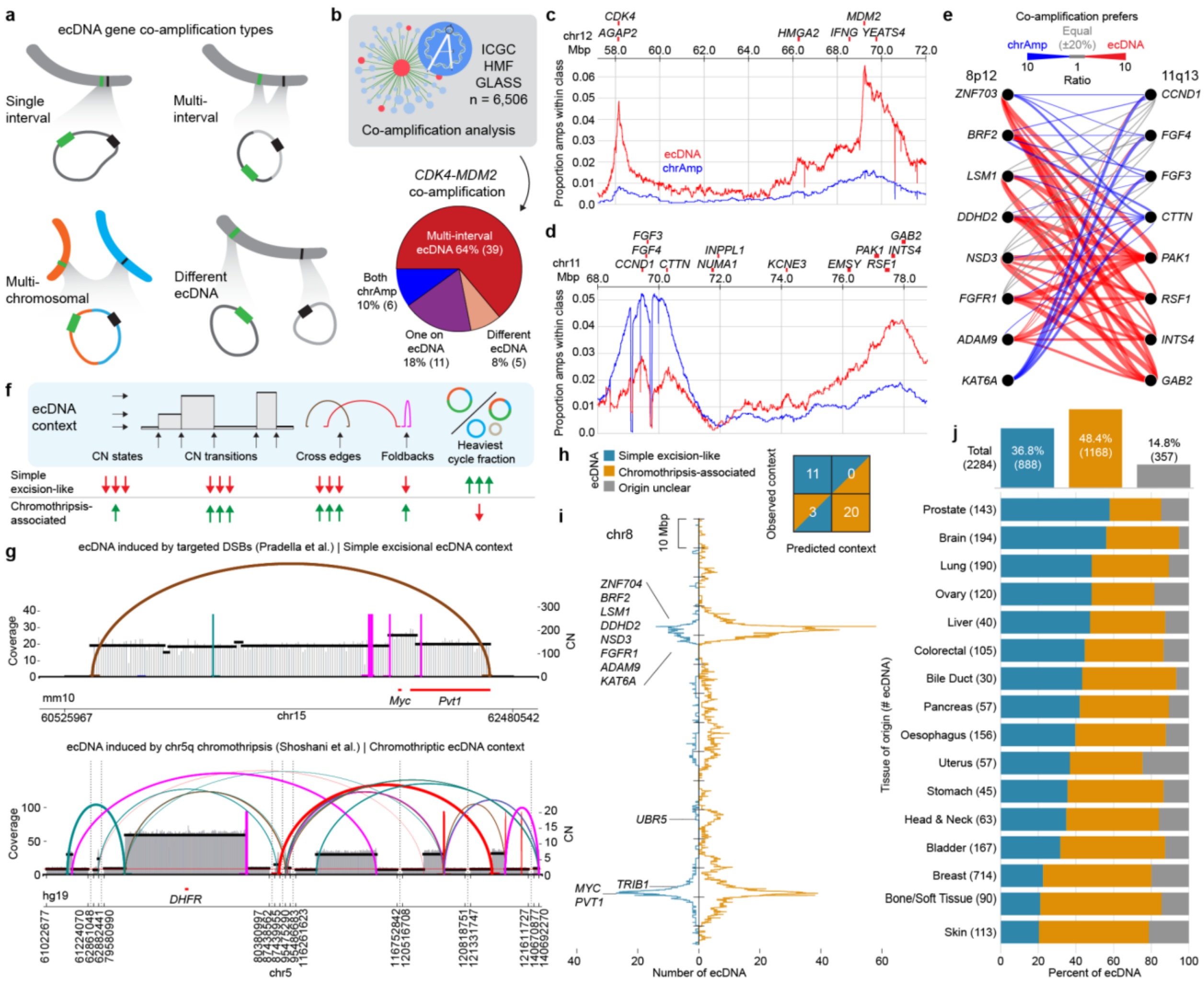

2. Additionally, the relationship between the features computed by ecContext (enumerated as (i) to (vi) in the Methods section) and the resulting ecDNA classes (enumerated as 1. to 5.) is unclear. A tabular description or decision tree of all rules and how they are applied together with the precise thresholds and their motivation (see comment 1) should be given.

Response: The reviewer raises an excellent point about the clarity of the ecContext method description. We agree that the ecContext method could use a visual description. We have updated Supplementary Figure 8 to include panel e, reproduced below, showing a decision tree of the ecContext method. Revised Figure 3f (reproduced in response to point #1 above) also shows how the metrics (i to vi) influence the discovery of chromothripsis-associated ecDNA. We have also updated the methods section (lines 770-784) to clarify how each of the scores computed to determine the ecDNA context is used.

In brief**,** ecContext uses a hierarchical decision tree to classify ecDNA formation mechanisms. The method computes six structural complexity metrics from the ecDNA regions: (i) cycle fraction, which is the proportion of ecDNA explained by the heaviest circular path, (ii) number of copy number transitions, (iii) number of copy number states, (iv) number of crossing SV edges, (v) number of foldback inversions, and (vi) whether the ecDNA is unichromosomal or multichromosomal.

Classifications are evaluated in priority order, with the first matching condition determining the result:

1. **BFB-like** requires abundant foldbacks (>3, comprising >10% of SV edges), multiple CN states (>2), and unichromosomal content, reflecting iterative breakage-fusion-bridge cycles.
2. **Two-foldback** requires foldbacks near both ecDNA ends with otherwise low structural complexity (≤6 CN states, T/N<4 or <4 cross edges), consistent with replication fork regression.
3. **Heavily rearranged** requires high structural complexity (T/N≥4 or ≥4 cross edges, or >6 CN states), subdivided into unichromosomal (HRUC) vs multichromosomal (HRMC), capturing chromothripsis-associated origins.
4. **Simple circular** requires either ≥15% cycle fraction or ≥30% overlap with a single everted edge cycle (head-to-tail cycle), with low structural complexity (≤3 cross edges, T/N<4, ≤6 CN states, unichromosomal). These are further classified by assessing amplicon-wide complexity: simple circular complex background (SCCB) if the broader genomic region analyzed in the amplicon shows high complexity (T/N≥4 or ≥4 cross edges or >6 CN states), or simple circular simple background (SCSB) otherwise.
5. **Unknown** captures ecDNA not matching any defined pattern.

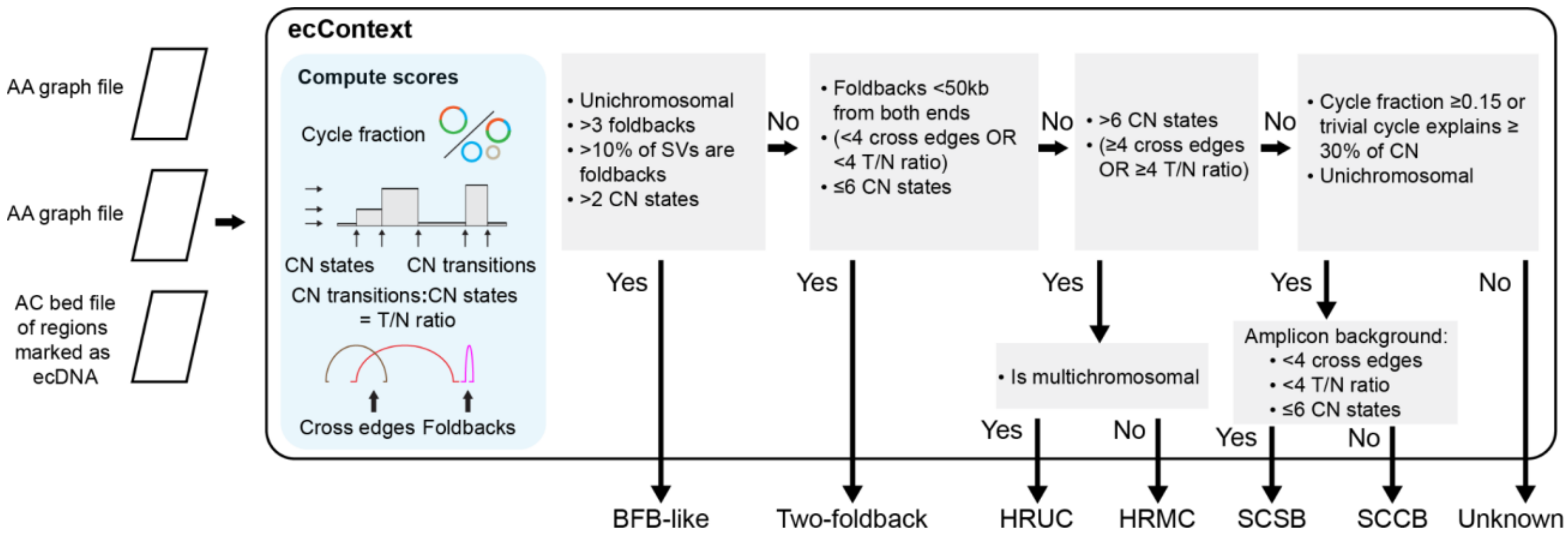

3. The authors report on improvements of detection of focal amplifications including ecDNA, but only give sensitivity values for ecDNA detection. Can the authors provide a thorough evaluation of the detection and classification performances of all parts of AmpliconSuite including in particular accuracy measures for the classification of other focal amplifications outside ecDNAs?

Response: We thank the reviewer for this important comment and note that this comparison against non-ecDNA amplifications was also a request highlighted by Reviewer #1 in their point 2.

As was mentioned in that response, we wish to highlight that the analysis of ecDNA or other types of focal amplifications at a sequence level is a relatively new scientific field. There is very little gold standard data providing fully resolved ecDNA or BFB sequences. Even imaging-based approaches for ecDNA detection based on FISH are scarce, scattered across many different sources and do not represent a large enough dataset for robust machine learning. Our set of 175 metaphase FISH-stained gene probes with contemporaneous WGS is intended as a resource for the research community and we believe will enable the beginning of larger-scale truth datasets.

Of focal amplifications called by AC as "Linear" (chromosomal amps with minimal rearrangements) is that of the 35/37 focal amps called as Linear by AC and probed with FISH genes appeared as clear HSRs, while the other two were indeterminate for DM (potential rare ecDNA or FISH artifact). Whether a chromosomal amplification appears on its native chromosome (native HSR) or on a different chromosome (non-native HSR) is also informative about the correctness of a focal amplification prediction. For instance the BFB amplification process acts on a chromosome arm, creating chains of inverted duplications, and we should expect the FISH to show an amplified BFB gene co-localize with the native chromosome marker. For BFBs, we noted that 8/9 BFBs predicted by AC with a probed amplicon gene and chromosome marker appeared as native HSRs.

However, to get a more definitive and larger-scale understanding of the correctness of the BFB predictions at a sequence level, we examined the performance of AC against results from a large-scale optical genome mapping study (Dehkordi et al., Nature Communications, 2025) that

identified breakage-fusion-bridge (BFB) focal amplifications, where matched WGS data was also available.

Using AC v1.5.1, we compared WGS-based predictions to OM2BFB calls as truth (revised Figure 2g), we found BFB detection using AC had: Sensitivity (Recall): 0.71, Specificity: 0.95, Precision: 0.88

	OM2BFB: BFB	OM2BFB: Not BFB
AC: BFB	22	3
AC: Not BFB	9	59

4. While ecDNAs are relatively easy to detect using FISH allowing generation of an experimental gold standard, this seems to be more difficult for other types of amplifications. The choice of a benchmark dataset needs to be discussed, the used cell lines were also used for the AmpliconArchitect paper and it is unclear how accurate these reference calls are. Are other novel datasets available to corroborate the findings from the cell lines?

Response: We fully agree that additional data modalities will be important for validating non-ecDNA predictions. Notably, as described in the response to point #3 above, we benchmarked BFB detection on 93 focal amplifications where both optical genome mapping and WGS data were available, providing a strong orthogonal validation.

We agreed that the experimental gold standard should be expanded significantly in order to demonstrate the tools performance on a wider variety of focal amplifications. As described in response to reviewer 1, point #1, to address this, we have expanded our set of cell lines with cytogenetically validated ecDNA status. Previously, we relied on a set of 66 total metaphase FISH-probed genes across 43 different cell lines as a truth set for comparing our classification heuristics. We performed **extensive** additional validation experiments, expanding this dataset by >100 additional probes to a total of 175 metaphase FISH-probed genes across 72 different cell lines or models, by incorporating new data (85 of the probe genes) with prior results from five different studies.

Importantly, this new data set contains a close balance of different cytogenetic amplification types; 56 ecDNA (DM), 3 indeterminate for ecDNA, 52 non-native HSR (homogeneously staining region) - where the amplified gene was located on a different centromere than its corresponding centromeric probe, 39 native HSR, and 25 unknown HSR types - where the centromere was not concurrently probed with the gene. We believe that by releasing this dataset with the paper, the broader community will benefit, and it will improve others' methods development efforts for focal amplification detection.

An important caveat, however, of using cell lines for ecDNA analysis is that the re-integration of ecDNA into chromosomal DNA is a common phenomenon in cancer cell lines, and may occur spontaneously (Alitalo et al., PNAS, 1983; PMID: 6300869) or be induced by changes to selective pressures (Nathanson et al., Science 2014; Song et al., Cancer Discovery, 2022). Prior analysis of HSR integration points is limited, but based on existing findings and a null model of random re-integration, a non-native integration location is expected (Luebeck et al., Nature Communications, 2020, Song et al., Cancer Discovery, 2022).

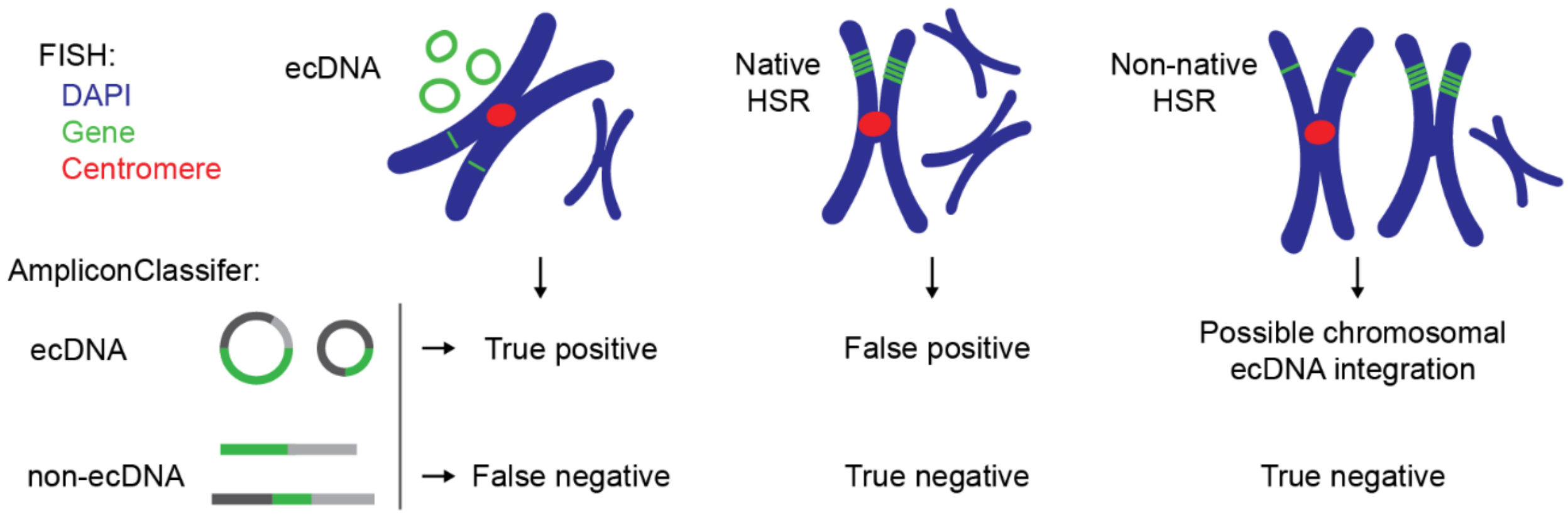

When ecDNA was identified by WGS, and a non-native HSR was observed in the amplicon's FISH data, we marked these cases as neutral in the calculation of performance metrics. Such ecDNA-derived amplifications still carry the signatures of ecDNA in their SV "fossil records". In patient tumor samples, on the other hand, the landscape of selective pressures is heterogeneous across the tumor and over time. Thus, even if a significant fraction of cells were converted to re-integrated ecDNA and appeared as HSR, we argue that some ecDNA cells would likely remain and could be re-selected when selective pressures changed again (Nathanson et al., Science, 2014). Predicting ecDNA for cases where an ecDNA-derived HSR appears dominant should not necessarily be marked as false positive in the same way an amplification with no ecDNA involvement should be penalized.

With this in mind, on the panel of 175 amplifications with ecDNA status validated by metaphase FISH we then applied three versions of AmpliconClassifier; AC 2020 (Kim et al., Nature Genetics, 2020), AC v0.4.13 (Luebeck et al., Nature, 2023), and the most recent version AC v1.5.1. The latest version showed the best performance, highlighting the improvements to the method over time.

Metric	AC 2020	AC v0.4.13	AC v1.5.1
Sensitivity	92.9%	92.7%	94.6%
Specificity	85.2%	93.1%	94.2%
Precision	81.3%	89.5%	91.4%

We have incorporated these findings into a heavily revised Figure 2 (below) which illustrates the methodology for creating the truth set (Fig. 2c-e), and a visual representation of performance improvements to AmpliconClassifier (Fig. 2f) and the placed this information in Supplementary Table 2.

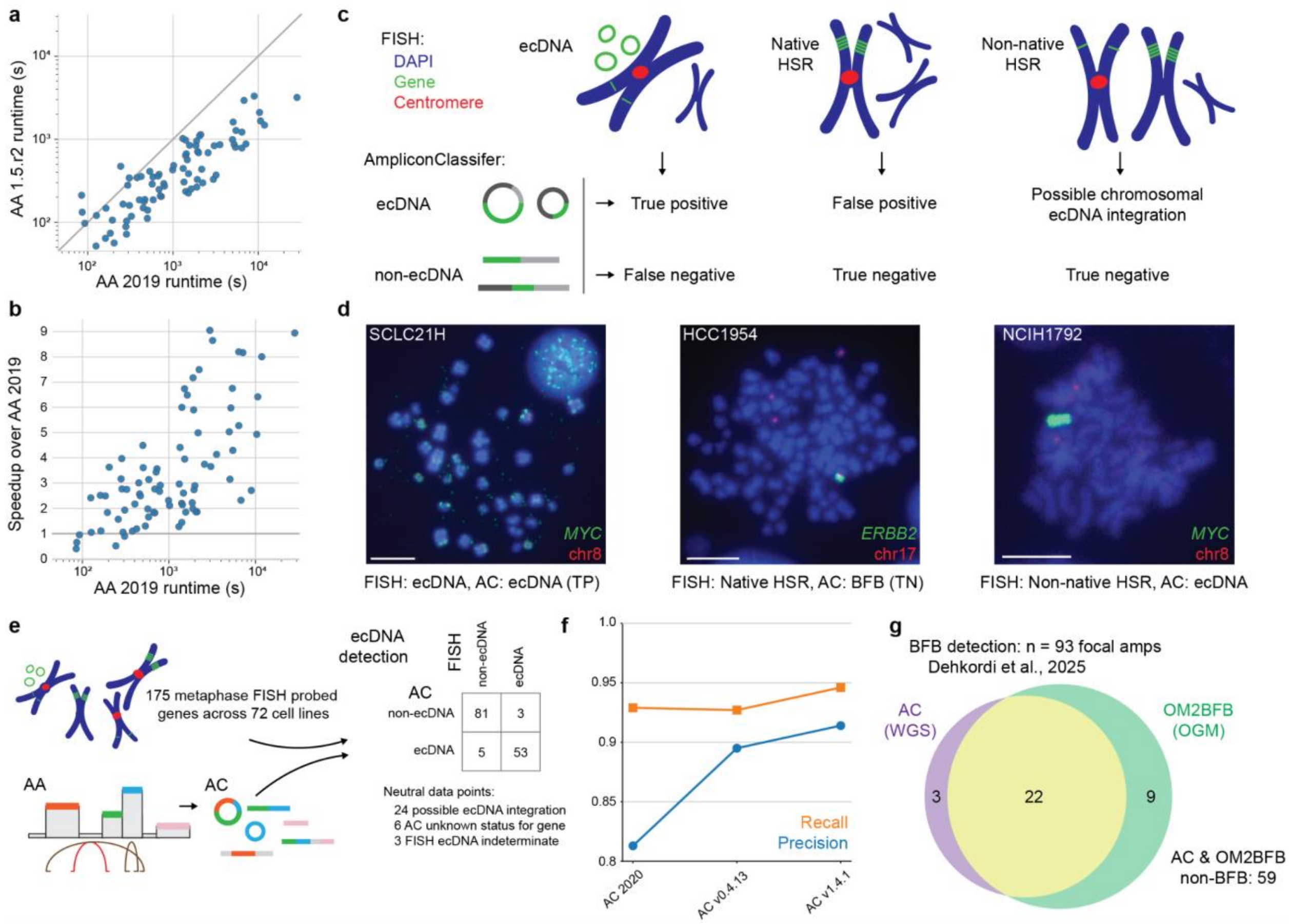

5. As a side note, the Kim et al. paper states that 44 cell lines were used, the authors here use only 43 cell lines. What happened to that one cell line?

Response: We thank the reviewer for this astute observation. Cell line MDAMB231 was included in Kim et al., Nature Genetics, 2020 as an ecDNA-negative case. However it is not included in our comparison. MDAMB231 contained no focal amplifications in FISH or WGS data, and our benchmarking in Figure 2 is restricted only to samples which do show evidence of focal amplification. Indeed, with the latest version of AmpliconSuite-pipeline this sample still remained without any predicted focal amplifications. Thus it had no natural place in the categorizations of focal amplifications and inclusion of such cases with no evidence of any focal amplification risks overinflating the true negative rate.

6. Beyond additional test datasets, sophisticated simulations could reveal the improved performance of AmpliconSuite over prior implementations and elucidate sensitivity, specificity and classification accuracy values more systematically.

Response: We agree that the reviewer has suggested a very important additional analysis. To demonstrate the ability to predict ecDNA in simulated data. We utilized simulated ecDNA structures derived from chromothriptic and episomal mechanisms from the simulated amplicons, simulated using our tool ecSimulator (https://github.com/AmpliconSuite/ecSimulator. These 86 simulated cases were created from simulating ecDNA structures with rearrangements, followed by simulation of WGS sequencing data from these structures mixed with background reads at ~10x coverage using the Mason2 WGS simulator. The set of sequencing data was part of a previously dataset for benchmarking the performance of nanopore-based breakpoint detection with CoRAL (Zhu, Jones et al., Genome Research, 2024) vs. AA, but here we used it for the purposes of benchmarking ecDNA detection.

To illustrate the high complexity and realistic nature of the simulated data, we provide examples of real ecDNA with generative context labeled found in cell lines (left column) and simulated examples (right column).

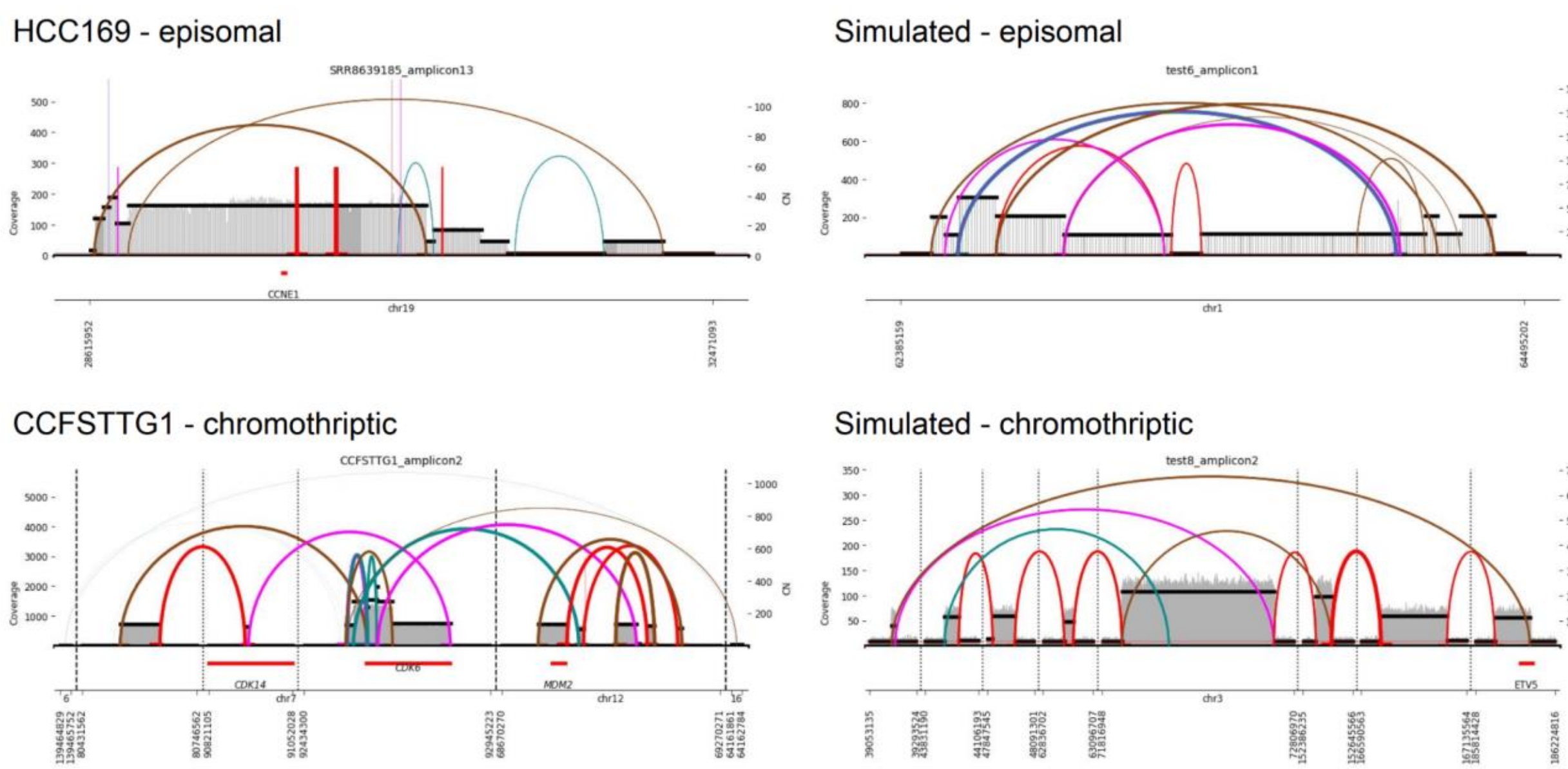

Using the newest AC v1.5.1, this gave

ecDNA simulation type	Simulated cases	Recovered (AC v1.5.1)	Fraction recovered
chromothripsis	44	39	0.89
excisional	42	35	0.83

However, using the older AC 2020 version (Kim et al., Nature Genetics, 2020) for classification on this same dataset, we found the newer version of AC provided a marked improvement in ecDNA detection.

ecDNA simulation type	Simulated	Recovered (AC 2020)	Fraction recovered
chromothripsis	44	20	0.46
excisional	42	26	0.62

Compared to the FISH-validated cell line benchmarking, the simulated data showed marginally lower ecDNA detection sensitivity. This difference likely reflects the simulation design: while the largest unmappable genomic regions were excluded (e.g. centromeres, telomeres), the simulations sampled uniformly across the remaining genome, rather than enriching for oncogene-rich regions like real ecDNA. Real ecDNAs preferentially harbor mappable, oncogene-dense sequences, while the simulations included a greater proportion of low-complexity gene deserts. These regions may have impaired structural variant detection with short reads, creating a more challenging landscape for SV identification than typically encountered in authentic cell line data.

We also evaluated the sensitivity of ecDNA detection across varying tumor purity levels by performing *in silico* dilutions of cancer cell line sequencing data with normal reads from NA12878. Starting with 9 ecDNA-rich cancer cell lines with 34 different ecDNA species predicted at baseline, we created dilution series at 28 different purity levels ranging from 4.8% to 95.2% tumor content, then performed ecDNA detection using AmpliconSuite-pipeline on each diluted sample (revised Methods lines 631-651). An ecDNA from the baseline sample was considered successfully re-identified if the original genomic coordinates showed overlap with ecDNA coordinates detected in the dilute samples. We also computed a simple theoretical detection limit which was defined as the combination of purity and ecDNA + background copy number which would create an amplification event with 4.5 total copies or greater (lines 644-651).

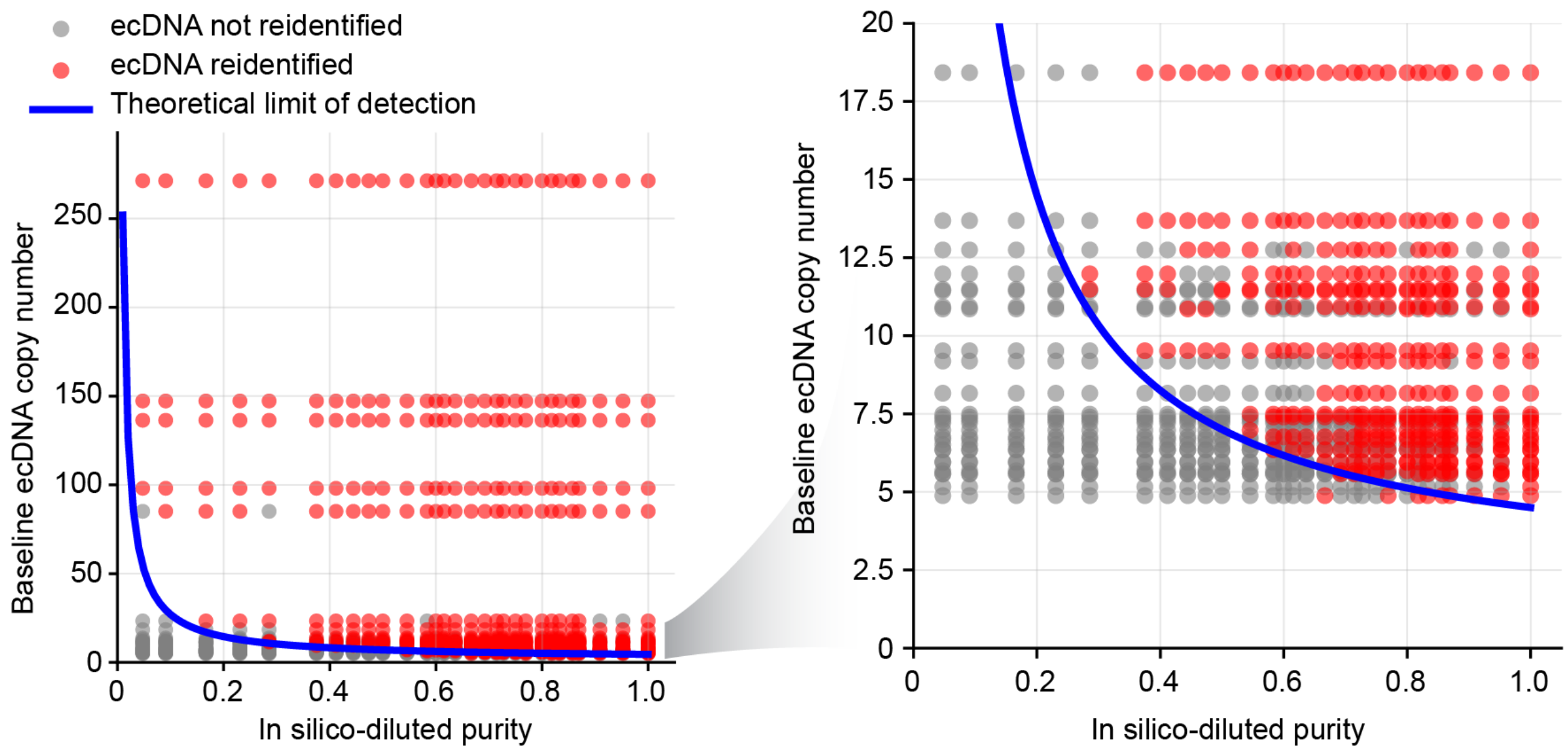

The results demonstrate that detection sensitivity strongly correlates with both tumor purity and ecDNA copy number. Detection rates dropped significantly for lower-copy ecDNAs and lower tumor purities, following a characteristic detection threshold curve consistent with the theoretical limit where measured copy number approaches the diploid background. When compared against the theoretical detection curve, 79.5% of theoretically-detectable ecDNAs (dilution-adjusted CN ≥ 4.5) were recovered in the diluted samples.

We have included these simulation findings in the Results section (lines 205-211) and as revised Supplementary Figure 6.

7. The authors explored the complexity and genomic span of focal amplifications. They report lower complexity and higher variance in genomic span in ecDNA compared to BFB. Can the authors speculate on why this could be observed?

Response: We appreciate this thought-provoking question. The observed patterns likely reflect fundamental mechanistic differences between ecDNA and BFB formation.

Lower complexity in ecDNA: The distribution of complexity scores for ecDNA is lowered by simple excisional events, where a single circular fragment is excised from the chromosome with minimal rearrangement. These "clean" excisions, which represent a substantial fraction of ecDNA formation events (Figure 3j) have inherently low structural complexity, pulling down the average complexity score. In contrast, BFB cycles always involve repeated breakage-fusion-bridge events that progressively accumulate inversions and duplications, rarely if ever, producing simple structures.

Higher variance in genomic span for ecDNA: ecDNA can form through multiple distinct mechanisms (chromothripsis, excision, etc.). Regardless, ecDNA formation requires only two

double-strand breaks, which could occur essentially anywhere in the genome from a few kilobases to multiple megabases apart depending on the initiating event. This mechanistic flexibility naturally produces high variance in circle size. Conversely, BFB amplifications are constrained by a mechanism which involves entire chromosome arms: they initiate from telomere loss or chromosome arm fusion and propagate through repeated bridge-breakage cycles which are ultimately constrained to the chromosome arm. This process typically amplifies regions extending from the initial fusion point, producing a product that is more constrained to the macro-scale, and less likely to be limited to a small localized region.

We speculate that ecDNA's mechanistic diversity generates both the simplest structures (excision events) and the greatest size variability, while BFB's iterative process yields consistently complex structures of more uniform scale. In our Results section we performed a new statistical analysis of ecDNA against all chromosomal focal amps in the expanded pan-cancer cohort, stating (lines 379-382): "One important observation we found was that compared to chrAmps, ecDNA showed a significantly greater variance in amount of reference genome it captured across the distribution of focal amplification sizes (ecDNA standard deviation 3.22 Mbp, chrAmp standard deviation 2.23 Mbp, $P < 1x10^{-16}$, Bartlett's test). The high variance in ecDNA size may be ascribed to the wide variety of mechanisms that form it." The subsequent section which follows, on ecDNA context and how the simple-excisional ecDNA tend to be smaller, further underscores this finding.

8. The authors state that biologically unrelated samples should not share exact genomic boundaries and implement a similarity score-based filter to control for false-positive focal amplifications. How might specific phenomena that predispose the genome to break at specific positions (such as fragile sites) influence this assumption? Can the authors give an example how this filter improves the analysis and elaborate on when to use the filter?

Response: In the calculation of the similarity score, for two SVs to be considered the same between different samples, we require that the total difference between both SV endpoints be no more than 250bp apart. To illustrate this, we performed an analysis of ecDNA SV endpoints in the unfiltered version of the CCLE dataset. We asked if we could detect any conserved ecDNA-associated SVs between the 329 samples in the dataset located on the q arm of chr8, which contains MYC/PVT1 and was the most frequent source of ecDNA in that dataset. This region is particularly likely to show recurrent SVs as genomic rearrangements around MYC/PVT1 are highly common in cancer genomes and recurrently create MYC-PVT1 fusions, particularly mediated by ecDNA (Yi, Zhang et al., Cell, 2026; PMID: 41506267). After filtering indel-type SVs smaller than 10kbp, we found there were no conserved chr8q ecDNA SVs where different SV's endpoints matched within 1000bp in that dataset. When requiring the endpoints to be less than 10kbp apart, we began to observe pairs of SVs flagged as having shared endpoints, namely near the MYC locus, suggestive of oncogenomic alterations of independent origins, having nearby, but inexact conservation. These findings highlight that filtering based on extremely tight (250bp) conservation of SV endpoints is unlikely to remove real, recurrent phenomena.

To illustrate how this filter improves results, we performed a case study on the filtering of focal amps based on similarity score using the CCLE dataset (GRCh38). In examining the similarity scores of the amplicons in the cohort, we observed the top similarity score was for two amplicons which shared an ecDNA classification constituted by a head-to-tail genome cycle of a segment of ~61kbp derived from chr5:32.10Mbp-32.17Mbp. Importantly, the SV endpoints were shared (distance < 250bp). Through similarity score-based filtering, we discovered this particular event occurred in 3 of 329 samples analyzed. This example is the subject of revised Supplementary Figure 3, shown below.

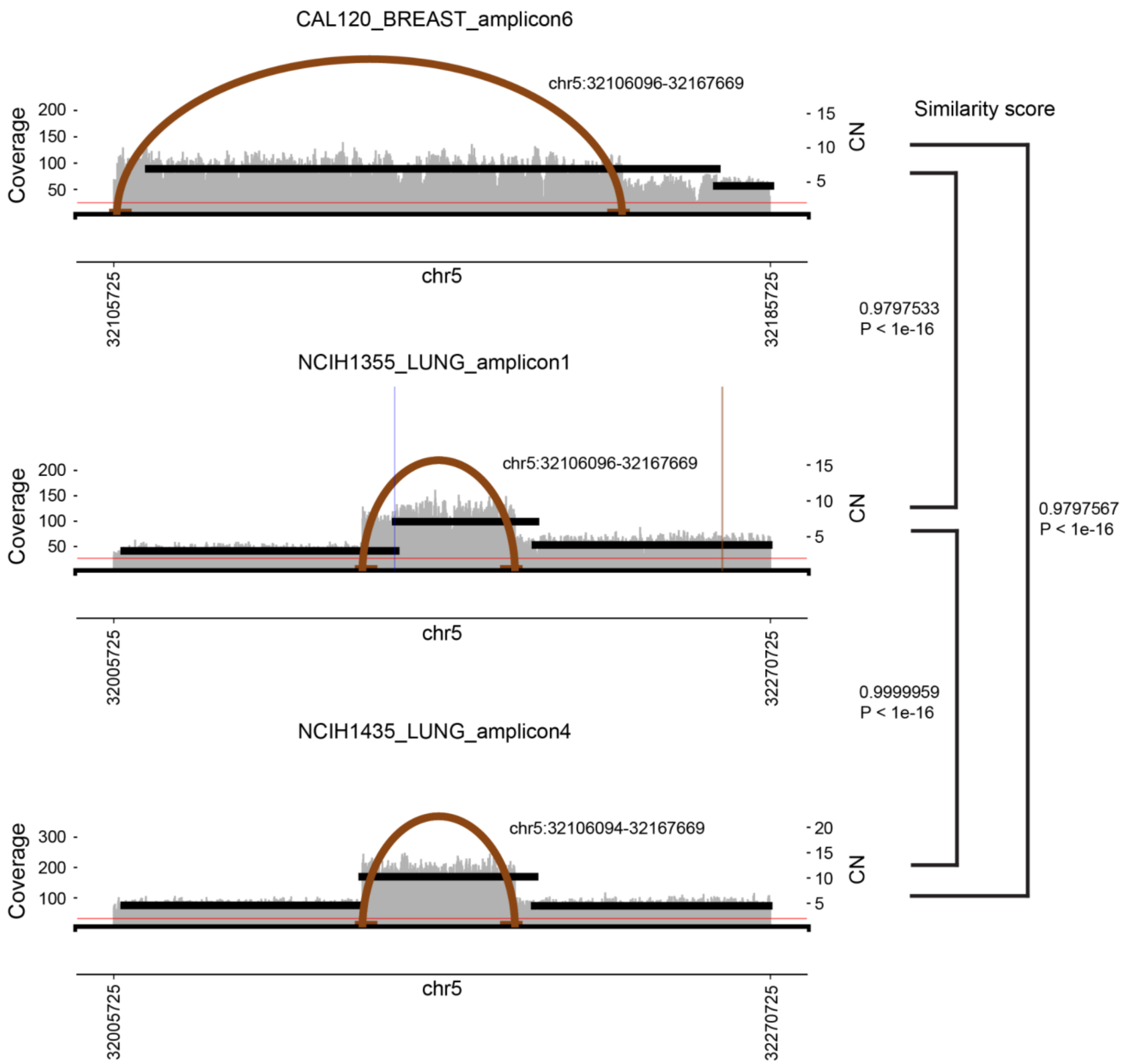

Examination of the SV forming this cycle against the gnomAD-v4.1 SV database revealed this event to be consistent with a rare ~61kbp multi-copy duplication which is found in 2.2% of the human population (link below, gnomAD-v4.1 DUP_CHR5_C02C1ED4, https://gnomad.broadinstitute.org/variant/DUP_CHR5_C02C1ED4?dataset=gnomad_sv_r4

). While the AmpliconArchitect data repo contains annotations on repetitive elements, large tandem duplications and conserved high signal regions - germline amplification events which are highly infrequent in the population are likely missing and thus not filtered from AA analysis. Thus, the detection of ecDNA can be improved, particularly in large-scale analyses through similarity-based filtering. This finding is included in the Results section on lines 118-127.

Minor comments:

- l.275: Caner Cell Line → Cancer Cell Line
- l.135: ‘may arise due from’ → ‘may arise from’ or ‘may arise due to’
- l.102: the abbreviation SV for structural variants is defined here, but structural variant appears earlier (l.59)
- l.40,58,79: multiple definitions of the abbreviation AA for AmpliconArchitect; similar for AC, please check

Response: We thank the reviewer for their close reading of the manuscript. We have addressed these typos. Because the abstract may be self-contained in some reproductions of the work, we chose to define acronyms from the abstract again in the main text. However, we will check with editorial staff to ensure this is correct.

Remarks on code availability:
We reviewed the code and found it well documented, but did not run it ourselves.

Response: We thank the reviewer for their examination of our code. Our documentation provides a small test case for users who wish to try out the method, using a small BAM file ~2GB obtainable from SRA.

Reviewer #3:

Remarks to the Author:
Jens Luebeck and colleagues reported the development of an all-in-one bioinformatic pipeline AmpliconSuite for analyzing focal amplifications in cancer genomes. The AmpliconSuite pipeline developed by the study has made significant efforts to improve and integrate the analytic packages for analyzing, visualizing, and interpreting focal amplifications in pair-end WGS cancer genomes. Although many packages were integrated into the AmpliconSuite-pipeline with their original but well-developed codes, significant amounts of modifications and improvements have been introduced into the AA, AC, and ecContext.

The AmpliconSuite could not be regarded as a completely novel method. However, it is a significantly improved and streamlined tried-and-tested technique for focal amplification analysis. The research team has significantly improved the sensitivity and performance of the AmpliconArchitect (AA) and AC. As a validation of the codes developed by the study, we have performed back-to-back comparison of the older version of AA and the AmpliconSuite pipeline using in-house generated WGS data from UBC. In line with the sensitivity and performance reported by the study, the AmpliconSuite-pipeline is significantly faster, smoother, and more informative than previous AA. Since there are rapidly increasing interests in understanding the roles and translatability of ecDNAs in cancer diagnosis and treatment, the AmpliconSuite will become one of the most popular and broadly used tool kits for studying focal amplification and ecDNAs in cancer genomes.

Furthermore, this study has developed an interoperable database ampliconrepository for the scientific community to share, re-use, and analyze focal amplification data. Although the ampliconrepository will benefit from some further modifications (comments below), it is a great example of the foundational FAIR (Findability, Accessibility, Interoperability, and Reusability) principles.

To demonstrate the broad applicability of the AmpliconSuite-pipeline, the study has analyzed over 2500 WGS genomes from tumors and cancer cell lines. These analyzed data have been shared and illustrated in their database AmpliconRepository.org. A conceptionally interesting and important point introduced by the study is ecContext for categorizing the ecDNAs by their generative mechanism. Although some of these categorizations remain discussing, this is an important step toward better understanding of the ecDNA functions.

Overall, the AmpliconSuite is a significant improvement to the tried-and-tested AmpliconArchitect bioinformatic tool. This is a timely all-in-one and end-to-end workflow to meet the great needs of analyzing cancer WGS data. The nature of code-free (GenePattern and nextflow) and end-to-end makes the method to be easily adapted by the broad scientific community, even with limited bioinformatic skills. Although the manuscript, methods, and figures have already been well prepared, the study and manuscript will benefit from addressing a few important points.

Response: We thank the reviewer for their thorough and insightful review of our work. We further appreciate the acknowledgment of our efforts to enhance and integrate the modules in this

workflow, particularly in improving the sensitivity and performance of the methods. We look forward to addressing this valuable feedback to further enhance the clarity and impact of the work.

1.1 The AmpliconSuite-Pipeline

We have tested the AmpliconSuite pipeline and codes with in-house generated WGS data from human tumors. The performance of the pipeline is excellent in term of speed and operability. The amplicon detection results are consistent between the old and new version, but the contexts provided have been significantly improved in the new version as compared to the older one. However, it is unclear about the effect of WGS coverage on the AmpliconSuite (or AA) sensitivity. The previous AA has suggested 5-10X coverage. In the current study, only samples with at least 10X coverage and 0.4 tumor purity had been included for analysis. Although the authors have commented that the pipeline does not include the QC assessment of the WGS dataset, assigning certain degree of QC cutoffs will benefit the subsequent projects and results inclusion in the AmpliconRepository.

Response: We thank the reviewer for their excellent question and suggestions. To the reviewer's first comments about QC assessments and the effect of coverage and purity, we performed two new analyses.

Regarding the effects of coverage, we note that a similar question was a question asked by Reviewer #1 related to the effect of downsampling. Importantly, the original version of AA was developed around a validation set of samples that were conducted at ~1x coverage (Deshpande et al., Nature Communications, 2019). To address the effect of changing the coverage from standard (10x) downsampling to a higher mode, we randomly selected 60 high coverage cancer cell lines with at least one focal amplification from CCLE requiring that 30 were ecDNA-positive and 30 were ecDNA-negative at the default downsampling level (10x baseline coverage). We found that between the two modes of running, there were minimal differences in detection of ecDNA:

ecDNA negative (10x) → positive (30x): 3 (5.0%)
ecDNA positive (10x) → negative (30x): 1 (1.7%)

AC also predicts the number of ecDNA species present in the sample. These could vary if a structural variant joining two ecDNA into one was missed/gained between the coverage settings. When we compared the number of species identified, we found that the vast majority remained the same:

Stable (no change between ds10 and ds30): 47 (78.3%)
More species (ds30 > ds10): 10 (16.7%)
Fewer species (ds30 < ds10): 3 (5.0%)

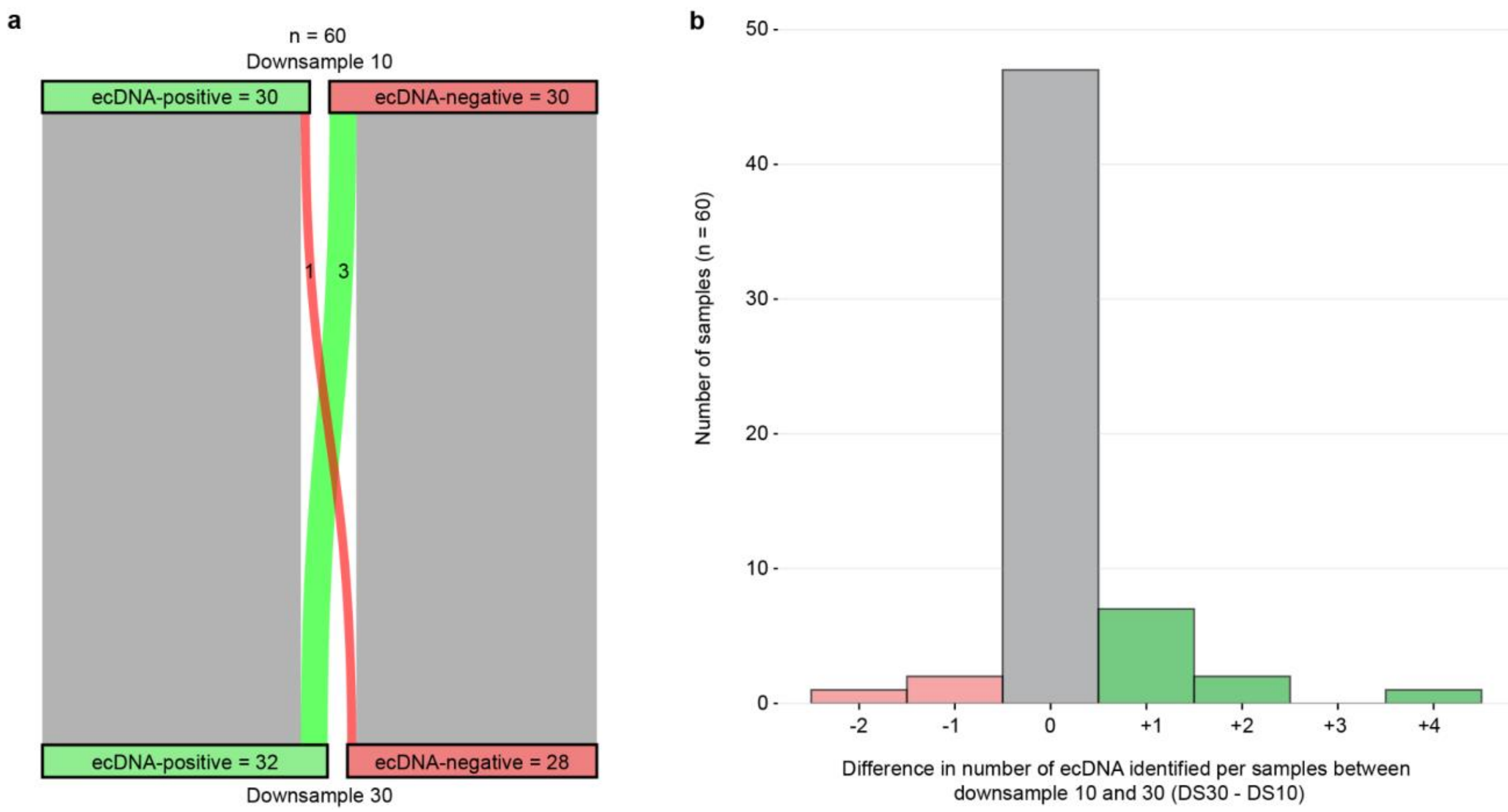

One important caveat of increasing coverage was that in this example, 17/60 samples increased runtime by more than an hour. This disproportionately affected the cases that were already longest to run (most complex amplicons). For instance SKBR3 went from 8 to 31 hours, and BT474 went from 5 to 14 hours, highlighting the computational challenges with increasing coverage to high levels.

Regarding the effect of purity, we noted Reviewer #2 also touched on the need for simulations to demonstrate sensitivity of the method, and the suggestion raised here combined naturally with the need to find the limits of detection given sample purity. We evaluated the sensitivity of ecDNA detection across varying tumor purity levels by performing diluting cancer cell line data with normal reads from NA12878. Starting with 9 ecDNA-rich cancer cell lines with 34 different ecDNA species predicted at baseline, we created dilution series at 28 different purity levels ranging from 4.8% to 95.2% tumor content, then performed ecDNA detection using AmpliconArchitect on each diluted sample against the GRCh38 reference genome (revised Methods lines 631-651). An ecDNA from the baseline sample was considered successfully re-identified if its genomic coordinates showed overlap with detected ecDNAs in the diluted samples. We also computed a theoretical detection limit which was defined as the combination of purity and ecDNA + background copy number which would create an amplification event with 4.5 total copies or greater (Methods lines 644-651).

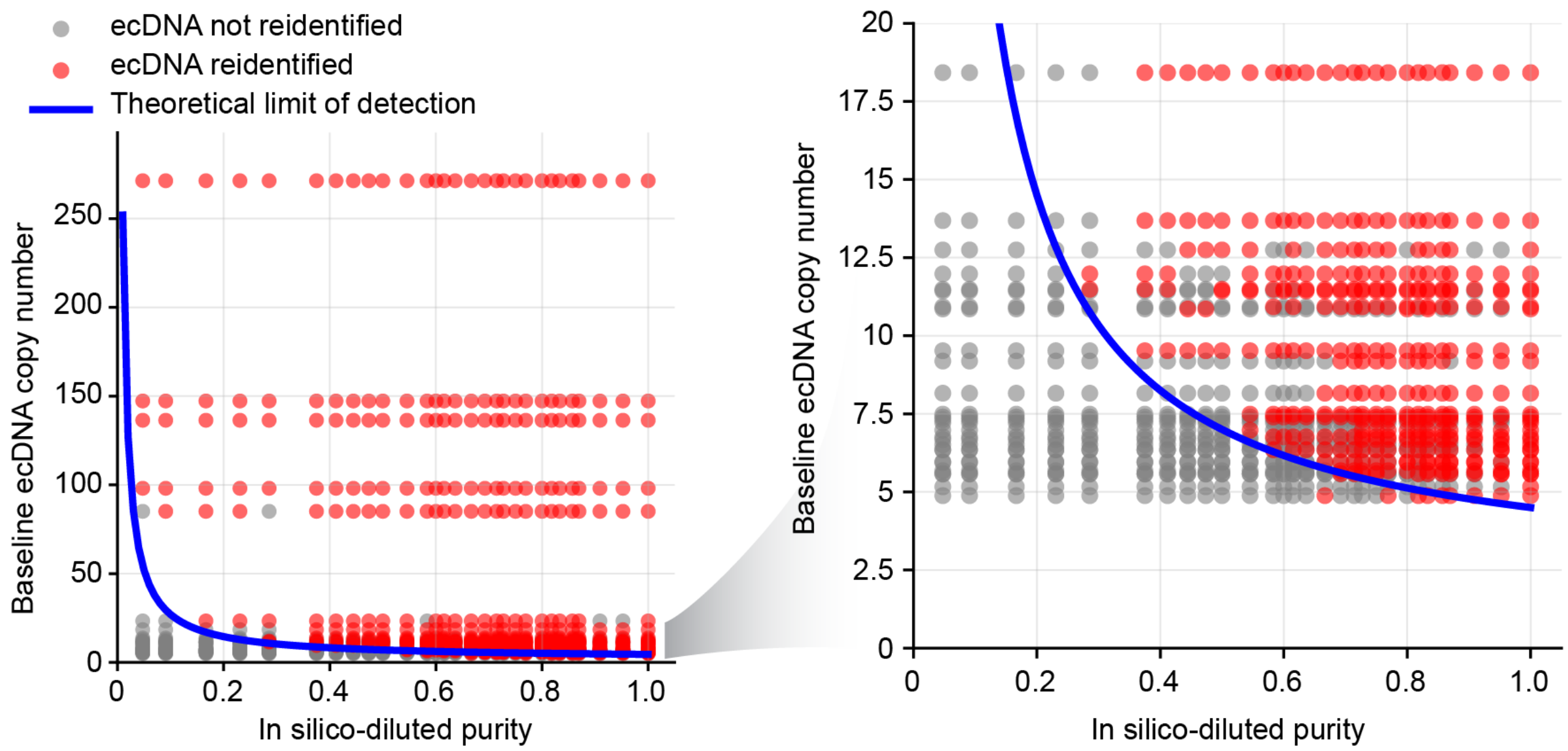

The results demonstrate that detection sensitivity strongly correlates with both tumor purity and ecDNA copy number. Detection rates dropped significantly for lower-copy ecDNAs and lower tumor purities, following a characteristic detection threshold curve consistent with the theoretical limit where measured copy number approaches the diploid background. When compared against the theoretical detection curve, 79.5% of theoretically-detectable ecDNAs (dilution-adjusted CN ≥ 4.5) were recovered in the diluted samples. We included this in the Results section (lines 205-211) and as revised Supplementary Figure 6.

A very good point is made by the reviewer that providing guidance on QC cutoffs may benefit users. One important QC parameter, which controls the minimum distance between read pairs to call an indel-like SV is automatically determined by AmpliconSuite-pipeline based on insert size distribution in the sequencing data. However other metrics, like QC based on purity, are difficult to automate, as optimal QC thresholds depend heavily on the specific research question and sample characteristics. For instance, FFPE samples often have lower quality metrics than fresh-frozen tissue but can still yield valuable ecDNA calls, while liquid biopsy samples may have very low tumor fractions that would fail standard purity cutoffs despite being clinically relevant. Additionally, coverage requirements vary based on the expected ecDNA copy number - high-copy ecDNAs can be reliably detected at lower coverage, while complex rearrangements benefit from deeper sequencing. Rather than imposing rigid cutoffs that might exclude valuable samples, we hope the analyses above (which we will make readily available in our tool documentation and in the manuscript) will allow users to make informed decisions based on their specific experimental context. We have added documentation to our Github GUIDE.md file, documenting the relationship between coverage, purity, and detection sensitivity based on our dilution experiments to help guide these decisions.

1.2 The analyzed results provided by AmpliconSuite are organized into three folders containing quite a large number of sub-folders and files. The readiness of the results will be significantly improved by including the code for generating an accompany html report as results output.

This is a very important suggestion as it directly improves the usability and interpretability of the tool. We first wish to mention that one of the goals of the AmpliconRepository interface is that users will be able to upload their output files there directly, and have the html-based summary tables and visualizations in front of them without needing to sift through various output files. To achieve this we have added a couple of important features to AmpliconSuite-pipeline and AmpliconRepository.org.

First, before undertaking an analysis, users can create an empty project on AmpliconRepository.org. Created projects may be publicly viewable or private (default). On the project page, a project owner can gather the project's UUID and and secret key. When users run AmpliconSuite-pipeline.py they can provide an optional argument "--upload" which enables them to directly send results files to the site into the existing project. They can then see the summary tables directly on the project page of AmpliconRepository.

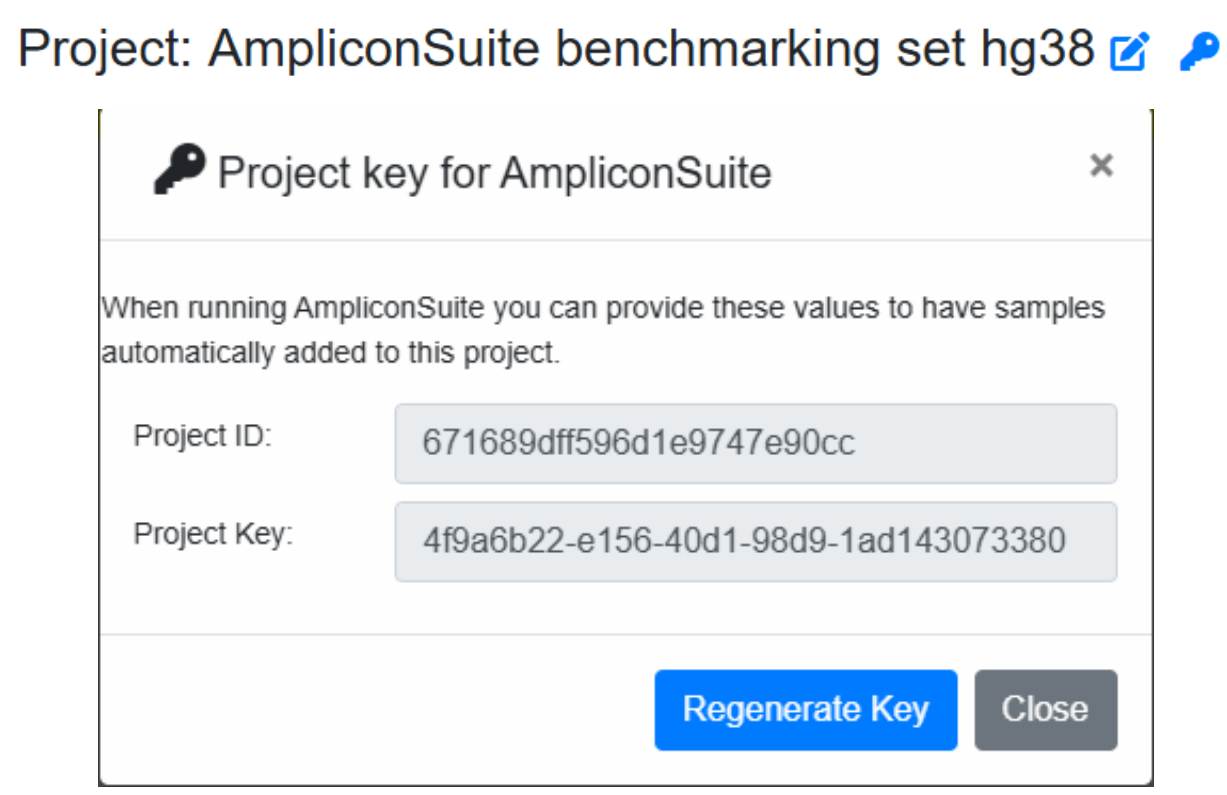

Optional AmpliconRepository upload arguments

- `--upload` : Sets condition to upload after run completes.
- `--project_uuid {str}` : Project UUID where sample(s) will be uploaded to (required if `--upload` set). Obtained from key icon on AmpRepo project page.
- `--project_key {str}` : Project secret key (required if `--upload` set). Obtained from key icon on AmpRepo project page.
- `--username {str}` : Username on AmpliconRepository
- `--upload_server local|dev|prod` : This is a developer argument to assist with debugging upload functionality. The default is `prod` which is AmpliconRepository.org.

For users who do not necessarily want the web interface to manage and view consolidated results, and prefer a consolidated tabular output, we have added an optional argument to AmpliconClassifier, `--make_results_table` which creates a consolidated table

"_results_table.tsv" of the most important annotations created by AmpliconClassifier, one row per focal amplification.

2. The AmpliconRepository portal

I have been exploring the AmpliconRepository database generated by this study. The interoperability of AmpliconRepository makes this database unique and distinct from the eccDNA Atlas database (https://lcbb.swjtu.edu.cn/eccDNAatlas/index.html). Both eccDNA Atlas and AmpliconRepository database mainly focus on providing static results on focal amplicon and eccDNA (including ecDNA).

One important value of studying focal amplification and ecDNA in cancer is related to their potential role in cancer prognosis. It will be relevant and highly useful to include the prognostic value of ecDNA genes, amplicon classification, and ecContext in the database.
The Human Protein Atlas (e.g.https://www.proteinatlas.org/ENSG00000146648-EGFR/pathology/urothelial+cancer) have provided excellent examples for interactively exploring the prognostic value of genes.

Particularly, since the study has already analyzed over 2000 tumor samples from PCAWG and TCGA, it should be feasible to explore the prognostic potential of the ecDNA genes and focal amplicons.

Response: We thank the reviewer for this excellent suggestion. Indeed, it would be ideal that our platform provide some interactive embedded analysis to users. Indeed, as we have >15,000 unique samples present on the site now, some kind of analysis which also leverages the large scale of data would be ideal.

Certain annotations with prognostic significance, such as survival data, do not readily translate to automated web-based analysis when combining cohorts with heterogeneous construction or annotation standards. The covariates required for meaningful survival analysis vary substantially across cohorts, precluding standardized automated approaches. However, such analyses have been comprehensively addressed in the primary publications that generated these datasets (Kim et al., Nature Genetics, 2020; Chapman et al., Nature Genetics, 2023; Bailey et al., Nature, 2024; Lv et al., Cancer Discovery, 2025).

However, the reviewer's comment inspired us to develop a new feature for AmpliconRepository that not only facilitates surveying ecDNA but also enables identification of potential gene dependencies created by ecDNA. We implemented a "Co-amplification Graph" utility that analyzes gene co-amplification patterns across the ecDNA in samples selected from one or more projects. This tool uses AmpliconClassifier-generated gene lists as input to construct a network where nodes represent genes and edges represent the number of samples exhibiting co-amplification of gene pairs on ecDNA. Edges are annotated to specify whether genes co-amplify on the same ecDNA element or distinct ecDNA elements, and further distinguish whether co-

amplified genes derive from a single genomic interval, multiple intervals, or segments from different chromosomes.

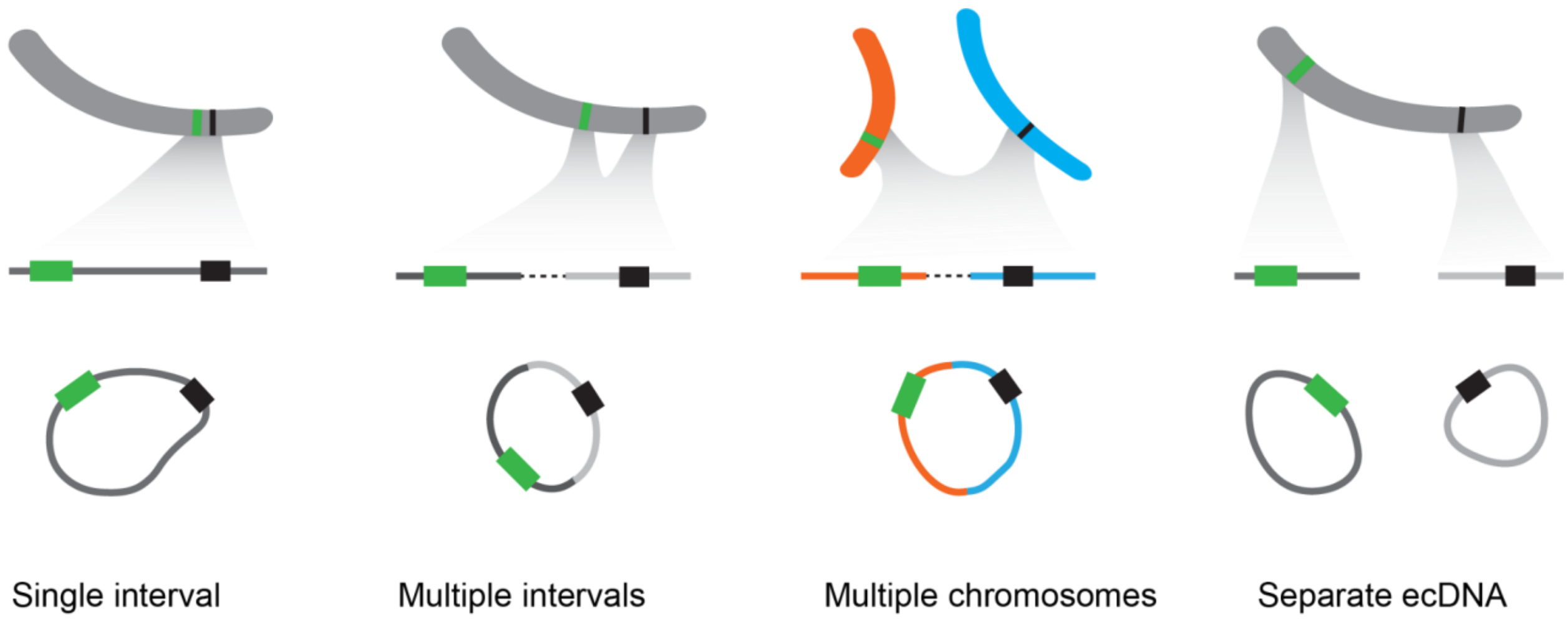

As output it generates a tabular form where rows are each pair of genes that has an ecDNA co-amplification, and columns represent counts of the types of co-amplifications.

We built this into AmpliconRepository.org using Neo4j as the backend graph database constrution & search tool, with Cytoscape.js as the method for visualization. To use the co-amplification graph utility, users can select one or more projects to combine with the same reference genome build.

Coamplification Graph Analysis

Select projects to include in your coamplification graph analysis. All selected projects must be of the same reference group - either (GRCh37, hg19) or (GRCh38, GRCh38_viral).

☑	TCGA	TCGA samples processed by Verhaak and Kim labs, filtered for coverage, duplicate samples that appeared in PCAWG, and other criteria as described in Kim et al., 2024. Manifest file availble in project download. Sample naming convention is [TCGA tumor sample ID]_MN_[matched normal ID]	Public	GRCh37	101
☑	PCAWG	PCAWG samples processed by Verhaak and Kim labs, filtered for coverage, duplicates and other criteria as described in Kim et al, 2024.	Public	GRCh37	2,095

Showing 1 to 5 of 5 entries (filtered from 49 total entries) Previous 1 Next

Generate Coamplification Graph

Users then click “Generate Co-amplification Graph” at the bottom of the page and will be directed to a Cytoscape.js-powered visualizer and tabular view where they can search gene names and see what genes are co-amplified alongside the searched gene (Supplementary Figure 7d).

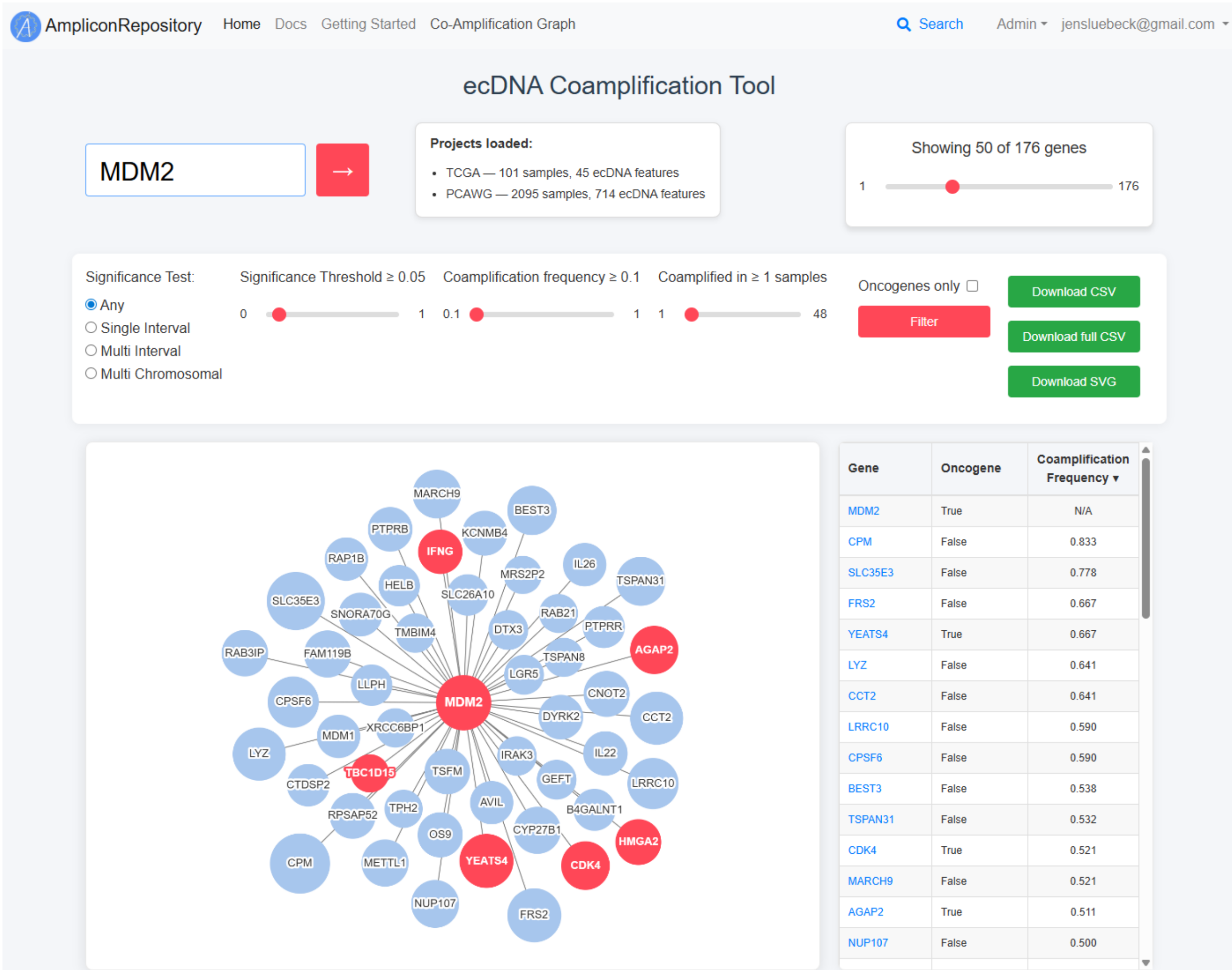

We made a number of fascinating discoveries by combining large-scale pan-cancer cohorts on AmpliconRepository.org and analyzing their gene co-amplification profiles. Namely, one of the most frequent co-amplifications of oncogenes mediated by ecDNA was the CDK4-MDM2 co-amplification, where ~72% of such co-amplifications were mediated solely by ecDNA. A second observation was in the role of ecDNA support genes - in particular we observed many chromatin remodeler oncogenes co-amplified alongside other driver oncogenes (e.g. MDM2 - YEATS4, PAK1 - NSD3).

Consequently, these results have become the subject of the first part of our revised Figure 3 (a-e).

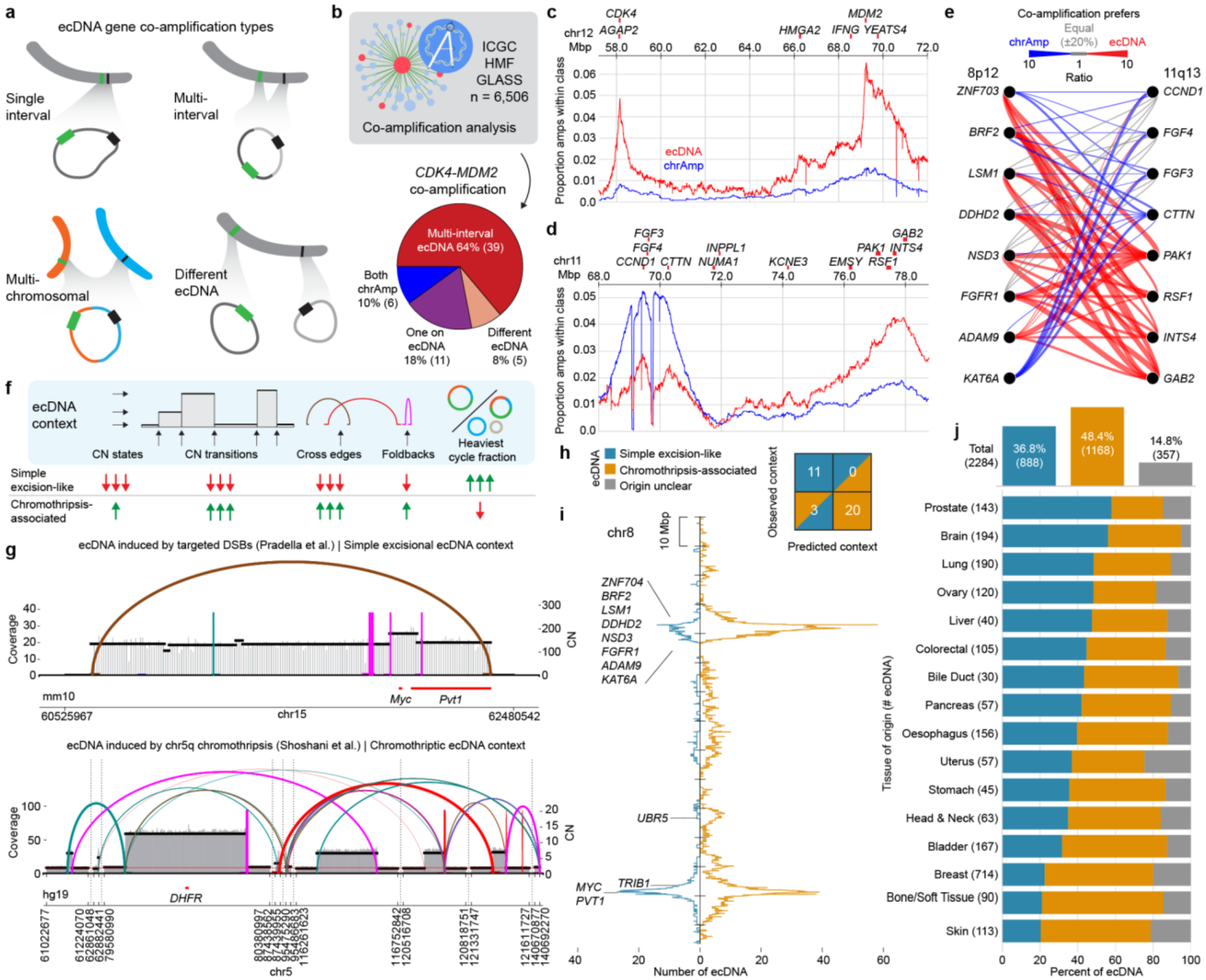

Lines 266-347 of the results section highlight these findings. In brief they report that,

- CDK4-MDM2 co-amplification (61 samples): 64% on multi-interval ecDNA, demonstrating ecDNA's outsize role in driving this potent co-amplification, 8% on separate ecDNA.
- Regional analysis of chr12q14-15 showed ecDNA selectively excludes intergenic regions between CDK4-MDM2 while incorporating neighboring oncogenes (YEATS4, AGAP2)
- chr11q13 analysis revealed mechanistic preferences: CCND1/FGF3/FGF4 relatively enriched in chromosomal amplifications while PAK1/RSF1/INTS4/GAB2 preferentially amplified by ecDNA, suggesting distinct selective advantages for chromatin remodeling and signaling genes
- Multi-chromosomal co-amplification between chr8p12 and chr11q13 oncogene clusters showed potential ecDNA-mediated functional synergy between signaling pathway components and chromatin remodeling machinery.

3. Classification of ecDNA and HSR with AA

ecDNA can subsequently integrate into the chromosome as homogeneously staining regions (HSRs). One limitation and concern about AA is regarding the accuracy or correctness of distinguishing the ecDNA from the other two classes of HSR-like amplifications (CNC and linear). It is unclear to me that whether this issued has been addressed and improved in the updated AA. According to the results provided in Figure 2c, it doesn't seems that this has been resolved in the new version of AA.

Response: We appreciate the reviewer raising this important point about AmpliconArchitect's (AA) classification of ecDNA versus HSR-like amplifications. We acknowledge that AA cannot definitively distinguish between ecDNA and HSRs that are ecDNA-derived (i.e., ecDNAs that have integrated into chromosomes). This limitation reflects a fundamental challenge in the field: even when ecDNA integrates into chromosomes, the amplified structural variants that suggest circularity, and an ecDNA origin continue to exist. By contrast, the structural variants that anchor them into potentially multiple locations in the genome only require two unamplified endpoints, making them more difficult to detect. In fact, no existing bioinformatic tool takes WGS data and reliably predicts integration status of ecDNA-derived amplifications.

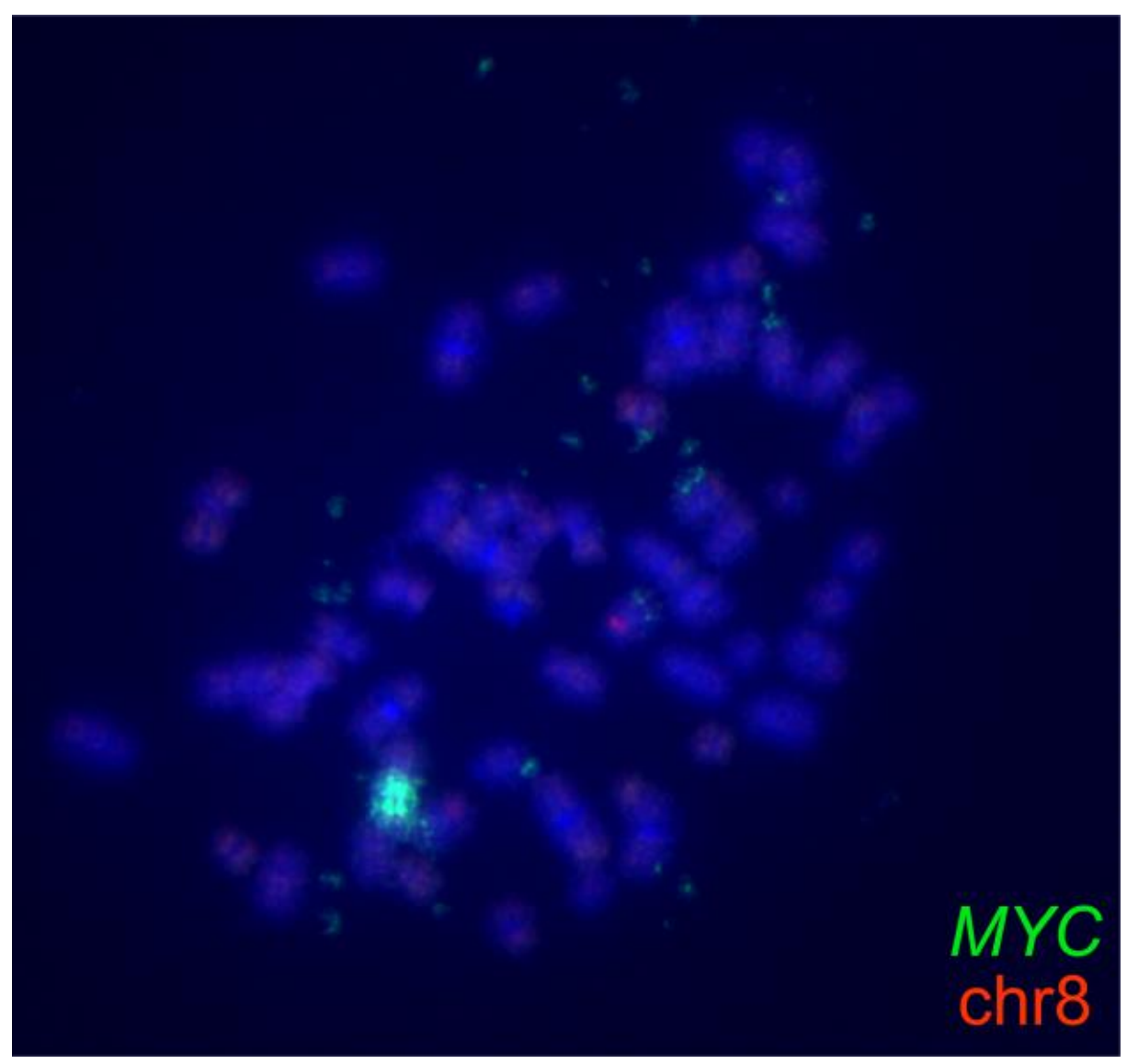

However, tumor heterogeneity actually simplifies this classification challenge in practice. When ecDNA integrates and selective pressures expand the HSR-bearing clone, the non-integrated subclone is unlikely to be entirely lost. This is strikingly illustrated in drug treatment experiments targeting ecDNA-borne oncogenes (Nathanson et al., Science, 2014), whereby and EGFR ecDNA was seemingly replaced by ecDNA-derived HSR of the same structure when erlotinib was applied, yet the original ecDNA reappeared after drug removal. The kind of heterogeneity sparking this process is actually visible in the metaphase FISH experiments we performed. For instance, the ecDNA MYC amplification in DMS273 shows up in some cells as a combination of both ecDNA and an HSR form (pictured at right) - located off the native chromosome, which is likely ecDNA-derived.

One alternative explanation would be an independent amplification carried on HSR of the same oncogene. The focal amplification of MYC is too complex to resolve in DMS273, however, in cell line CCFSTTG1, we also noticed large non-native CDK6 HSRs alongside the ecDNA in 3 of 19 metaphase images. Through analysis of the directly matched WGS data we found that the focal amplification of CDK6 was explained unambiguously by an ecDNA cycle - suggesting no other modes of focal amplification at that location. This suggests these HSR events are latent

integrations of ecDNA as ecDNA-derived HSR on a non-native chromosome. We have added Supplementary Figure 4 which illustrates this finding.

This has important implications for interpreting AA+AC classifications. In patient tumors (as opposed to heavily clonal cell lines), the distinction between "pure ecDNA" and "ecDNA-derived HSR" is less critical. The typical evolutionary trajectory may first involve ecDNA as the dominant form, followed by integration events producing HSRs in cell subsets. Labeling such amplifications as "ecDNA" remains biologically accurate.

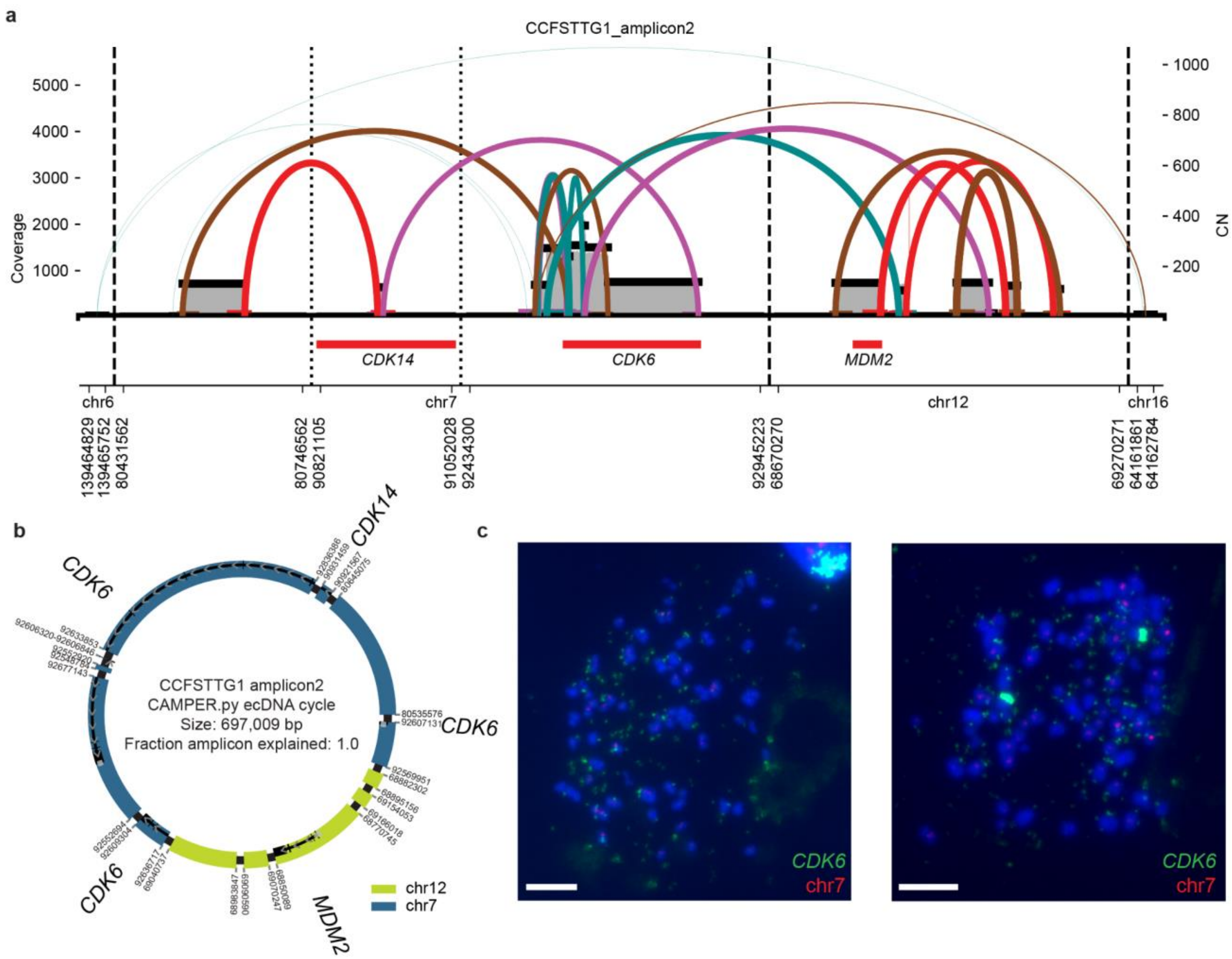

This relationship between ecDNA and non-native HSRs representing integrated ecDNA amplifications requires special interpretation in comparisons of metaphase FISH and WGS results. When evaluating the performance of ecDNA detection on cell lines, where re-integration is more common due to lowered selective pressures for ecDNA, we cannot confidently say that a non-native HSR in FISH data is evidence the ecDNA we confidently find in WGS is false. We have re-worked Figure 2 to better clarify the way the FISH validation data has been segregated.

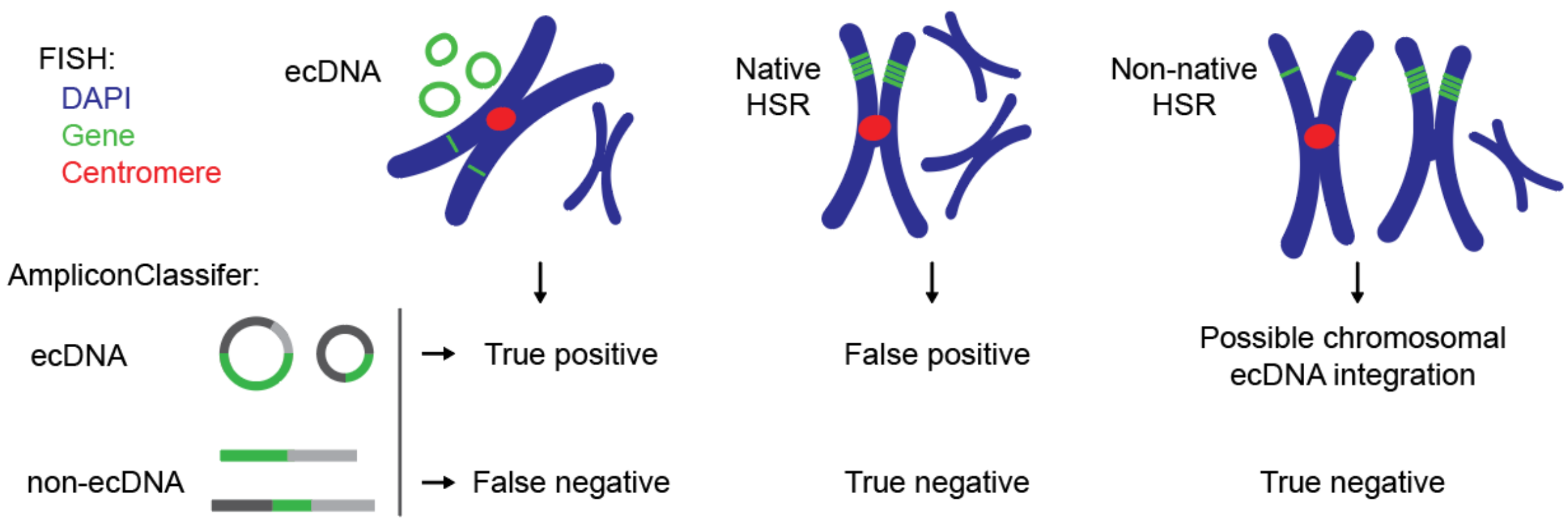

Our data provides compelling evidence that many non-native HSRs detected by FISH are likely ecDNA-derived. In cases where WGS predicted ecDNA but FISH showed HSR (Supplementary Table 2), we observed that 20 of 25 chromosomally localizable HSRs were located on non-native chromosomes, while only 5 were on the native chromosome. This high frequency of non-native integration is highly characteristic of ecDNA-derived HSRs, as ecDNA can integrate at random genomic locations (Luebeck et al., Nature Communications 2020; Song et al., Cancer Discovery, 2022). In contrast, amplifications formed through purely chromosomal mechanisms (such as BFB cycles or tandem duplications detected as CNC structures) would be more likely to remain on their native chromosome.

Essentially, AA detects the "fossil record" of ecDNA - the structural signatures of circular architecture and complex rearrangements that persist even after chromosomal integration. Identifying ecDNA-origin amplifications remains highly informative because these retain the potential for ecDNA re-emergence and carry associated therapeutic challenges. While we acknowledge AA cannot resolve whether an ecDNA has become HSR-dominant, the biological plasticity of ecDNA, the non-native integration evidence, and the heterogeneous selective pressures in patient tumors (where ecDNA cells likely persist and can be re-selected; Nathanson et al., Science, 2014) suggest our classifications capture the functionally relevant distinction between ecDNA-origin and purely chromosomal amplification mechanisms.

We have added the following to the Discussion section (lines 443-455): “There are natural limitations to the detection of focal amplifications, imposed both by sequencing technology and tumor biology. An important limitation of short-read WGS is distinguishing extrachromosomal ecDNA from chromosomally integrated, ecDNA-derived HSR. Integration junctions require only two unamplified endpoints, while amplified structural variants indicating circular origin persist after integration, driving the identification of ecDNA-like genome reconstructions. However, our FISH validation reveals that HSRs co-occurring with WGS-predicted ecDNA predominantly localize to non-native chromosomes (20 of 25 cases), consistent with ecDNA-derived integration rather than purely chromosomal mechanisms. Moreover, dynamics of tumor heterogeneity suggests ecDNA-bearing subclones may persist even when HSRs dominate, and can re-emerge when selective pressures shift. Consequently, identifying ecDNA-origin amplifications, whether currently extrachromosomal or integrated, remains biologically and clinically informative, as these

amplifications retain the potential for ecDNA re-emergence and the associated therapeutic challenges of extrachromosomal amplification."

4. ecContext

The conceptional introduction of ecDNA context based on their potential generative mechanisms is excellent. This will facilitate the better interpretation, study and understanding of ecDNA in cancer. The methods section has provided very detail description of the six types of ecDNA contexts based on their generative mechanisms. However, one major concern regarding the imputation of ecDNAs into such contexts is based on the assumption that the ecDNA in the tumor tissue (cells) is homogenous. Biologically, cancer cells and their genomes are highly heterogeneous in tumor tissues, particularly in tumors with advanced stages. For example, the ecDNA defined as heavily rearranged unichromosomal (HRUC, Figure 3f) could also be explained by cancer cell heterogeneity, comprising several ecDNAs within this genomic region of the tumor cells. If one inspecting the coverage of ecDNA amplicon, the depth diversity among the different genomic blocks within the ecDNA can be an indicative of heterogeneity.

Response: We thank the reviewer for their compliment towards the utility of the ecDNA context work and for raising this important point about potential confounding from tumor heterogeneity or post-formation rearrangements. We agree that distinguishing chromothripsis-derived ecDNA from ecDNA that have undergone recombination or accumulated complexity after formation is a key challenge. However, ecContext addresses this concern through several design features, which we have adjusted our methods section to better reflect.

First, ecContext implements a preprocessing step that identifies when the primary circular structure is formed by a single everted (head-to-tail) SV edge, the signatures of excisional formation. When detected, genome segments falling *inside* this founding cycle are excluded from the structural complexity metrics used for classification. This filtering step removes post-formation rearrangements that may have occurred through recombination, structural evolution, or clonal heterogeneity after the initial excision event, allowing ecContext to focus on the genomic context at the time of ecDNA formation rather than subsequent evolutionary changes. However, if such a signature is not found, then SVs unrelated to the origin will add noise.

Second, the "simple circular" categories explicitly accommodate some degree of internal complexity (allowing up to 3 cross edges, T/N ratio <4, and up to 6 CN states within the ecDNA). This provides tolerance for moderate post-formation changes while still distinguishing these from the extreme oscillating copy number states and high edge crossing characteristic of chromothripsis.

First, we turned to previously published data whereby ecDNA was generated experimentally in model systems from a known mechanism of formation and deployed ecContext. Two landmark studies provided this data; Shoshani et al., Nature, 2021 and Pradella et al., Nature, 2025. In the Pradella study, ecDNA was induced by engineering a circular structure from the cleaved DNA between inserted loxP sites, containing murine Myc or Mdm2. Cre recombinase excised and

circularized the intervening DNA through double strand breaks, creating an oncogene-bearing megabase-scale head-to-tail ecDNA characteristic of simply-structured ecDNA which was allowed to develop in the mouse tumor. In contrast, Shoshani et al. observed spontaneous ecDNA formation in methotrexate-selected HeLa cells through chromothripsis, where chromosomal regions underwent catastrophic shattering into multiple fragments that were then reassembled into complex circular amplicons in a PARP- and DNA-PKcs-dependent manner. The Shoshani study authors documented in the figures and supplement of their study that with their genomic and cytogenetic analysis, the majority of ecDNA formations occurred through chromothripsis, however a few were also marked as extrusion.

We found that among the samples generated from non-chromothriptic mechanisms, namely excisional ecDNA formation, 11/11 (8/8 Pradella samples, 3/3 Shoshani samples) were classified as having a the "simple circular simple background" context label - which is a non-chromothriptic category. Among samples from the Shoshani study with ecDNA capturing segments of chr5 20/23 (87%) were given context labels associated with chromothripsis ("heavily rearranged unichromosomal", "simple circular complex background"). These results are available in revised Figure 3h and in Supplementary Table 8. We note that none of the ecDNA experimentally generated from excisional mechanisms were labeled as chromothripsis-associated, even though there was some evidence of internal SVs (e.g. revised Figure 3g), like the reviewer suggested may occur.

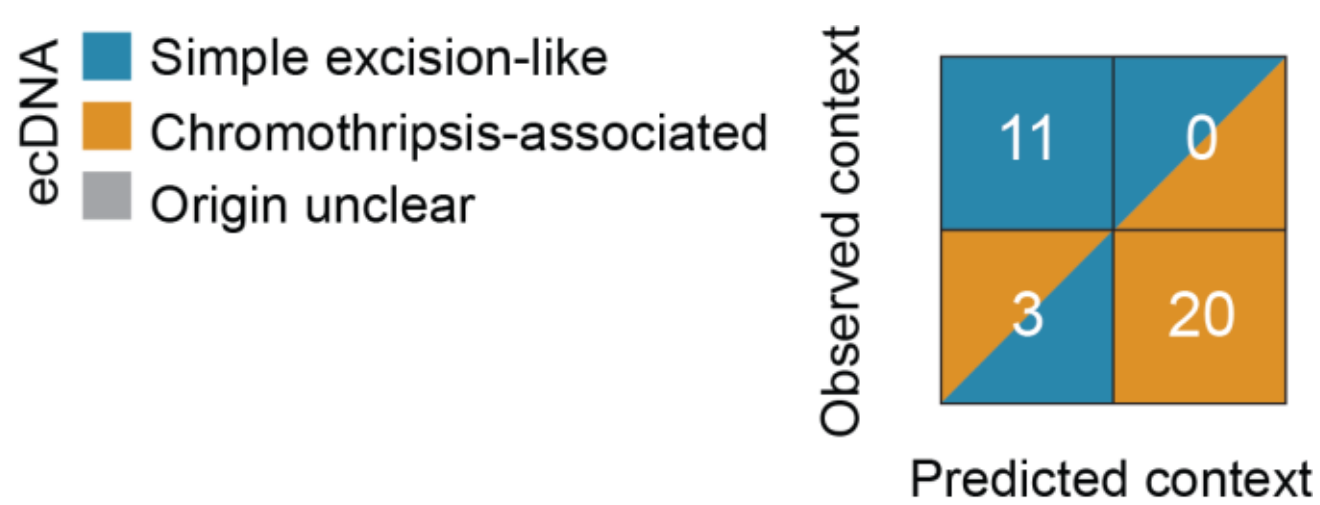

While ecContext's filtering approach mitigates many confounding effects from post-formation events, we acknowledge that extreme heterogeneity or extensive post-formation recombination could potentially be misclassified as chromothriptic origin in edge cases. However, our validation against experimentally-generated ecDNA suggests that the structural signatures of chromothriptic formation (high edge crossing, oscillating copy numbers) remain distinguishable from typical levels of post-formation complexity. We have added to the Discussion on lines 419-421: "An important caveat of this method is that chromothripsis-associated classifications could in some cases capture simple ecDNA that has undergone extreme additional rearrangement or recombination post-formation."

Minor Comments:

5.1: Figure 2c, the figure legend should include the information that these 43 cancer cell lines are different from those benchmarking cancer cells used in Figure 2a-b. The results in Figure 2c do suggest that the current AA pipeline still cannot distinguish ecDNA from HSRs.

Response: This is a good distinction to make - we have updated the figure caption accordingly to show the timing dataset is not the same as the FISH validation dataset:

“**a)** Runtimes of AmpliconArchitect (AA) on 81 cancer cell line samples using the originally published 2019 version of AA and AA version 1.5.r2. Diagonal line indicates y=x. **b)** Speedup of AA on 81 cancer cell line samples using timing values in panel a. Horizontal grey line at y=1 indicates same speed as AA 2019.”

We have generally completely updated Figure 2, and in our response to the reviewer’s comment #3 above which answers the HSR point. We have distinguished the types of HSR found by FISH, and whether the HSR is likely ecDNA-derived. Subsequently, and as mentioned in response to comment #3, we have also re-iterated the difficulty to distinguish ecDNA-derived HSRs with WGS in Discussion (lines 443-455).

5.2: Regarding the truncated genes on ecDNA, PVT1 is provided as an example (Figure 3i and j). However, since PVT1 is co-amplified with MYC, the data provided is not sufficient to conclude that the truncated ecDNA gene has oncogenic effect and positive selection effect. Similarly, the truncated SYT1 gene on ecDNA is within the same ecDNA amplicon of MDM2 (check from the AmpliconRepository database).

Response: We have removed this analysis from the study as it was not strong enough on its own to suggest oncogenic effects of truncated genes. We note that some authors on this manuscript in addition with other colleagues recently published a separate study which analyzes in depth the role of PVT1 as a fusion partner on ecDNA (Yi, Zhang et al., Cell, 2026; PMID: 41506267), and better demonstrates how rearrangements that truncate PVT1 can then allow it to join to other fusion partners.

5.3: Furthermore, some of the truncated ecDNA genes, e.g. RPSAP52, MGAT4C, ecDNA gene could not be found in the AmpliconRepository.

Response: We thank the reviewer for pointing this out. In initial iterations of AmpliconSuite and AmpliconRepository, genes found to be truncated in the sample were not propagated to the list of searchable genes within a project on the site. However, because truncated genes can still have clinical and scientific importance we have now removed this limitation and now genes, regardless of truncation status can be searched there.

5.4: Supplementary Figure 4b. one typo in the illustrative heavily rearranged ecDNA from single chromosome (“p” should be “g”).

Response: We apologies for the confusion with the orientation of the letters which was rotated to show the segment had been reversed in orientation. Revised Supplementary Figure 4b (revised Supplementary Figure 8b) previously showed segments d, f, e, h coming together in a rearranged circular order, and we realize the rotation of the text on the panel was likely confusing. We have changed segment ‘d’ to ‘c’ in the resulting ecDNA structure.

Remarks on code availability:

We have checked all the codes provided in GitHub and tested them with in-house WGS data from human cancers. There are enough and very detail instructions for using the codes. These codes are usable resource for the community and outperform the older version of AA/AC.

Response: We thank the reviewer again for their examination of the tools and methods and for trying the method on their own data. We are very happy to hear that the tool was readily usable based on the documentation, and that the newer version outperformed the older version of the method.

Reviewer #2:

Remarks to the Author:
We would like to thank the authors for carefully addressing our concerns. We are convinced that AmpliconClassifier will make a great contribution to the field. We have no further comments.

Response: We agree and sincerely thank the reviewer again for their efforts. Their feedback has significantly improved the manuscript.

Reviewer #3:

Remarks to the Author:
In this revision, the authors have addressed majority of my previous concerns and comments. However, one of my major concerns for AmpliconSuite is still its novelty and significance as a novel and disruptive method for the field.

Just to be fair to the authors for the great amounts of work and effort that have been investigated for the study, I have carried out code validation and pipeline parsing for AmpliconSuite. The whole pipeline could be executed successfully and operated smoothly using the codes provided via GitHub. So, from a reproducibility point of view, I can confirm that pipelines are reproducible. Agreed with the authors' claim regarding the mapping speed of the AA, we observed some improvement but not dramatic. Another implementation that the current AmpliconSuite has installed is a more detailed stratification of the ecDNA context. I would hope that the authors could provide more biological and cancer insights in the revision. However, the current applicational demonstration of AmpliconSuite with ecDNA gene co-amplification and tissue-type based cancer stratification still belong to the nature of descriptive observations, rather than mechanical insights that can only be achieved with AmpliconSuite, substantially lacking validations.

However, I see the study more valuable from a resource point of view instead of a significantly improved or novel method for the ecDNA and cancer biology field. The aggregated pipelines used by AmpliconSuite enable one-stop identification and characterization of focal amplification from WGS data. The online repository as commended before is another useful resource for the field. The current repository contains 8772 samples from 14 projects. However, the online repository is more a platform or app for visualization of AA outputs. The analytical and applicational values of the online platform are still limited.

Remarks on code availability:
The codes provided contain enough details for reproducibility. We are able to run the code locally and successfully.

Response: We thank the reviewer for their thorough engagement with our work, including the time invested in code validation and pipeline testing. We are glad to hear the reviewer's

confirmation that AmpliconSuite-pipeline runs reproducibly and that AmpliconArchitect's runtime improvements are observable. We address the remaining concerns below.

Novelty and biological insights:

We respectfully would like to offer a different perspective on the manuscript's contributions to the field. While several tools detect focal amplification structures including ecDNA from WGS data, AmpliconClassifier offers a complementary rule-based approach for systematic classification, and with the new FISH validation set has been tested on a larger set of cytogenetically validated models than any other related tool. The embedded ecContext method is, to our knowledge, the only available approach for inferring ecDNA formation mechanism (chromothriptic versus excisional) from WGS data. The ecContext method in particular addresses a question, “how did this ecDNA form?” - that no other existing tool directly attempts. Validation of formation mechanism classification is inherently challenging because experimental ground-truth datasets are extremely limited; we used all available published launcher model WGS data (Pradella et al., 2025 and Shoshani et al., 2021) for validation, which to our knowledge represents the most comprehensive validation possible given the current state of the field.

We agree that the gene co-amplification and tissue-type stratification findings are observational rather than experimental, mechanistic interrogations, and have not claimed otherwise in the manuscript. We believe these observations are nonetheless valuable: the finding that ecDNA mediates 72% of CDK4-MDM2 co-amplifications, for example, has direct clinical implications for therapeutic strategy that were not previously recognized. Bioinformatic tools like AmpliconSuite are not only hypothesis testing machines, but also hypothesis-generating. The systematic framework AmpliconSuite provides is precisely what enables the field to prioritize which co-amplification events warrant mechanistic follow-up - a function that is distinct from, and complementary to, experimental validation. Generating mechanistic validation for each observation would require dedicated experimental studies that are well beyond the scope of a methods paper, but our work provides the systematic framework that enables such follow-up studies on well-chosen, interesting targets it identifies.

Regarding AmpliconRepository.org:

We respectfully push back on the argument minimizing AmpliconRepository.org as primarily a visualization platform. AmpliconRepository.org has been in active development and use for over five years, and represents the only community-editable repository of focal amplification calls with versioned project management, programmatic upload, and analytical utilities (including the co-amplification graph utility demonstrated in Figure 2). The co-amplification function in particular allows embedded analysis of gene co-amplifications in the website, with exportable analysis results. AmpliconRepository is becoming the de facto community resource for ecDNA data sharing, with over 15,000 samples across 30+ projects. We also apologize for the discrepancy with the 8,772 samples/14 projects numbers the reviewer observed, that was due to a temporary issue that affected project counts listed on the site, causing an undercounting, which has since been resolved.

While individual features may exist in other platforms, AmpliconRepository.org's combination of standardized output sharing, user-editable projects, and integrated analytical tools is unique. We would also note that critical infrastructure in genomics such as cBioPortal, UCSC Genome Browser, or IGV is often not characterized as "disruptive" in itself, yet enables disruptive science through the analyses it makes possible. AmpliconRepository.org fills an analogous role for the ecDNA field.

We again appreciate the reviewer's thoughtful engagement and agree that future work will continue to expand the biological and methodological insights enabled by AmpliconSuite. We believe the current manuscript represents a substantial and valuable contribution to the field with broad interest to cancer investigators. It provides a reproducible, validated, broadly-adopted toolkit with novel classification methods, paired with a community resource that has already enabled dozens of published studies. We hope the reviewer will agree this combination provides important value to the field.
